# Supplementary material for: Targeting Cancer Stem Cells in Triple-Negative Breast Cancer
Source: Cancers (Basel). 2019 Jul 9;11(7):965. doi: 10.3390/cancers11070965 (PMC6678244; doi:10.3390/cancers11070965)
Supplement: Supplementary file 1 [file cancers-11-00965-s001.pdf]

Supplementary Methods

Gene set enrichment analysis (GSEA) of triple negative breast cancer (TNBC)

We obtained the primary gene expression data of breast cancer patients and breast cancer cell lines from Gene Expression Omnibus (GEO; http://www.ncbi.nlm.nih.gov/geo/) and analyzed the gene signature with Gene Set Enrichment Analysis (GSEA), as described in our previous report [1]. Briefly, the clinical data of breast cancer patients were obtained from the GEO database under series accession no. GSE27447, and human breast cancer cell line data were obtained from GSE32474. We utilized GEO2R (https://www.ncbi.nlm.nih.gov/geo/geo2r/) to compare TNBC and non-TNBC groups to identify differentially expressed genes. From GSE27447, we compared the gene expression profiles of five TNBC patient tumors with those of fourteen non-TNBC patient tumors, and we identified 1972 genes that were differentially expressed between TNBC and non-TNBC patients (p-value<5E-02, Supplementary Table 1). From the GSE32474 dataset, we compared the genomic signature of TNBC cell lines (BT549, HS578T, and MDA-MB-231) with that of non-TNBC cell lines (MCF7 and T47D), and we identified a total of 653 annotated genes that were differentially expressed between human TNBC and non-TNBC cell lines (p-value<1E-08, Supplementary Table 2). Then, GSEA of the ranked gene list was performed using the Java implementation of GSEA obtained from http://www.broadinstitute.org/gsea/ (5000 permutations, minimum term size of 15, and maximum term size of 500). The entire gene lists for TNBC patients as well as for TNBC cell lines were pre-ranked based on the mean fold change. The analysis included gene sets from MSigDB pathways and C2: curated gene sets (c2.all.v6.2.symbols.gmt), and an FDR q-value < 0.05 was set as the significance threshold.

Supplementary Tables

Supplementary Table 1. Differentially expressed gene list of TNBC patient tumors (n=5) versus non-TNBC patient tumors (n=14).

Supplementary Table 2. Differentially expressed gene list of TNBC cell lines (BT549, HS578T, and MDA-MB-231) versus non-TNBC cell lines (MCF7 and T47D).

Supplementary Tables are attached as excel files.

References

1. Kim J.-H.; Park S.-Y; Jun Y.; Kim J.-Y.; Nam J.-S. Roles of Wnt Target Genes in the Journey of Cancer Stem Cells. *International Journal of Molecular Sciences* **2017**, *18*, pii: E1604

Supplementary Table 1. Differentially expressed gene list of TNBC patient tumors (n=5) versus non-TNBC patient tumors (n=14).

#	ID	Gene.symb	TNBC group	non-TNBC group	logFC	P.Value	Gene.title
1	8103706	AADAT	2.272663	-3.5244	1.667202	0.0348422	aminoadipate aminotransferase
2	7989953	AAGAB	-2.9889457	-2.4408	-0.59936	0.0075418	alpha- and gamma-adaptin binding protein
3	8052798	AAK1	2.220929	-3.5981	0.519726	0.0387047	AP2 associated kinase 1
4	8100478	AASDH	-2.1427178	-3.7078	-0.60192	0.0452934	aminoadipate-semialdehyde dehydrogenase
5	8070708	AATBC	2.2450704	-3.5638	0.524513	0.0368558	apoptosis associated transcript in bladder cancer
6	8131682	ABCB5	-2.1401391	-3.7114	-1.18796	0.0455271	ATP binding cassette subfamily B member 5
7	7975607	ACOT4	-2.8771048	-2.6158	-1.45266	0.0096492	acyl-CoA thioesterase 4
8	7952503	ACRV1	2.1522735	-3.6945	0.541704	0.0444369	acrosomal vesicle protein 1
9	8137474	ACTR3B	2.497213	-3.196	0.932199	0.0218645	ARP3 actin related protein 3 homolog B
10	8092002	ACTRT3	2.1043293	-3.761	0.477936	0.0488877	actin related protein T3
11	7955535	ACVR1B	-3.4919358	-1.6429	-0.7804	0.0024384	activin A receptor type 1B
12	8066431	ADA	2.5949572	-3.0493	0.613998	0.0177718	adenosine deaminase
13	7983191	ADAL	-3.6757514	-1.3502	-1.13951	0.0016052	adenosine deaminase like
14	8115490	ADAM19	2.2082558	-3.616	0.784916	0.0397089	ADAM metallopeptidase domain 19
15	7979927	ADAM20	-2.5968716	-3.0464	-0.97852	0.0176993	ADAM metallopeptidase domain 20
16	7979925	ADAM20P	-3.1352823	-2.21	-1.10773	0.0054469	ADAM metallopeptidase domain 20 pseudogene 1
17	7990736	ADAMTS7	2.7036207	-2.8839	0.471716	0.0140756	ADAM metallopeptidase with thrombospondin type 1 motif 7
18	7952752	ADAMTS8	2.267395	-3.5319	0.373428	0.0352187	ADAM metallopeptidase with thrombospondin type 1 motif 8
19	8088560	ADAMTS9	2.223479	-3.5944	0.814403	0.0385054	ADAM metallopeptidase with thrombospondin type 1 motif 9
20	8132667	ADCY1	-2.3826722	-3.3652	-2.13113	0.027783	adenylate cyclase 1
21	7999079	ADCY9	-2.178401	-3.658	-0.68505	0.0421698	adenylate cyclase 9
22	8052882	ADD2	2.3413871	-3.4253	0.920914	0.0302602	adducin 2
23	8157777	ADGRD2	3.0434101	-2.3551	0.753347	0.006684	adhesion G protein-coupled receptor D2
24	7996064	ADGRG5	2.6787531	-2.9219	0.748457	0.0148507	adhesion G protein-coupled receptor G5
25	7902565	ADGRL2	3.1775711	-2.143	1.370978	0.0049553	adhesion G protein-coupled receptor L2
26	8106827	ADGRV1	-2.9017974	-2.5773	-2.19419	0.0091399	adhesion G protein-coupled receptor V1
27	7928882	ADIRF	-2.6158137	-3.0177	-1.52148	0.0169976	adipogenesis regulatory factor
28	7928471	ADK	-2.4563235	-3.2568	-0.54747	0.0238268	adenosine kinase
29	8005134	ADORA2B	2.5798829	-3.072	0.501918	0.0183519	adenosine A2b receptor
30	7925550	ADSS	-2.2428494	-3.5669	-0.80113	0.0370224	adenylosuccinate synthase
31	7977273	ADSSL1	-3.924199	-0.9566	-1.24256	0.0009105	adenylosuccinate synthase like 1
32	8106429	AGGF1	-2.3379787	-3.4303	-0.45223	0.0304736	angiogenic factor with G-patch and FHA domains 1
33	8130628	AGPAT4	2.2572734	-3.5464	1.030554	0.0359524	1-acylglycerol-3-phosphate O-acyltransferase 4
34	8138381	AGR2	-3.034222	-2.3696	-4.76127	0.0068217	anterior gradient 2, protein disulphide isomerase family member
35	8138392	AGR3	-2.8541838	-2.6514	-3.82108	0.0101462	anterior gradient 3, protein disulphide isomerase family member
36	7896822	AGRN	-3.6272845	-1.4273	-1.2464	0.0017926	agrin
37	8169492	AGTR2	-2.350242	-3.4125	-0.79261	0.0297122	angiotensin II receptor type 2
38	8049737	AGXT	2.147625	-3.701	0.561296	0.0448517	alanine-glyoxylate aminotransferase
39	8122396	AIG1	-2.8242038	-2.698	-0.85036	0.0108335	androgen induced 1
40	8011912	AIPL1	2.428713	-3.2976	0.446297	0.0252438	aryl hydrocarbon receptor interacting protein like 1
41	8112458	AK6///TAF	-2.1319152	-3.7228	-0.37853	0.0462798	adenylate kinase 6///TATA-box binding protein associated factor 9
42	8177635	AK6///TAF	-2.1319152	-3.7228	-0.37853	0.0462798	adenylate kinase 6///TATA-box binding protein associated factor 9
43	8164766	AK8	-2.2637759	-3.5371	-0.58121	0.0354794	adenylate kinase 8
44	8128777	AK9	-4.3419818	-0.3051	-6.19443	0.0003509	adenylate kinase 9
45	8128788	AK9	-2.2081295	-3.6162	-0.79441	0.0397191	adenylate kinase 9
46	7975066	AKAP5	-2.1550402	-3.6907	-1.10602	0.0441917	A-kinase anchoring protein 5
47	8163569	AKNA	2.3821898	-3.3659	0.831578	0.0278109	AT-hook transcription factor
48	7904084	AKR7A2P1	-2.0949187	-3.7739	-0.5781	0.0498074	aldo-keto reductase family 7 member A2 pseudogene 1
49	7913146	AKR7A3	-2.1430004	-3.7074	-1.01223	0.0452679	aldo-keto reductase family 7 member A3
50	7994737	ALDOA	-2.6232516	-3.0064	-0.86986	0.0167294	aldolase, fructose-bisphosphate A
51	7936923	ALDOAP2	-2.1384024	-3.7138	-0.76954	0.0456851	aldolase, fructose-bisphosphate A pseudogene 2
52	7969228	ALG11///U	-2.5976779	-3.0452	-0.49494	0.0176689	ALG11, alpha-1,2-mannosyltransferase///UTP14, small subunit proces
53	8141757	ALKBH4	2.769381	-2.7828	0.541324	0.0122074	alkB homolog 4, lysine demethylase
54	8040053	ALLC	3.2541557	-2.0215	1.017958	0.0041729	allantoicase
55	8004221	ALOX12	2.1981782	-3.6302	0.453382	0.0405245	arachidonate 12-lipoxygenase, 12S type
56	8012309	ALOX12B	2.2575772	-3.546	0.439605	0.0359302	arachidonate 12-lipoxygenase, 12R type
57	8001477	AMFR	-2.6542221	-2.9593	-0.77238	0.0156549	autocrine motility factor receptor
58	8090852	AMOTL2	-3.0848012	-2.2898	-0.7071	0.0060961	angiomotin like 2
59	8090866	ANAPC13	-3.0338613	-2.3702	-0.95255	0.0068272	anaphase promoting complex subunit 13
60	8033892	ANGPTL6	2.1121698	-3.7501	0.470955	0.0481332	angiopoietin like 6
61	8150439	ANK1	2.3629479	-3.394	0.46731	0.028942	ankyrin 1
62	7984227	ANKDD1A	3.0400253	-2.3605	0.497822	0.0067344	ankyrin repeat and death domain containing 1A
63	8060949	ANKEF1	-5.0439266	0.7419	-1.35595	0.000072	ankyrin repeat and EF-hand domain containing 1
64	7943943	ANKK1	2.3528345	-3.4087	0.400823	0.0295535	ankyrin repeat and kinase domain containing 1
65	7934979	ANKRD1	2.1881199	-3.6443	0.493973	0.0413538	ankyrin repeat domain 1
66	7927033	ANKRD30	-3.1461142	-2.1929	-6.27253	0.0053167	ankyrin repeat domain 30A
67	8069499	ANKRD30	-3.3952732	-1.7969	-3.55391	0.0030354	ankyrin repeat domain 30B
68	8112672	ANKRD31	2.493414	-3.2017	0.877722	0.0220402	ankyrin repeat domain 31
69	8016725	ANKRD40	-2.728627	-2.8455	-0.59072	0.0133352	ankyrin repeat domain 40
70	7942858	ANKRD42	-2.3024942	-3.4816	-1.0422	0.0327798	ankyrin repeat domain 42
71	7965686	ANKS1B	-2.3903619	-3.3539	-1.47992	0.027343	ankyrin repeat and sterile alpha motif domain containing 1B

72	7993815	ANKS4B	2.6442458	-2.9745	0.669602	0.0159937	ankyrin repeat and sterile alpha motif domain containing 4B
73	7938951	ANO5	-2.3424692	-3.4238	-1.12212	0.0301927	anoctamin 5
74	8049799	ANO7	2.1350723	-3.7184	0.452277	0.0459895	anoctamin 7
75	7905283	ANXA9	-2.4597308	-3.2517	-2.26573	0.0236572	annexin A9
76	8034084	AP1M2	-2.7490871	-2.814	-2.50749	0.0127571	adaptor related protein complex 1 mu 2 subunit
77	8161618	APBA1	3.5176519	-1.6019	0.960177	0.0023002	amyloid beta precursor protein binding family A member 1
78	8099982	APBB2	-2.5988325	-3.0434	-0.94924	0.0176255	amyloid beta precursor protein binding family B member 2
79	7897632	APITD1-CC	-2.8836635	-2.6055	-1.1645	0.0095113	APITD1-CORT readthrough///apoptosis-inducing, TAF9-like domain 1
80	7966003	APPL2	-2.2425228	-3.5674	-0.50044	0.037047	adaptor protein, phosphotyrosine interacting with PH domain and le
81	7942774	AQP11	-2.122294	-3.7362	-1.1444	0.0471746	aquaporin 11
82	7987325	AQR	-2.9339698	-2.527	-0.54104	0.0085152	aquarius intron-binding spliceosomal factor
83	8167998	AR	-2.5116395	-3.1745	-1.98042	0.0212091	androgen receptor
84	8133021	ARAFP2	2.3752435	-3.376	0.446955	0.0282144	ARAF pseudogene 2
85	8139804	ARAFP2	2.3752435	-3.376	0.446955	0.0282144	ARAF pseudogene 2
86	7962904	ARF3	-2.5975476	-3.0453	-0.51749	0.0176738	ADP ribosylation factor 3
87	8088339	ARF4	-2.4728572	-3.2323	-0.54835	0.0230142	ADP ribosylation factor 4
88	8122279	ARFGEF3	-5.8304428	1.8217	-1.93978	0.0000129	ARFGEF family member 3
89	8097813	ARFIP1	-3.1000184	-2.2658	-0.62602	0.0058929	ADP ribosylation factor interacting protein 1
90	7946201	ARFIP2	-2.5586605	-3.104	-0.67522	0.019199	ADP ribosylation factor interacting protein 2
91	8045563	ARHGAP15	2.1832787	-3.6511	1.17502	0.0417584	Rho GTPase activating protein 15
92	7921752	ARHGAP30	2.3182758	-3.4588	0.788445	0.0317349	Rho GTPase activating protein 30
93	8081838	ARHGAP31	2.2641881	-3.5365	0.731037	0.0354496	Rho GTPase activating protein 31
94	7952641	ARHGAP32	-2.209819	-3.6138	-0.69274	0.0395838	Rho GTPase activating protein 32
95	8062532	ARHGAP40	-2.5174616	-3.1658	-2.15779	0.0209499	Rho GTPase activating protein 40
96	7964436	ARHGAP9	2.4224611	-3.3068	0.90279	0.0255754	Rho GTPase activating protein 9
97	7944560	ARHGEF12	-2.2883407	-3.5019	-0.53691	0.0337438	Rho guanine nucleotide exchange factor 12
98	7897154	ARHGEF16	-3.3584979	-1.8555	-0.9732	0.0032985	Rho guanine nucleotide exchange factor 16
99	8106210	ARHGEF28	-2.3961807	-3.3454	-0.87502	0.0270144	Rho guanine nucleotide exchange factor 28
100	8096682	ARHGEF38	-2.7721862	-2.7784	-2.81453	0.0121333	Rho guanine nucleotide exchange factor 38
101	8168781	ARL13A	2.2024182	-3.6242	0.701607	0.0401795	ADP ribosylation factor like GTPase 13A
102	7995193	ARMC5	2.302131	-3.4821	0.454134	0.0328042	armadillo repeat containing 5
103	8122818	ARMT1	-3.1253059	-2.2258	-1.72407	0.0055696	acidic residue methyltransferase 1
104	7988963	ARPP19	-2.6176075	-3.015	-0.48835	0.0169326	cAMP regulated phosphoprotein 19
105	7910494	ARV1///AR	-2.1460972	-3.7031	-0.63449	0.0449888	ARV1 homolog, fatty acid homeostasis modulator///ARV1 homolog,
106	7933341	ASAH2B///	-2.7287155	-2.8454	-0.73624	0.0133326	N-acylsphingosine amidohydrolase 2B///N-acylsphingosine amidohy
107	7933597	ASAH2B///	-2.6877952	-2.9081	-0.73703	0.0145643	N-acylsphingosine amidohydrolase 2B///N-acylsphingosine amidohy
108	7927599	ASAH2B///	-2.5887009	-3.0587	-0.67476	0.0180104	N-acylsphingosine amidohydrolase 2B///N-acylsphingosine amidohy
109	8026024	ASNA1	-2.6952897	-2.8966	-0.57937	0.0143309	arsA arsenite transporter, ATP-binding, homolog 1 (bacterial)
110	8090664	ASTE1	-2.5356473	-3.1386	-1.01035	0.0201593	asteroid homolog 1 (Drosophila)
111	7955425	ATF1	-2.287929	-3.5025	-0.56576	0.0337723	activating transcription factor 1
112	8049271	ATG16L1	-2.3661643	-3.3893	-0.54874	0.02875	autophagy related 16 like 1
113	8048120	ATIC	-2.5196916	-3.1624	-0.6127	0.0208514	5-aminoimidazole-4-carboxamide ribonucleotide formyltransferase/II
114	7986789	ATP10A	-2.1692	-3.6709	-0.85022	0.0429558	ATPase phospholipid transporting 10A (putative)
115	7907160	ATP1B1	-2.4717828	-3.2338	-0.93742	0.0230662	ATPase Na+/K+ transporting subunit beta 1
116	7997593	ATP2C2	-4.0240968	-0.7994	-1.66455	0.0007247	ATPase secretory pathway Ca2+ transporting 2
117	7963713	ATP5G2	-2.3507804	-3.4117	-0.35939	0.0296792	ATP synthase, H+ transporting, mitochondrial Fo complex subunit C2
118	8037037	ATP5SL	-2.5314968	-3.1448	-0.56814	0.0203372	ATP5S like
119	8143221	ATP6V0A4	2.3247245	-3.4495	2.171831	0.0313168	ATPase H+ transporting V0 subunit a4
120	8074251	ATP6V1E1	-2.1112179	-3.7515	-0.39749	0.0482243	ATPase H+ transporting V1 subunit E1
121	7971731	ATP7B	-4.1762064	-0.5616	-1.47318	0.000512	ATPase copper transporting beta
122	8100026	ATP8A1	2.8898665	-2.5959	1.154224	0.0093827	ATPase phospholipid transporting 8A1
123	8023497	ATP8B1	-2.6715703	-2.9329	-1.08277	0.015082	ATPase phospholipid transporting 8B1
124	8142096	ATXN7L1	2.60747	-3.0303	0.58551	0.0173034	ataxin 7 like 1
125	8142098	ATXN7L1	2.216131	-3.6049	0.622058	0.0390821	ataxin 7 like 1
126	7964575	AVIL	-3.2288622	-2.0617	-1.06946	0.0044169	advillin
127	8132188	AVL9	-2.7640632	-2.791	-0.55734	0.0123492	AVL9 cell migration associated
128	7907719	AXDND1	-2.6323269	-2.9926	-1.8063	0.0164075	axonemal dynein light chain domain containing 1
129	8141374	AZGP1	-2.9005357	-2.5792	-2.34764	0.0091653	alpha-2-glycoprotein 1, zinc-binding
130	8091780	B3GALNT1	-2.0971333	-3.7709	-0.79006	0.0495896	beta-1,3-N-acetylgalactosaminyltransferase 1 (globoside blood group
131	7968370	B3GLCT	-2.483989	-3.2157	-0.6221	0.0224819	beta 3-glucosyltransferase
132	8084206	B3GNT5	2.8862183	-2.6016	1.482984	0.0094581	UDP-GlcNAc:betaGal beta-1,3-N-acetylglucosaminyltransferase 5
133	8049075	B3GNT7	2.2616669	-3.5401	0.658002	0.0356322	UDP-GlcNAc:betaGal beta-1,3-N-acetylglucosaminyltransferase 7
134	8128247	BACH2	2.4179987	-3.3133	1.715049	0.0258146	BTB domain and CNC homolog 2
135	8096617	BANK1	3.0924167	-2.2778	2.123757	0.0059936	B-cell scaffold protein with ankyrin repeats 1
136	7964203	BAZ2A	-2.4151626	-3.3175	-0.46541	0.0259677	bromodomain adjacent to zinc finger domain 2A
137	7975676	BBOF1	-3.0419877	-2.3574	-0.98223	0.0067051	basal body orientation factor 1
138	8097252	BBS12	-2.2384355	-3.5732	-0.53517	0.0373557	Bardet-Biedl syndrome 12
139	7984686	BBS4	-2.7774875	-2.7703	-0.71116	0.0119943	Bardet-Biedl syndrome 4
140	8046147	BBS5	-2.6165273	-3.0166	-0.73107	0.0169717	Bardet-Biedl syndrome 5
141	8135422	BCAP29	-2.130656	-3.7246	-0.69208	0.046396	B-cell receptor-associated protein 29
142	8063417	BCAS4	-2.3252676	-3.4487	-1.00813	0.0312819	breast carcinoma amplified sequence 4
143	7963139	BCDIN3D	-2.1666876	-3.6744	-0.57105	0.0431728	BCDIN3 domain containing RNA methyltransferase
144	7917338	BCL10	-2.1523453	-3.6944	-0.39604	0.0444305	B-cell CLL/lymphoma 10

145	8052399	BCL11A	3.3687551	-1.8392	2.315409	0.003223	B-cell CLL/lymphoma 11A
146	7981242	BCL11B	2.346752	-3.4175	1.005218	0.0299271	B-cell CLL/lymphoma 11B
147	7996281	BEAN1	2.129717	-3.7259	0.412036	0.0464828	brain expressed, associated with NEDD4, 1
148	8015715	BECN1	-3.0760505	-2.3036	-0.56573	0.0062161	beclin 1
149	8169009	BEX4	-2.579441	-3.0727	-0.73996	0.0183692	brain expressed X-linked 4
150	7961891	BHLHE41	2.1800437	-3.6557	1.105775	0.0420309	basic helix-loop-helix family member e41
151	7954717	BICD1	2.0996645	-3.7674	0.480853	0.0493417	BICD cargo adaptor 1
152	8054945	BIN1	2.1067736	-3.7576	0.861427	0.0486514	bridging integrator 1
153	8144625	BLK	2.2102155	-3.6132	3.556865	0.0395521	BLK proto-oncogene, Src family tyrosine kinase
154	7956013	BLOC1S1-I	-2.1246406	-3.7329	-0.52946	0.0469549	BLOC1S1-RDH5 readthrough///biogenesis of lysosomal organelles co
155	8132515	BLVRA	-3.3796469	-1.8218	-1.17509	0.0031446	biliverdin reductase A
156	8036913	BLVRB	-2.15358	-3.6927	-0.68344	0.0443209	biliverdin reductase B
157	7987454	BMF	4.159089	-0.5883	0.873347	0.0005324	Bcl2 modifying factor
158	7979241	BMP4	-3.589626	-1.4873	-0.94318	0.001953	bone morphogenetic protein 4
159	8180361	BMP8B	2.2814012	-3.5119	0.557538	0.034226	bone morphogenetic protein 8b
160	7989323	BNIP2	-2.2313641	-3.5833	-0.38751	0.0378953	BCL2 interacting protein 2
161	8145454	BNIP3L	-2.231308	-3.5833	-0.52549	0.0378996	BCL2 interacting protein 3 like
162	8081686	BOC	2.1470123	-3.7018	1.328845	0.0449066	BOC cell adhesion associated, oncogene regulated
163	8062444	BPI	3.0232413	-2.3869	1.925202	0.00699	bactericidal/permeability-increasing protein
164	8061883	BPIFA1	2.275391	-3.5205	0.879779	0.0346488	BPI fold containing family A member 1
165	8075600	BPIFC	2.4728332	-3.2323	0.83537	0.0230154	BPI fold containing family C
166	8134454	BRI3	2.192329	-3.6384	0.445816	0.0410049	brain protein I3
167	8085287	BRK1	2.4545532	-3.2594	0.73589	0.0239153	BRICK1, SCAR/WAVE actin nucleating complex subunit
168	7909931	BROX	-2.4224167	-3.3068	-0.50436	0.0255778	BRO1 domain and CAAX motif containing
169	8077595	BRPF1	2.1987323	-3.6294	0.447163	0.0404793	bromodomain and PHD finger containing 1
170	7902965	BTBD8	-2.3012339	-3.4834	-0.8148	0.0328646	BTB domain containing 8
171	7951604	BTG4	2.1047836	-3.7603	0.874941	0.0488437	BTG anti-proliferation factor 4
172	8089519	BTLA	3.7313302	-1.2619	1.605129	0.0014142	B and T lymphocyte associated
173	8047339	BZW1	-2.5601838	-3.1017	-0.60484	0.019137	basic leucine zipper and W2 domains 1
174	7934185	C10orf54	2.4082886	-3.3276	0.684499	0.0263423	chromosome 10 open reading frame 54
175	7955777	C12orf10	-2.5779019	-3.075	-0.49146	0.0184295	chromosome 12 open reading frame 10
176	7957467	C12orf29	-2.9961127	-2.4295	-0.6513	0.0074231	chromosome 12 open reading frame 29
177	7977331	C14orf79	-3.2615506	-2.0097	-1.05247	0.0041041	chromosome 14 open reading frame 79
178	7977868	C14orf93	-2.4276441	-3.2991	-0.58529	0.0253002	chromosome 14 open reading frame 93
179	7986239	C15orf32	-3.4526158	-1.7056	-0.79461	0.0026659	chromosome 15 open reading frame 32
180	7991357	C15orf38-/	-2.3571931	-3.4023	-0.43489	0.0292885	C15orf38-AP3S2 readthrough///adaptor related protein complex 3 si
181	7991367	C15orf38-/	-2.9189107	-2.5505	-0.71363	0.0088023	C15orf38-AP3S2 readthrough///actin-related protein 2/3 complex inl
182	7982593	C15orf54	-2.2710048	-3.5268	-1.00345	0.0349603	chromosome 15 open reading frame 54
183	8002992	C16orf46	-2.163512	-3.6788	-0.78903	0.0434484	chromosome 16 open reading frame 46
184	8000702	C16orf54	2.2760381	-3.5196	1.242003	0.034603	chromosome 16 open reading frame 54
185	8003601	C16orf54	2.2760381	-3.5196	1.242003	0.034603	chromosome 16 open reading frame 54
186	8001048	C16orf58	-2.1273527	-3.7291	-0.52778	0.0467022	chromosome 16 open reading frame 58
187	7923974	C1orf132//	-2.2861028	-3.5051	-0.8137	0.0338987	chromosome 1 open reading frame 132///microRNA 29c
188	7897753	C1orf167	3.6625666	-1.3712	0.501728	0.0016542	chromosome 1 open reading frame 167
189	7905324	C1orf56	-3.1962742	-2.1134	-1.04638	0.004752	chromosome 1 open reading frame 56
190	8111443	C1QTNF3-,	-2.1040527	-3.7613	-1.19245	0.0489145	C1QTNF3-AMACR readthrough (NMD candidate)///C1q and tumor r
191	8065244	C20orf78	2.2749264	-3.5212	0.568775	0.0346817	chromosome 20 open reading frame 78
192	8071745	C22orf15	2.613113	-3.0218	0.671847	0.0170961	chromosome 22 open reading frame 15
193	8080847	C3orf14	-5.4828542	1.3577	-2.14349	0.0000273	chromosome 3 open reading frame 14
194	8081645	C3orf52	-4.3173017	-0.3431	-1.54682	0.0003711	chromosome 3 open reading frame 52
195	8083223	C3orf58	2.5652537	-3.0941	0.922127	0.018932	chromosome 3 open reading frame 58
196	8097435	C4orf33	-2.1856762	-3.6478	-0.67599	0.0415576	chromosome 4 open reading frame 33
197	8107194	C5orf30	-2.3469268	-3.4173	-0.81801	0.0299163	chromosome 5 open reading frame 30
198	8110518	C5orf60	-2.5178346	-3.1652	-0.53497	0.0209334	chromosome 5 open reading frame 60
199	8108192	C5orf66	2.2388084	-3.5727	0.480244	0.0373274	chromosome 5 open reading frame 66
200	8123463	C6orf120	-2.3255069	-3.4483	-0.44852	0.0312665	chromosome 6 open reading frame 120
201	8126446	C6orf226	2.1771711	-3.6597	0.48703	0.0422741	chromosome 6 open reading frame 226
202	8139228	C7orf25	-2.2612493	-3.5407	-0.50787	0.0356625	chromosome 7 open reading frame 25
203	8136535	C7orf55-LU	2.1247307	-3.7328	0.340903	0.0469465	C7orf55-LUC7L2 readthrough///formation of mitochondrial complex
204	8158539	C9orf106	-3.6118747	-1.4518	-1.88113	0.0018566	chromosome 9 open reading frame 106
205	8163181	C9orf152	-2.6335623	-2.9908	-2.46422	0.0163641	chromosome 9 open reading frame 152
206	8162039	C9orf64	-2.6705258	-2.9345	-0.80055	0.0151159	chromosome 9 open reading frame 64
207	8163348	C9orf84	-2.3238263	-3.4508	-1.35552	0.0313748	chromosome 9 open reading frame 84
208	7989501	CA12	-2.8647268	-2.635	-2.29305	0.0099146	carbonic anhydrase 12
209	8150978	CA8	-4.2172153	-0.4979	-2.25529	0.0004663	carbonic anhydrase 8
210	7949843	CABP2	3.040699	-2.3594	0.654007	0.0067243	calcium binding protein 2
211	8034643	CACNA1A	2.5035249	-3.1866	0.488816	0.0215754	calcium voltage-gated channel subunit alpha1 A
212	8172580	CACNA1F	-2.9737958	-2.4646	-4.70958	0.0077987	calcium voltage-gated channel subunit alpha1 F
213	8008409	CACNA1G	2.2414888	-3.5689	0.445551	0.0371248	calcium voltage-gated channel subunit alpha1 G
214	8073152	CACNA1I	2.4488291	-3.2679	0.386338	0.0242039	calcium voltage-gated channel subunit alpha1 I
215	8009334	CACNG4	-3.4265151	-1.7471	-1.98694	0.0028282	calcium voltage-gated channel auxiliary subunit gamma 4
216	8081036	CADM2	-2.7114498	-2.8719	-2.08981	0.0138397	cell adhesion molecule 2
217	8142585	CADPS2	-2.1231377	-3.735	-1.03402	0.0470955	calcium dependent secretion activator 2

218	8092468	CAMK2N2	2.3680204	-3.3866	0.418915	0.0286397	calcium/calmodulin dependent protein kinase II inhibitor 2
219	8107307	CAMK4	2.5427472	-3.1279	1.278866	0.0198583	calcium/calmodulin dependent protein kinase IV
220	8117054	CAP2	-3.0602928	-2.3285	-1.57832	0.0064378	CAP, adenylate cyclase-associated protein, 2 (yeast)
221	8051275	CAPN13	-2.1151138	-3.7461	-2.02412	0.0478527	calpain 13
222	8174527	CAPN6	2.9751502	-2.4625	1.801111	0.0077754	calpain 6
223	7910466	CAPN9	-2.6579297	-2.9537	-2.36086	0.0155308	calpain 9
224	8028172	CAPNS1	-2.1602185	-3.6834	-0.33898	0.043736	calpain small subunit 1
225	7962112	CAPRIN2	3.0145066	-2.4006	0.726327	0.0071267	caprin family member 2
226	8156404	CARD19	-2.8292648	-2.6901	-0.78938	0.0107144	caspase recruitment domain family member 19
227	7996608	CARMIL2	3.4211668	-1.7557	1.121882	0.0028627	capping protein regulator and myosin 1 linker 2
228	7961844	CASC1	-3.3320147	-1.8977	-2.17681	0.0035017	cancer susceptibility candidate 1
229	7912646	CASP9	-2.7657223	-2.7884	-0.51802	0.0123048	caspase 9
230	8063497	CASS4	3.2567309	-2.0174	0.662415	0.0041488	Cas scaffolding protein family member 4
231	7912347	CASZ1	-3.3014862	-1.9462	-0.95049	0.0037513	castor zinc finger 1
232	7912343	CASZ1	-3.1146774	-2.2426	-0.87258	0.0057033	castor zinc finger 1
233	7912316	CASZ1	-3.0475498	-2.3486	-0.89103	0.0066228	castor zinc finger 1
234	8108182	CATSPER3	-2.307099	-3.4749	-2.37496	0.0324717	cation channel sperm associated 3
235	8003425	CBFA2T3	2.7519919	-2.8096	0.565384	0.012677	CBFA2/RUNX1 translocation partner 3
236	8113602	CCDC112	-2.1122585	-3.75	-0.52042	0.0481248	coiled-coil domain containing 112
237	8131871	CCDC126	-3.2295884	-2.0605	-0.90029	0.0044098	coiled-coil domain containing 126
238	8057377	CCDC141	2.2768915	-3.5183	1.735544	0.0345428	coiled-coil domain containing 141
239	8133770	CCDC146	-2.8964212	-2.5856	-0.85573	0.0092486	coiled-coil domain containing 146
240	7944913	CCDC15	-2.3997939	-3.3401	-2.8842	0.0268122	coiled-coil domain containing 15
241	8025868	CCDC159	-2.4674254	-3.2403	-0.68359	0.0232783	coiled-coil domain containing 159
242	8022831	CCDC178	2.1251071	-3.7323	0.807372	0.0469114	coiled-coil domain containing 178
243	8000932	CCDC189	-2.4572793	-3.2554	-1.1666	0.0237791	coiled-coil domain containing 189
244	8089659	CCDC191	-2.9568116	-2.4912	-0.84534	0.0080968	coiled-coil domain containing 191
245	8089988	CCDC58	-2.2810736	-3.5123	-0.42786	0.034249	coiled-coil domain containing 58
246	8115261	CCDC69	2.468435	-3.2388	1.027488	0.023229	coiled-coil domain containing 69
247	8087372	CCDC71	-2.1512745	-3.6959	-1.18463	0.0445257	coiled-coil domain containing 71
248	8052269	CCDC88A	2.6202628	-3.0109	0.808839	0.0168367	coiled-coil domain containing 88A
249	7950804	CCDC89	-2.1109698	-3.7518	-1.06029	0.048248	coiled-coil domain containing 89
250	8099746	CCKAR	2.2796267	-3.5144	0.770279	0.0343503	cholecystokinin A receptor
251	8160879	CCL19	2.6977435	-2.8929	2.467384	0.0142552	C-C motif chemokine ligand 19
252	7942123	CCND1	-2.4877958	-3.21	-1.68265	0.0223025	cyclin D1
253	8112072	CCNO	-2.720682	-2.8577	-1.12086	0.0135663	cyclin O
254	7993664	CCP110	-3.0330919	-2.3714	-1.08623	0.0068389	centriolar coiled-coil protein 110
255	8093294	CCR2	3.1412182	-2.2006	1.708961	0.0053752	C-C motif chemokine receptor 2
256	8079392	CCR2	2.5912757	-3.0548	1.301647	0.0179118	C-C motif chemokine receptor 2
257	8078442	CCR4	2.4630387	-3.2468	1.568663	0.0234936	C-C motif chemokine receptor 4
258	8015031	CCR7	3.2082266	-2.0944	1.980432	0.0046263	C-C motif chemokine receptor 7
259	8112428	CD180	2.8096505	-2.7205	1.182424	0.011183	CD180 molecule
260	7994487	CD19	3.2657838	-2.003	1.739821	0.0040652	CD19 molecule
261	8052916	CD207	2.1147949	-3.7465	1.110956	0.047883	CD207 molecule
262	8027837	CD22	3.023703	-2.3862	1.687428	0.0069828	CD22 molecule
263	7922040	CD247	3.0198175	-2.3923	0.868803	0.0070433	CD247 molecule
264	7949588	CD248	2.1158772	-3.745	0.831893	0.0477802	CD248 molecule
265	7953333	CD27	3.1742836	-2.1483	1.630278	0.0049919	CD27 molecule
266	8030277	CD37	2.4066283	-3.3301	1.06591	0.0264335	CD37 molecule
267	8094240	CD38	2.1158988	-3.745	1.503515	0.0477781	CD38 molecule
268	7952056	CD3D	2.1033552	-3.7623	1.129435	0.0489822	CD3d molecule
269	7944179	CD3E	2.6351339	-2.9884	1.528112	0.0163091	CD3e molecule
270	7909400	CD46	-2.6023028	-3.0382	-0.61779	0.0174954	CD46 molecule
271	7940409	CD5	2.6743346	-2.9287	1.580355	0.0149926	CD5 molecule
272	7899160	CD52	2.5551882	-3.1092	1.184367	0.0193411	CD52 molecule
273	8019478	CD7	2.909568	-2.5651	0.702166	0.0089851	CD7 molecule
274	8161004	CD72	2.2152808	-3.6061	0.615073	0.0391493	CD72 molecule
275	8029136	CD79A	3.3924976	-1.8013	2.358974	0.0030546	CD79a molecule
276	8017511	CD79B	2.7114446	-2.8719	1.349591	0.0138398	CD79b molecule
277	8081564	CD96	2.4390419	-3.2823	1.284835	0.0247047	CD96 molecule
278	8112053	CDC20B	-2.7253961	-2.8505	-3.41276	0.0134287	cell division cycle 20B
279	7924773	CDC42BPA	-2.2571316	-3.5466	-0.77215	0.0359628	CDC42 binding protein kinase alpha
280	8046488	CDCA7	2.2380438	-3.5738	1.722164	0.0373854	cell division cycle associated 7
281	8023710	CDH19	3.7233928	-1.2745	3.639313	0.00144	cadherin 19
282	8105862	CDK7	-2.9310147	-2.5316	-0.51463	0.0085708	cyclin dependent kinase 7
283	8177462	CDK7	-2.9310147	-2.5316	-0.51463	0.0085708	cyclin dependent kinase 7
284	8114171	CDKL3	-3.3035602	-1.9429	-0.82456	0.0037338	cyclin dependent kinase like 3
285	8034075	CDKN2D	2.4687857	-3.2383	0.447028	0.0232119	cyclin dependent kinase inhibitor 2D
286	8029086	CEACAM5	-2.5701895	-3.0866	-2.34645	0.0187344	carcinoembryonic antigen related cell adhesion molecule 5
287	8029098	CEACAM6	-2.8648116	-2.6349	-3.63694	0.0099127	carcinoembryonic antigen related cell adhesion molecule 6
288	7926127	CELF2	2.1833261	-3.6511	1.011728	0.0417545	CUGBP, Elav-like family member 2
289	8076757	CELSR1	-3.4021732	-1.7859	-1.5862	0.0029884	cadherin EGF LAG seven-pass G-type receptor 1
290	8156341	CENPP	-2.1282604	-3.7279	-0.89205	0.0466179	centromere protein P

291	8013015	CENPV	2.5655751	-3.0936	1.51338	0.0189191	centromere protein V
292	8167652	CENPVL2//	2.3916751	-3.352	0.333941	0.0272685	centromere protein V like 2///centromere protein V like 1
293	8172715	CENPVL2//	2.3916751	-3.352	0.333941	0.0272685	centromere protein V like 2///centromere protein V like 1
294	7911870	CEP104	-2.1136286	-3.7481	-0.41474	0.047994	centrosomal protein 104
295	8082827	CEP63	-2.4208334	-3.3092	-0.52409	0.0256624	centrosomal protein 63
296	8129193	CEP85L	3.8556634	-1.0648	1.330106	0.0010648	centrosomal protein 85 like
297	7991546	CERS3	2.4204709	-3.3097	1.13253	0.0256818	ceramide synthase 3
298	8046086	CERS6	-3.4955309	-1.6372	-1.15802	0.0024186	ceramide synthase 6
299	7921862	CFAP126	-2.2347174	-3.5785	-1.43984	0.0376385	cilia and flagella associated protein 126
300	8158081	CFAP157	-2.3748979	-3.3765	-0.98483	0.0282346	cilia and flagella associated protein 157
301	7936201	CFAP43	-2.3914186	-3.3524	-1.56382	0.0272831	cilia and flagella associated protein 43
302	8089606	CFAP44	-2.5702847	-3.0865	-0.76047	0.0187306	cilia and flagella associated protein 44
303	8089596	CFAP44	-2.5509976	-3.1155	-0.83012	0.0195139	cilia and flagella associated protein 44
304	7900639	CFAP57	-2.1766012	-3.6605	-0.88655	0.0423225	cilia and flagella associated protein 57
305	7930320	CFAP58	2.5090417	-3.1784	0.662012	0.0213257	cilia and flagella associated protein 58
306	8134051	CFAP69	-2.9092464	-2.5656	-1.34455	0.0089915	cilia and flagella associated protein 69
307	7934334	CFAP70	-2.3449871	-3.4201	-1.12211	0.0300363	cilia and flagella associated protein 70
308	8179351	CFB	-2.8531712	-2.653	-1.93533	0.0101687	complement factor B
309	8118345	CFB	-2.7887953	-2.7528	-1.85323	0.0117028	complement factor B
310	8178115	CFB	-2.7887953	-2.7528	-1.85323	0.0117028	complement factor B
311	8172333	CFP	2.134694	-3.719	0.59971	0.0460242	complement factor properdin
312	7904883	CHD1L	2.1000112	-3.7669	0.623309	0.0493078	chromodomain helicase DNA binding protein 1 like
313	8146579	CHD7	2.2782018	-3.5165	0.765018	0.0344505	chromodomain helicase DNA binding protein 7
314	8039796	CHMP2A	-2.5182984	-3.1645	-0.71453	0.0209129	charged multivesicular body protein 2A
315	8081055	CHMP2B	-3.0669629	-2.318	-0.6904	0.006343	charged multivesicular body protein 2B
316	8154785	CHMP5	-3.0279193	-2.3795	-0.76787	0.0069178	charged multivesicular body protein 5
317	7950425	CHRDL2	2.5350048	-3.1395	0.75009	0.0201867	chordin like 2
318	7910915	CHRM3	4.8279406	0.4273	2.190008	0.0001168	cholinergic receptor muscarinic 3
319	7945896	CHRNA10	-2.4934702	-3.2016	-0.56737	0.0220376	cholinergic receptor nicotinic alpha 10 subunit
320	7928291	CHST3	3.1005766	-2.2649	1.101982	0.0058856	carbohydrate sulfotransferase 3
321	8002882	CHST6	2.8017542	-2.7328	0.581497	0.0113772	carbohydrate sulfotransferase 6
322	7991386	CIB1	-2.886601	-2.601	-0.73936	0.0094502	calcium and integrin binding 1
323	7993195	CIITA	2.4551113	-3.2586	0.97329	0.0238874	class II major histocompatibility complex transactivator
324	8087739	CISH	-2.1801701	-3.6555	-0.75085	0.0420202	cytokine inducible SH2 containing protein
325	8167603	CLCN5	2.7236092	-2.8532	0.900432	0.0134807	chloride voltage-gated channel 5
326	8134091	CLDN12	-2.350718	-3.4118	-0.68158	0.029683	claudin 12
327	7998898	CLDN6	2.2058456	-3.6194	1.031962	0.0399026	claudin 6
328	8012126	CLDN7	-2.842153	-2.6701	-1.36469	0.0104168	claudin 7
329	8012013	CLEC10A	3.5515223	-1.548	1.240996	0.0021298	C-type lectin domain family 10 member A
330	8026350	CLEC17A	3.1969589	-2.1123	0.93519	0.0047447	C-type lectin domain family 17 member A
331	7960832	CLEC4C	3.3635106	-1.8475	1.339774	0.0032614	C-type lectin domain family 4 member C
332	8102877	CLGN	-4.0079048	-0.8248	-2.6	0.000752	calmegin
333	8133459	CLIP2	2.2593841	-3.5434	0.708322	0.0357983	CAP-Gly domain containing linker protein 2
334	8099368	CLNK	2.8567122	-2.6475	0.73432	0.0100902	cytokine dependent hematopoietic cell linker
335	7992917	CLUAP1	-2.4274975	-3.2994	-0.95552	0.025308	clusterin associated protein 1
336	8110971	CMBL	-2.5531589	-3.1123	-1.54696	0.0194246	carboxymethylenebutenolidase homolog
337	8078405	CMTM7	2.3828839	-3.3649	1.271156	0.0277708	CKLF like MARVEL transmembrane domain containing 7
338	7929732	CNNM1	2.3894409	-3.3553	0.620228	0.0273954	cyclin and CBS domain divalent metal cation transport mediator 1
339	7930194	CNNM2	2.3045457	-3.4786	0.796006	0.0326422	cyclin and CBS domain divalent metal cation transport mediator 2
340	8110589	CNOT6	-2.9233968	-2.5435	-0.61518	0.0087158	CCR4-NOT transcription complex subunit 6
341	7911138	CNST	-2.1670633	-3.6739	-0.6728	0.0431403	consortin, connexin sorting protein
342	8036835	CNTD2	-2.3945686	-3.3478	-4.56602	0.0271051	cyclin N-terminal domain containing 2
343	8137010	CNTNAP2	-2.5830786	-3.0672	-1.31526	0.0182274	contactin associated protein-like 2
344	8139244	COA1	-2.616324	-3.0169	-0.49503	0.0169791	cytochrome c oxidase assembly factor 1 homolog
345	8007272	COASY	-2.7407089	-2.8269	-0.74874	0.0129909	Coenzyme A synthase
346	8127446	COL9A1	2.3439467	-3.4216	1.148536	0.0301009	collagen type IX alpha 1 chain
347	8063970	COL9A3	2.9811848	-2.453	0.651523	0.0076723	collagen type IX alpha 3 chain
348	7988605	COPS2	-2.1093618	-3.754	-0.37722	0.0484022	COP9 signalosome subunit 2
349	7955896	COPZ1	-2.336905	-3.4318	-0.69296	0.0305411	coatomer protein complex subunit zeta 1
350	8047217	COQ10B	-2.9957305	-2.4301	-0.75746	0.0074293	coenzyme Q10B
351	7967072	COQ5	-2.1126712	-3.7494	-0.50935	0.0480854	coenzyme Q5, methyltransferase
352	7993608	COQ7	-2.5830458	-3.0673	-1.03158	0.0182287	coenzyme Q7, hydroxylase
353	7994769	CORO1A	3.9037427	-0.9888	1.502945	0.000954	coronin 1A
354	8162744	CORO2A	-2.261045	-3.541	-0.89004	0.0356774	coronin 2A
355	8003171	COTL1	2.4051105	-3.3323	0.795299	0.0265172	coactosin like F-actin binding protein 1
356	7911085	COX20	-2.8835487	-2.6057	-0.81091	0.0095137	COX20, cytochrome c oxidase assembly factor
357	8001041	COX6A2	2.127747	-3.7286	0.434237	0.0466655	cytochrome c oxidase subunit 6A2
358	8136200	CPA4	2.7094398	-2.875	1.371097	0.0138999	carboxypeptidase A4
359	8151191	CPA6	-3.264813	-2.0046	-2.14006	0.0040741	carboxypeptidase A6
360	8035201	CPAMD8	2.609781	-3.0268	1.639459	0.0172182	C3 and PZP like, alpha-2-macroglobulin domain containing 8
361	8083246	CPB1	-2.5252534	-3.1541	-3.6231	0.0206076	carboxypeptidase B1
362	8098204	CPE	-2.2380461	-3.5738	-0.85367	0.0373852	carboxypeptidase E
363	8090690	CPNE4	-2.5070252	-3.1814	-3.31595	0.0214167	copine 4

364	8126035	CPNE5	3.0993598	-2.2668	0.708914	0.0059015	copine 5
365	8018102	CPSF4L	-2.2655626	-3.5346	-2.92451	0.0353505	cleavage and polyadenylation specific factor 4 like
366	8039937	CPTP	-2.4253126	-3.3026	-0.58965	0.0254237	ceramide-1-phosphate transfer protein
367	7909371	CR1	2.1388064	-3.7133	1.187545	0.0456483	complement component 3b/4b receptor 1 (Knops blood group)
368	7909350	CR2	2.6326352	-2.9922	5.90316	0.0163967	complement component 3d receptor 2
369	7985159	CRABP1	3.1236	-2.2285	3.288241	0.0055909	cellular retinoic acid binding protein 1
370	8143188	CREB3L2	2.1092475	-3.7542	0.589447	0.0484132	cAMP responsive element binding protein 3 like 2
371	7905677	CREB3L4	-2.4147204	-3.3182	-1.77395	0.0259916	cAMP responsive element binding protein 3 like 4
372	7977409	CRIP1	-3.4979967	-1.6332	-2.06111	0.0024051	cysteine rich protein 1
373	7977397	CRIP2	-2.2093212	-3.6145	-0.76727	0.0396236	cysteine rich protein 2
374	8146967	CRISPLD1	2.3829563	-3.3648	1.45972	0.0277667	cysteine rich secretory protein LCCL domain containing 1
375	7966052	CRY1	-2.5551866	-3.1092	-0.49587	0.0193412	cryptochrome circadian clock 1
376	8071997	CRYBB2	2.3646307	-3.3915	0.351866	0.0288414	crystallin beta B2
377	8071989	CRYBB3	2.3115867	-3.4684	0.488005	0.0321739	crystallin beta B3
378	7917037	CRYZ	-2.5332207	-3.1422	-0.76657	0.0202631	crystallin zeta
379	8107887	CSF2	2.446621	-3.2711	0.559405	0.024316	colony stimulating factor 2
380	8165735	CSF2RA	2.1461034	-3.7031	0.732097	0.0449882	colony stimulating factor 2 receptor alpha subunit
381	8176306	CSF2RA	2.1461034	-3.7031	0.732097	0.0449882	colony stimulating factor 2 receptor alpha subunit
382	8072757	CSF2RB	2.6158598	-3.0176	1.136986	0.016996	colony stimulating factor 2 receptor beta common subunit
383	8149574	CSGALNAC	2.2628945	-3.5384	1.055698	0.0355432	chondroitin sulfate N-acetylgalactosaminyltransferase 1
384	8095441	CSN1S2AP	-2.3310672	-3.4403	-2.54657	0.0309107	casein alpha s2-like A, pseudogene
385	8115022	CSNK1A1	-2.6073033	-3.0306	-0.61821	0.0173096	casein kinase 1 alpha 1
386	7971071	CSNK1A1L	2.1916702	-3.6394	0.353461	0.0410593	casein kinase 1 alpha 1 like
387	7963235	CSRNP2	-2.5767792	-3.0767	-0.7263	0.0184736	cysteine and serine rich nuclear protein 2
388	8065388	CST11	-2.4114254	-3.323	-0.70391	0.0261707	cystatin 11
389	8061416	CST7	2.9844666	-2.4478	0.950361	0.0076169	cystatin F
390	7969151	CTAGE10P	-2.9153739	-2.5561	-0.87867	0.0088711	CTAGE family member 10, pseudogene
391	8093456	CTBP1-AS2	-2.18183	-3.6532	-0.5902	0.0418802	CTBP1 antisense RNA 2 (head to head)
392	7964579	CTDSP2	-2.212762	-3.6096	-0.41042	0.0393491	CTD small phosphatase 2
393	7983335	CTDSPL2	-2.5272896	-3.1511	-0.32785	0.0205191	CTD small phosphatase like 2
394	8016878	CUEDC1	-2.9874754	-2.4431	-0.8854	0.0075663	CUE domain containing 1
395	8059393	CUL3	-2.2237954	-3.594	-0.40666	0.0384808	cullin 3
396	8173493	CXCR3	2.1971629	-3.6316	0.947484	0.0406075	C-X-C motif chemokine receptor 3
397	8055465	CXCR4	2.7198872	-2.8589	0.948493	0.0135896	C-X-C motif chemokine receptor 4
398	8173437	CXorf65	3.405519	-1.7806	1.395403	0.0029659	chromosome X open reading frame 65
399	7923516	CYB5R1///(	-2.2183597	-3.6017	-0.57663	0.0389064	cytochrome b5 reductase 1///cytochrome b5 reductase 1
400	8051583	CYP1B1	2.7823921	-2.7627	1.127121	0.011867	cytochrome P450 family 1 subfamily B member 1
401	7935169	CYP2C8	-2.2796874	-3.5143	-2.81092	0.0343461	cytochrome P450 family 2 subfamily C member 8
402	8028991	CYP2S1	2.2271918	-3.5892	0.575151	0.038217	cytochrome P450 family 2 subfamily S member 1
403	8035095	CYP4F11	-2.9287027	-2.5352	-2.17238	0.0086146	cytochrome P450 family 4 subfamily F member 11
404	8018922	CYTH1	2.4806915	-3.2206	0.518706	0.0226384	cytohesin 1
405	8055980	CYTIP	2.6692768	-2.9364	1.230021	0.0151566	cytohesin 1 interacting protein
406	8099132	CYTL1	3.4356089	-1.7327	0.629637	0.0027706	cytokine like 1
407	7977775	DAD1	-2.1656606	-3.6758	-0.38251	0.0432618	defender against cell death 1
408	7969959	DAOA	2.1084133	-3.7553	0.400013	0.0484934	D-amino acid oxidase activator
409	8156199	DAPK1	2.6846254	-2.9129	0.949994	0.0146641	death associated protein kinase 1
410	8098732	DBET///DU	2.4953582	-3.1988	0.519836	0.0219501	D4Z4 binding element transcript (non-protein coding)///double hom
411	8098740	DBET///DU	2.4953582	-3.1988	0.519836	0.0219501	D4Z4 binding element transcript (non-protein coding)///double hom
412	8124196	DCDC2	-2.5846933	-3.0648	-2.42887	0.0181649	doublecortin domain containing 2
413	7947282	DCDC5	-2.4240796	-3.3044	-1.52635	0.0254892	doublecortin domain containing 5
414	7932186	DCLRE1C	2.7387505	-2.83	0.61964	0.0130461	DNA cross-link repair 1C
415	7960320	DCP1B	-2.8962379	-2.5859	-0.76446	0.0092523	decapping mRNA 1B
416	8147830	DCSTAMP	-2.3195054	-3.457	-3.97549	0.0316548	dendrocyte expressed seven transmembrane protein
417	7964466	DCTN2	-2.3439789	-3.4216	-0.4274	0.0300989	dynactin subunit 2
418	8092321	DCUN1D1	-2.3171628	-3.4604	-0.44909	0.0318075	defective in cullin neddylation 1 domain containing 1
419	8000028	DCUN1D3	-2.1972621	-3.6315	-0.64577	0.0405994	defective in cullin neddylation 1 domain containing 3
420	7898211	DDI2///RS(	-2.1179541	-3.7422	-0.40967	0.0475834	DNA damage inducible 1 homolog 2///regulatory solute carrier prot
421	8128843	DDO	-2.9332029	-2.5282	-0.72632	0.0085296	D-aspartate oxidase
422	8117900	DDR1	-2.3482122	-3.4154	-0.63007	0.029837	discoidin domain receptor tyrosine kinase 1
423	8177867	DDR1	-2.3482122	-3.4154	-0.63007	0.029837	discoidin domain receptor tyrosine kinase 1
424	8179184	DDR1	-2.3482122	-3.4154	-0.63007	0.029837	discoidin domain receptor tyrosine kinase 1
425	8054758	DDX11L9//	-2.3921322	-3.3513	-1.09656	0.0272426	DEAD/H-box helicase 11 like 9///DEAD/H-box helicase 11 like 5///DI
426	8166440	DDX53	3.0017113	-2.4207	0.769566	0.0073316	DEAD-box helicase 53
427	8157477	DEC1	3.3568095	-1.8582	0.903873	0.0033111	deleted in esophageal cancer 1
428	8118932	DEF6	2.804228	-2.7289	0.762804	0.011316	DEF6, guanine nucleotide exchange factor
429	8126939	DEFB110	-2.463084	-3.2468	-0.67277	0.0234913	defensin beta 110
430	7981278	DEGS2	-2.3963696	-3.3451	-1.6488	0.0270038	delta 4-desaturase, sphingolipid 2
431	7923131	DENND1B	-2.8554671	-2.6494	-1.15081	0.0101177	DENN domain containing 1B
432	7923141	DENND1B	-2.8166451	-2.7097	-1.1599	0.0110137	DENN domain containing 1B
433	7965760	DEPDC4	-3.461523	-1.6914	-3.93577	0.0026126	DEP domain containing 4
434	8074944	DERL3	2.5089674	-3.1785	1.061992	0.0213291	derlin 3
435	8071179	DGCR6	-2.2743574	-3.522	-0.79055	0.034722	DiGeorge syndrome critical region gene 6
436	7916432	DHCR24	-2.6727655	-2.9311	-1.51348	0.0150433	24-dehydrocholesterol reductase

437	7963020	DHH	2.1547435	-3.6911	0.439706	0.0442179	desert hedgehog
438	8034454	DHPS	-3.7376111	-1.2519	-0.76904	0.0013941	deoxyhypusine synthase
439	8099649	DHX15	-3.1605826	-2.17	-0.58299	0.0051474	DEAH-box helicase 15
440	7909478	DIEXF	-2.1799334	-3.6558	-0.52422	0.0420402	digestive organ expansion factor homolog (zebrafish)
441	8097811	DKFZP434I	2.1441392	-3.7058	0.387445	0.0451651	uncharacterized protein DKFZP434I0714
442	7927631	DKK1	-2.4306473	-3.2947	-4.03876	0.025142	dickkopf WNT signaling pathway inhibitor 1
443	8168179	DLG3	-2.6234538	-3.0061	-0.75664	0.0167221	discs large MAGUK scaffold protein 3
444	8144253	DLGAP2	2.272361	-3.5248	0.390161	0.0348637	DLG associated protein 2
445	7931108	DMBT1	-2.6582114	-2.9533	-3.63236	0.0155214	deleted in malignant brain tumors 1
446	8154034	DMRT1	2.10669	-3.7577	0.624963	0.0486594	doublesex and mab-3 related transcription factor 1
447	7910030	DNAH14	-2.6505274	-2.965	-0.99854	0.0157796	dynein axonemal heavy chain 14
448	7910047	DNAH14	-2.4218356	-3.3077	-1.24003	0.0256088	dynein axonemal heavy chain 14
449	8111019	DNAH5	-3.9464203	-0.9215	-5.49175	0.0008654	dynein axonemal heavy chain 5
450	8057821	DNAH7	-3.0497761	-2.3451	-1.6773	0.0065901	dynein axonemal heavy chain 7
451	7985147	DNAJA4	-2.16927	-3.6708	-0.81459	0.0429498	DnaJ heat shock protein family (Hsp40) member A4
452	7933933	DNAJC12	-2.4244595	-3.3038	-2.19883	0.025469	DnaJ heat shock protein family (Hsp40) member C12
453	8082688	DNAJC13	-2.728756	-2.8453	-0.50451	0.0133314	DnaJ heat shock protein family (Hsp40) member C13
454	7963935	DNAJC14	-2.594422	-3.0501	-0.52433	0.0177921	DnaJ heat shock protein family (Hsp40) member C14
455	7898192	DNAJC16	-4.4393582	-0.1559	-0.76581	0.0002811	DnaJ heat shock protein family (Hsp40) member C16
456	7974190	DNAJC19	-2.5495936	-3.1176	-0.58686	0.0195721	DnaJ heat shock protein family (Hsp40) member C19
457	7955211	DNAJC22	-3.4211708	-1.7557	-4.70241	0.0028627	DnaJ heat shock protein family (Hsp40) member C22
458	8114625	DND1	2.1746756	-3.6632	0.54912	0.0424865	DND microRNA-mediated repression inhibitor 1
459	8070730	DNMT3L	2.4160667	-3.3162	0.752888	0.0259188	DNA methyltransferase 3 like
460	8059222	DNPEP	-2.3575196	-3.4019	-0.4629	0.0292688	aspartyl aminopeptidase
461	8109843	DOCK2	2.2158465	-3.6053	0.979224	0.0391046	dedicator of cytokinesis 2
462	8149638	DOK2	2.3805646	-3.3683	0.751418	0.0279048	docking protein 2
463	8056222	DPP4	2.6046209	-3.0346	1.658572	0.017409	dipeptidyl peptidase 4
464	7953665	DPPA3	3.2558369	-2.0188	0.757416	0.0041572	developmental pluripotency associated 3
465	8051387	DPY30	-2.3665784	-3.3887	-0.53462	0.0287254	dpy-30, histone methyltransferase complex regulatory subunit
466	8005289	DRC3	-2.2930047	-3.4952	-1.15838	0.0334233	dynein regulatory complex subunit 3
467	7951703	DRD2	2.3351142	-3.4344	0.380763	0.0306541	dopamine receptor D2
468	8050846	DTNB	2.4165324	-3.3155	0.550869	0.0258937	dystrobrevin beta
469	8113691	DTWD2	-3.0179958	-2.3951	-0.75273	0.0070718	DTW domain containing 2
470	7958931	DTX1	2.0990427	-3.7682	0.493321	0.0494025	deltex E3 ubiquitin ligase 1
471	7934533	DUSP13	-2.3617741	-3.3957	-0.61154	0.0290124	dual specificity phosphatase 13
472	8116548	DUSP22	2.2619796	-3.5397	0.66041	0.0356095	dual specificity phosphatase 22
473	8049684	DUSP28	-2.4055386	-3.3317	-0.50789	0.0264936	dual specificity phosphatase 28
474	8150076	DUSP4	-2.39774	-3.3431	-1.56518	0.0269269	dual specificity phosphatase 4
475	8098725	DUX4///DU	2.5715042	-3.0847	0.548578	0.0186821	double homeobox 4///double homeobox 1///double homeobox 3///
476	8104122	DUX4///DU	2.2455705	-3.5631	0.438062	0.0368183	double homeobox 4///double homeobox 1///double homeobox 5
477	8046340	DYNC1I2	-2.5703707	-3.0864	-0.4684	0.0187271	dynein cytoplasmic 1 intermediate chain 2
478	8180311	DYNC1I2	-2.2728552	-3.5241	-0.33363	0.0348286	dynein cytoplasmic 1 intermediate chain 2
479	8172035	DYNLT3	-4.2113712	-0.507	-1.13976	0.0004725	dynein light chain Tctex-type 3
480	8065202	DZANK1	-2.8448538	-2.6659	-0.81864	0.0103555	double zinc ribbon and ankyrin repeat domains 1
481	7972336	DZIP1	2.9274817	-2.5371	1.407698	0.0086378	DAZ interacting zinc finger protein 1
482	7978558	EAPP	-2.4932736	-3.2019	-0.54193	0.0220467	E2F associated phosphoprotein
483	8067652	EEF1A2	-3.3219579	-1.9137	-3.0956	0.0035821	eukaryotic translation elongation factor 1 alpha 2
484	8123819	EEF1E1-BL	-2.3913816	-3.3524	-0.62353	0.0272852	EEF1E1-BLOC1S5 readthrough (NMD candidate)///eukaryotic translat
485	7993946	EEF2K	-2.1393565	-3.7125	-0.53304	0.0455982	eukaryotic elongation factor 2 kinase
486	7999304	EEF2KMT	-2.3465822	-3.4178	-0.51779	0.0299376	eukaryotic elongation factor 2 lysine methyltransferase
487	7911096	EFCAB2	-3.2696481	-1.9969	-1.39862	0.00403	EF-hand calcium binding domain 2
488	8076612	EFCAB6	-3.7957346	-1.1597	-1.13537	0.0012209	EF-hand calcium binding domain 6
489	8120222	EFHC1	-3.0211825	-2.3901	-0.81804	0.007022	EF-hand domain containing 1
490	8049187	EFHD1	-2.263568	-3.5374	-1.7407	0.0354945	EF-hand domain family member D1
491	7933872	EGR2	5.5365258	1.4308	2.164128	0.0000243	early growth response 2
492	7941311	EHBP1L1//	2.3942052	-3.3483	0.532997	0.0271255	EH domain binding protein 1 like 1///signal-induced proliferation-as
493	8041225	EHD3	2.4277749	-3.299	0.795064	0.0252933	EH domain containing 3
494	8078962	EIF1B	2.196745	-3.6322	0.488936	0.0406417	eukaryotic translation initiation factor 1B
495	7988283	EIF3J-AS1	-2.4200292	-3.3104	-0.67256	0.0257055	EIF3J antisense RNA 1 (head to head)
496	8021222	ELAC1	2.5159412	-3.1681	0.682298	0.0210173	elaC ribonuclease Z 1
497	7947481	ELF5	3.1001204	-2.2656	3.861275	0.0058915	E74 like ETS transcription factor 5
498	8131096	ELFN1	2.1213427	-3.7375	0.415791	0.047264	extracellular leucine rich repeat and fibronectin type III domain conta
499	8180411	ELMO1	2.4794012	-3.2225	1.016925	0.0226999	engulfment and cell motility 1
500	7996468	ELMO3	-3.6534623	-1.3857	-1.75664	0.0016889	engulfment and cell motility 3
501	7943562	ELMOD1	-2.3897715	-3.3548	-0.39817	0.0273766	ELMO domain containing 1
502	8097529	ELMOD2	-2.8674333	-2.6308	-0.73847	0.009856	ELMO domain containing 2
503	8123920	ELOVL2	-2.2256901	-3.5913	-2.25144	0.0383334	ELOVL fatty acid elongase 2
504	8086784	ELP6	-2.3836466	-3.3637	-0.67512	0.0277269	elongator acetyltransferase complex subunit 6
505	7982507	EMC4///EM	-2.548493	-3.1193	-0.55235	0.0196178	ER membrane protein complex subunit 4///ER membrane protein co
506	8041592	EML4///AL	2.3894751	-3.3552	0.592455	0.0273934	echinoderm microtubule associated protein like 4///anaplastic lymph
507	7999387	EMP2	-2.7317426	-2.8407	-1.18087	0.0132456	epithelial membrane protein 2
508	8010295	ENGASE	2.5135842	-3.1716	0.43459	0.0211222	endo-beta-N-acetylglucosaminidase
509	8122099	ENPP1	-3.5780532	-1.5057	-1.85046	0.0020051	ectonucleotide pyrophosphatase/phosphodiesterase 1

510	8126750	ENPP5	-2.4963957	-3.1972	-1.33375	0.0219022	ectonucleotide pyrophosphatase/phosphodiesterase 5 (putative)
511	7899534	EPB41	2.2264655	-3.5902	0.542129	0.0382733	erythrocyte membrane protein band 4.1
512	8044882	EPB41L5	-4.0607923	-0.7418	-0.92471	0.0006664	erythrocyte membrane protein band 4.1 like 5
513	8128284	EPHA7	4.0472705	-0.763	2.350459	0.0006873	EPH receptor A7
514	8082846	EPHB1	3.227523	-2.0638	1.322291	0.0044302	EPH receptor B1
515	8136811	EPHB6	2.7597675	-2.7976	0.972676	0.0124649	EPH receptor B6
516	7956120	ERBB3	-2.1221146	-3.7364	-0.81951	0.0471915	erb-b2 receptor tyrosine kinase 3
517	8058627	ERBB4	-2.5684917	-3.0892	-1.91031	0.0188021	erb-b2 receptor tyrosine kinase 4
518	8000013	ERI2	-3.1994151	-2.1084	-0.94816	0.0047186	ERI1 exoribonuclease family member 2
519	8148985	ERICH1	2.5173156	-3.166	0.683951	0.0209564	glutamate rich 1
520	8123467	ERMARD	-3.2091515	-2.093	-0.72425	0.0046167	ER membrane associated RNA degradation
521	7976642	ERVH-4	-2.2619886	-3.5397	-0.73165	0.0356089	endogenous retrovirus group H member 4
522	8122843	ESR1	-2.6566446	-2.9556	-3.41477	0.0155737	estrogen receptor 1
523	7954701	ETFBKMT	-2.2592239	-3.5436	-0.89812	0.0358099	electron transfer flavoprotein beta subunit lysine methyltransferase
524	7952601	ETS1	2.2492046	-3.5579	0.885207	0.0365474	ETS proto-oncogene 1, transcription factor
525	8092578	ETV5	2.2339689	-3.5796	0.965137	0.0376957	ETS variant 5
526	7984511	EWSAT1	-2.2379653	-3.5739	-3.11471	0.0373913	Ewing sarcoma associated transcript 1
527	7912385	EXOSC10	-2.6631457	-2.9457	-0.35429	0.0153577	exosome component 10
528	8127396	EYS	-2.2464718	-3.5618	-1.05215	0.036751	eyes shut homolog (Drosophila)
529	7922174	F5	2.0944224	-3.7746	1.682887	0.0498564	coagulation factor V
530	8167930	FAAH2	-3.0085693	-2.41	-0.78263	0.0072211	fatty acid amide hydrolase 2
531	8018251	FADS6	2.905043	-2.5722	0.804834	0.009075	fatty acid desaturase 6
532	7985268	FAH	-2.4404915	-3.2802	-0.95559	0.0246299	fumarylacetoacetate hydrolase
533	8043564	FAHD2A	2.2057458	-3.6195	0.577982	0.0399107	fumarylacetoacetate hydrolase domain containing 2A
534	8018097	FAM104A	-2.1521437	-3.6947	-0.37747	0.0444484	family with sequence similarity 104 member A
535	7940153	FAM111A	-2.9467974	-2.5069	-1.01001	0.0082777	family with sequence similarity 111 member A
536	8162466	FAM120AC	-3.4843425	-1.655	-0.76475	0.0024808	family with sequence similarity 120A opposite strand
537	8026787	FAM129C	2.297327	-3.489	1.439284	0.0331288	family with sequence similarity 129 member C
538	8120552	FAM135A	2.5104119	-3.1763	0.886355	0.0212642	family with sequence similarity 135 member A
539	7932243	FAM171A1	3.1505149	-2.1859	1.482906	0.0052646	family with sequence similarity 171 member A1
540	7973918	FAM177A1	-2.7577384	-2.8007	-0.75708	0.01252	family with sequence similarity 177 member A1
541	7974125	FAM179B	-2.2522297	-3.5536	-0.69798	0.0363233	family with sequence similarity 179 member B
542	8139160	FAM183BP	-2.6070545	-3.031	-0.73549	0.0173188	acyloxyacyl hydrolase (neutrophil)
543	8088219	FAM208A	-2.2038016	-3.6223	-0.47131	0.0400675	family with sequence similarity 208 member A
544	8017867	FAM20A	2.8721991	-2.6234	1.461276	0.0097535	family with sequence similarity 20 member A
545	7897119	FAM213B	-2.1562338	-3.689	-0.65772	0.0440863	family with sequence similarity 213 member B
546	7988970	FAM214A	-3.0125052	-2.4038	-0.93808	0.0071584	family with sequence similarity 214 member A
547	8131881	FAM221A	-2.9543563	-2.4951	-1.10209	0.0081408	family with sequence similarity 221 member A
548	8161154	FAM221B	-2.1907495	-3.6406	-0.67006	0.0411355	family with sequence similarity 221 member B
549	7988625	FAM227B	-2.9271929	-2.5376	-1.16289	0.0086433	family with sequence similarity 227 member B
550	8074734	FAM230B	-2.2840324	-3.5081	-0.70868	0.0340425	family with sequence similarity 230 member B (non-protein coding)
551	8005739	FAM27E5	2.5801889	-3.0716	0.47933	0.01834	family with sequence similarity E5
552	7977445	FAM30A	2.5101867	-3.1767	1.097236	0.0212743	family with sequence similarity 30, member A
553	7928909	FAM35A	-2.2004169	-3.627	-0.48112	0.040342	family with sequence similarity 35 member A
554	8084891	FAM43A	2.0977445	-3.77	0.552145	0.0495296	family with sequence similarity 43 member A
555	8127778	FAM46A	2.1974438	-3.6312	0.933487	0.0405845	family with sequence similarity 46 member A
556	7914015	FAM46B	-2.3854892	-3.3611	-1.00787	0.0276211	family with sequence similarity 46 member B
557	8095819	FAM47E-ST	-3.3033191	-1.9433	-2.80977	0.0037358	FAM47E-STBD1 readthrough///family with sequence similarity 47 me
558	8095826	FAM47E-ST	-2.7733139	-2.7767	-0.92549	0.0121036	FAM47E-STBD1 readthrough///starch binding domain 1
559	8050427	FAM49A	2.7574492	-2.8012	1.1221	0.0125278	family with sequence similarity 49 member A
560	8116658	FAM50B	-2.9973807	-2.4275	-0.64713	0.0074023	family with sequence similarity 50 member B
561	7919872	FAM63A	-2.2637394	-3.5372	-0.87331	0.0354821	family with sequence similarity 63 member A
562	7983940	FAM63B	-2.8541094	-2.6515	-0.77032	0.0101478	family with sequence similarity 63 member B
563	8124280	FAM65B	3.2836935	-1.9745	2.00247	0.0039046	family with sequence similarity 65 member B
564	7960881	FAM90A1	2.5795693	-3.0725	0.383266	0.0183642	family with sequence similarity 90 member A1
565	7937707	FAM99A	2.3500081	-3.4128	0.784532	0.0297266	family with sequence similarity 99 member A (non-protein coding)
566	8033487	FBN3	2.7178247	-2.8621	0.793523	0.0136504	fibrillin 3
567	7996423	FBXL8	-2.6339043	-2.9902	-0.5768	0.0163522	F-box and leucine rich repeat protein 8
568	8023843	FBXO15	-2.6781369	-2.9229	-1.63675	0.0148704	F-box protein 15
569	8036676	FBXO17	2.2969236	-3.4896	0.812279	0.0331561	F-box protein 17
570	7966760	FBXO21	-2.5376114	-3.1356	-0.45141	0.0200756	F-box protein 21
571	7985053	FBXO22	-2.9337975	-2.5272	-0.4523	0.0085184	F-box protein 22
572	7912750	FBXO42	-2.1772029	-3.6597	-0.37753	0.0422714	F-box protein 42
573	8103755	FBXO8	-2.3388596	-3.429	-0.55476	0.0304183	F-box protein 8
574	8120251	FBXO9	-2.7928613	-2.7465	-0.47055	0.0115997	F-box protein 9
575	8103206	FBXW7	2.1497674	-3.698	0.610901	0.0446601	F-box and WD repeat domain containing 7
576	8033420	FCER2	2.9165308	-2.5542	2.096722	0.0088485	Fc fragment of IgE receptor II
577	8026830	FCHO1	2.3178764	-3.4594	0.816884	0.0317609	FCH domain only 1
578	7923917	FCMR	3.1400914	-2.2024	2.05797	0.0053887	Fc fragment of IgM receptor
579	7921319	FCRL1	2.3024996	-3.4816	2.076446	0.0327794	Fc receptor like 1
580	7921298	FCRL2	3.1304302	-2.2177	1.753422	0.0055063	Fc receptor like 2
581	7921275	FCRL3	2.652541	-2.9619	1.693595	0.0157115	Fc receptor like 3
582	7921237	FCRL5	3.0158086	-2.3986	1.666464	0.0071061	Fc receptor like 5

583	7906475	FCRL6	2.7121606	-2.8708	0.594871	0.0138184	Fc receptor like 6
584	7906786	FCRLA	2.6877288	-2.9082	1.405106	0.0145664	Fc receptor like A
585	8095467	FDCSP	3.6117199	-1.4521	4.028388	0.0018573	follicular dendritic cell secreted protein
586	7951654	FDXACB1	-2.4468646	-3.2708	-0.66918	0.0243036	ferredoxin-fold anticodon binding domain containing 1
587	8024898	FEM1A	-2.1760867	-3.6612	-0.33505	0.0423663	fem-1 homolog A
588	7984470	FEM1B	-2.1652221	-3.6764	-0.3949	0.0432998	fem-1 homolog B
589	8113616	FEM1C	-2.2113955	-3.6115	-0.42644	0.0394579	fem-1 homolog C
590	8065948	FER1L4	3.3585184	-1.8555	1.550651	0.0032984	fer-1 like family member 4, pseudogene
591	8065920	FER1L4	3.0643805	-2.3221	1.086961	0.0063796	fer-1 like family member 4, pseudogene
592	7954729	FGD4	2.9950625	-2.4312	0.594132	0.0074403	FYVE, RhoGEF and PH domain containing 4
593	7950036	FGF3	2.1824367	-3.6523	0.502877	0.0418292	fibroblast growth factor 3
594	7967993	FGF9	2.9123953	-2.5607	2.111544	0.0089294	fibroblast growth factor 9
595	8123342	FGFR1OP	-2.310378	-3.4702	-0.68658	0.0322539	FGFR1 oncogene partner
596	7914112	FGR	2.1176465	-3.7426	0.731168	0.0476125	FGR proto-oncogene, Src family tyrosine kinase
597	8029847	FKRP	2.3379372	-3.4303	0.458215	0.0304762	fukutin related protein
598	8030578	FLJ26850	-2.5056848	-3.1834	-0.51563	0.0214773	FLJ26850 protein
599	8073939	FLJ44385	-2.1252614	-3.732	-0.67423	0.046897	uncharacterized FLJ44385
600	7952673	FLJ45950	-2.6512906	-2.9638	-2.06157	0.0157537	FLJ45950 protein
601	7940851	FLRT1	2.1204972	-3.7386	0.325243	0.0473435	fibronectin leucine rich transmembrane protein 1
602	7987145	FMN1	-2.6708246	-2.934	-0.80022	0.0151062	formin 1
603	8007757	FMNL1	3.3846366	-1.8139	1.123759	0.0031093	formin like 1
604	8045736	FMNL2	2.6961866	-2.8953	1.058435	0.0143032	formin like 2
605	8113914	FNIP1	-2.9438151	-2.5116	-0.54094	0.0083324	folliculin interacting protein 1
606	8146268	FNTA	2.520989	-3.1605	0.618471	0.0207943	farnesyltransferase, CAAX box, alpha
607	7999718	FOPNL	-3.1749229	-2.1472	-1.0467	0.0049848	FGFR1OP N-terminal like
608	7978706	FOXA1	-3.2083697	-2.0942	-2.63738	0.0046248	forkhead box A1
609	7931348	FOXI2	2.6878561	-2.908	0.576953	0.0145624	forkhead box I2
610	8121365	FOXO3	2.2903725	-3.499	0.546327	0.0336039	forkhead box O3
611	8160168	FREM1	2.7410323	-2.8264	2.022785	0.0129818	FRAS1 related extracellular matrix 1
612	8061497	FRG1BP	-2.6295307	-2.9969	-1.01121	0.016506	FSHD region gene 1 family member B, pseudogene
613	7987426	FSIP1	-2.637637	-2.9846	-2.84409	0.0162219	fibrous sheath interacting protein 1
614	8023995	FSTL3	-3.3870554	-1.81	-0.98124	0.0030924	follistatin like 3
615	7948656	FTH1	-2.2389633	-3.5725	-0.33557	0.0373157	ferritin heavy chain 1
616	8170360	FTH1P5///I	-2.3890771	-3.3558	-0.36917	0.0274161	ferritin heavy chain 1 pseudogene 5///ferritin heavy chain 1 pseudog
617	8158686	FUBP3	-2.4817152	-3.2191	-0.50749	0.0225897	far upstream element binding protein 3
618	7937208	FUOM	2.4182967	-3.3129	0.481072	0.0257986	fucose mutarotase
619	8165398	FUT7	2.6973266	-2.8935	0.58562	0.0142681	fucosyltransferase 7
620	8027748	FXYD3	-3.5787016	-1.5047	-2.00152	0.0020022	FXYD domain containing ion transport regulator 3
621	8128956	FYN	2.2808494	-3.5127	0.852526	0.0342646	FYN proto-oncogene, Src family tyrosine kinase
622	7959850	FZD10	2.2060907	-3.619	0.426431	0.0398829	frizzled class receptor 10
623	8047487	FZD7	3.4102981	-1.773	1.311747	0.002934	frizzled class receptor 7
624	8007561	G6PC3	-2.1021069	-3.764	-0.68071	0.0491035	glucose-6-phosphatase catalytic subunit 3
625	8074212	GAB4	2.5385209	-3.1342	0.398786	0.0200369	GRB2 associated binding protein family member 4
626	8109677	GABRG2	-2.0989059	-3.7684	-0.56454	0.0494159	gamma-aminobutyric acid type A receptor gamma2 subunit
627	8109407	GALNT10	-2.433505	-3.2905	-0.75174	0.0249923	polypeptide N-acetylgalactosaminyltransferase 10
628	8098328	GALNT7	-2.6942382	-2.8982	-1.23365	0.0143634	polypeptide N-acetylgalactosaminyltransferase 7
629	8154916	GALT	2.106334	-3.7582	0.544894	0.0486938	galactose-1-phosphate uridylyltransferase
630	8132929	GBAS	-2.1103124	-3.7527	-0.47773	0.048311	glioblastoma amplified sequence
631	8088958	GBE1	-2.4657004	-3.2429	-0.71059	0.0233627	glucan (1,4-alpha-), branching enzyme 1
632	8040479	GDF7	2.1127845	-3.7493	0.347173	0.0480745	growth differentiation factor 7
633	8000799	GDPD3	-2.4945696	-3.2	-2.79235	0.0219866	glycerophosphodiester phosphodiesterase domain containing 3
634	8149629	GFRA2	4.372468	-0.2583	1.289372	0.0003273	GDNF family receptor alpha 2
635	8114415	GFRA3	4.0526348	-0.7546	4.131013	0.0006789	GDNF family receptor alpha 3
636	7928752	GHITM	-2.4912889	-3.2048	-0.71153	0.0221391	growth hormone inducible transmembrane protein
637	8085293	GHRL	-2.2879572	-3.5025	-0.6165	0.0337703	ghrelin/obestatin prepropeptide
638	8141371	GJC3	-2.1059419	-3.7587	-1.27124	0.0487317	gap junction protein gamma 3
639	7927028	GJD4	2.3331788	-3.4372	0.445443	0.0307765	gap junction protein delta 4
640	8101256	GK2	-2.2788735	-3.5155	-1.01881	0.0344032	glycerol kinase 2
641	8086028	GLB1	2.1109527	-3.7518	0.58217	0.0482497	galactosidase beta 1
642	7957253	GLIPR1L2	-2.438176	-3.2836	-1.02843	0.0247495	GLI pathogenesis-related 1 like 2
643	8155192	GLIPR2	2.5751629	-3.0791	0.973374	0.0185372	GLI pathogenesis related 2
644	7916304	GLIS1	2.3510499	-3.4113	0.664376	0.0296627	GLIS family zinc finger 1
645	8174263	GLRA4	2.2641714	-3.5365	0.592425	0.0354508	glycine receptor alpha 4
646	7966259	GLTP	-2.6632331	-2.9456	-0.47246	0.0153548	glycolipid transfer protein
647	8003948	GLTPD2	2.1619295	-3.681	0.379458	0.0435864	glycolipid transfer protein domain containing 2
648	8169711	GLUD2	-2.2125851	-3.6099	-0.56782	0.0393632	glutamate dehydrogenase 2
649	8154951	GLUL	-2.6681615	-2.9381	-0.70245	0.015193	glutamate-ammonia ligase
650	7922689	GLUL	-2.6019943	-3.0386	-0.742	0.0175069	glutamate-ammonia ligase
651	7979260	GMFB	-2.6169525	-3.016	-0.57891	0.0169563	glia maturation factor beta
652	8024557	GNA11	-3.2562131	-2.0182	-0.5989	0.0041537	G protein subunit alpha 11
653	7916843	GNG12	-2.5261176	-3.1528	-0.8243	0.02057	G protein subunit gamma 12
654	7925250	GNG4	2.1715465	-3.6676	1.496529	0.0427541	G protein subunit gamma 4
655	8037785	GNG8	2.1436573	-3.7065	0.464996	0.0452085	G protein subunit gamma 8

656	7965746	GOLGA2P5	-2.9326479	-2.529	-0.88602	0.00854	golgin A2 pseudogene 5
657	8078569	GOLGA4	2.1731511	-3.6653	0.478446	0.0426167	golgin A4
658	7929711	GOLGA7B	2.5395152	-3.1328	0.423686	0.0199948	golgin A7 family member B
659	8089930	GOLGB1	-2.4230693	-3.3059	-0.47442	0.025543	golgin B1
660	8162117	GOLM1	-2.5349214	-3.1396	-1.01659	0.0201903	golgi membrane protein 1
661	7923628	GOLT1A	-2.8593196	-2.6434	-0.90679	0.0100327	golgi transport 1A
662	8004024	GP1BA	3.3877609	-1.8089	0.837937	0.0030875	glycoprotein Ib platelet alpha subunit
663	8096116	GPAT3	3.1716054	-2.1525	1.149704	0.005022	glycerol-3-phosphate acyltransferase 3
664	8141466	GPC2	2.1614738	-3.6817	0.454126	0.0436262	glypican 2
665	8078386	GPD1L	-2.5301929	-3.1467	-1.0059	0.0203934	glycerol-3-phosphate dehydrogenase 1-like
666	7966321	GPN3	-3.3989285	-1.7911	-0.85917	0.0030105	GPN-loop GTPase 3
667	8083839	GPR160	-3.6623046	-1.3716	-1.69345	0.0016552	G protein-coupled receptor 160
668	8045075	GPR17	2.2442269	-3.565	0.462382	0.036919	G protein-coupled receptor 17
669	8091503	GPR171	2.6273498	-3.0002	1.089535	0.0165833	G protein-coupled receptor 171
670	7972548	GPR18	3.1631349	-2.1659	1.391451	0.0051181	G protein-coupled receptor 18
671	7972557	GPR183	2.727913	-2.8466	1.1821	0.0133558	G protein-coupled receptor 183
672	8045336	GPR39	-3.1736394	-2.1493	-5.26091	0.0049992	G protein-coupled receptor 39
673	8121454	GPR6	2.1285787	-3.7274	0.444991	0.0465883	G protein-coupled receptor 6
674	7903694	GPR61	2.4463937	-3.2715	0.554507	0.0243276	G protein-coupled receptor 61
675	7954065	GPRC5A	-2.474435	-3.2299	-1.57422	0.0229381	G protein-coupled receptor class C group 5 member A
676	7999909	GPRC5B	3.0252861	-2.3837	1.314557	0.0069583	G protein-coupled receptor class C group 5 member B
677	8009639	GPRC5C	-3.5420865	-1.563	-3.65416	0.002176	G protein-coupled receptor class C group 5 member C
678	8125373	GPSM3	2.2734113	-3.5233	0.707632	0.0347891	G-protein signaling modulator 3
679	8179987	GPSM3	2.2734113	-3.5233	0.707632	0.0347891	G-protein signaling modulator 3
680	8178790	GPSM3	2.1448676	-3.7048	0.672477	0.0450994	G-protein signaling modulator 3
681	7901460	GPX7	2.1370098	-3.7158	1.249519	0.0458122	glutathione peroxidase 7
682	7955578	GRASP	2.2789305	-3.5154	0.4433	0.0343992	general receptor for phosphoinositides 1 associated scaffold protein
683	8040292	GREB1	-2.2430916	-3.5666	-2.41632	0.0370042	growth regulation by estrogen in breast cancer 1
684	8020384	GREB1L	-2.7502108	-2.8123	-2.14826	0.0127261	growth regulation by estrogen in breast cancer 1 like
685	8040190	GRHL1	2.1966244	-3.6324	1.272199	0.0406516	grainyhead like transcription factor 1
686	7914974	GRIK3	-2.134794	-3.7188	-1.48494	0.046015	glutamate ionotropic receptor kainate type subunit 3
687	7950921	GRM5	2.1034996	-3.7621	0.410635	0.0489682	glutamate metabotropic receptor 5
688	7976571	GSKIP	-2.5858206	-3.0631	-0.74388	0.0181213	GSK3B interacting protein
689	8096688	GSTCD	-2.6657582	-2.9417	-0.67545	0.0152717	glutathione S-transferase C-terminal domain containing
690	8128890	GSTM2P1	2.3868358	-3.3591	0.657902	0.027544	glutathione S-transferase mu 2 pseudogene 1
691	7918379	GSTM3	-4.1670133	-0.5759	-2.51129	0.0005229	glutathione S-transferase mu 3
692	7903742	GSTM4	-2.7996977	-2.7359	-0.91616	0.0114283	glutathione S-transferase mu 4
693	7930311	GSTO2	-3.0245181	-2.3849	-1.26609	0.0069702	glutathione S-transferase omega 2
694	7989315	GTF2A2	-2.3245343	-3.4497	-0.48195	0.0313291	general transcription factor IIA subunit 2
695	8081953	GTF2E1	-2.5447695	-3.1249	-0.6694	0.0197734	general transcription factor IIE subunit 1
696	7963817	GTSF1	2.2816811	-3.5115	1.956088	0.0342065	gametocyte specific factor 1
697	7946275	GVINP1	2.5749039	-3.0795	1.263811	0.0185474	GTPase, very large interferon inducible pseudogene 1
698	8023977	GZMM	2.485538	-3.2134	0.444249	0.0224087	granzyme M
699	8170994	H2AFB1///	2.4876126	-3.2103	0.496369	0.0223111	H2A histone family member B1///H2A histone family member B2///H
700	8176249	H2AFB1///	2.4876126	-3.2103	0.496369	0.0223111	H2A histone family member B1///H2A histone family member B2///H
701	8139421	H2AFV	-2.5858702	-3.063	-0.547	0.0181194	H2A histone family member V
702	8051799	HAAO	2.2356804	-3.5771	1.093582	0.0375651	3-hydroxyanthranilate 3,4-dioxygenase
703	8090091	HACD2///H	-2.5587319	-3.1039	-0.62759	0.0191961	3-hydroxyacyl-CoA dehydratase 2///3-hydroxyacyl-CoA dehydratase
704	8103745	HAND2	2.463815	-3.2457	0.465462	0.0234553	heart and neural crest derivatives expressed 2
705	7946061	HBE1	-2.2941637	-3.4935	-1.09772	0.0333441	hemoglobin subunit epsilon 1
706	8114572	HBEGF	2.1128733	-3.7492	0.426363	0.0480661	heparin binding EGF like growth factor
707	8129706	HBS1L	-2.2366155	-3.5758	-0.68549	0.0374939	HBS1 like translational GTPase
708	8117809	HCG9	-2.1396027	-3.7122	-0.5372	0.0455759	HLA complex group 9 (non-protein coding)
709	8177740	HCG9	-2.1396027	-3.7122	-0.5372	0.0455759	HLA complex group 9 (non-protein coding)
710	7899643	HCRTR1	2.3220629	-3.4533	0.550573	0.0314888	hypocretin receptor 1
711	7944911	HEPN1	-2.3219404	-3.4535	-0.74342	0.0314967	hepatocellular carcinoma, down-regulated 1
712	8007745	HEXIM1	-3.8381318	-1.0926	-1.02225	0.0011083	hexamethylene bisacetamide inducible 1
713	8089851	HGD	-2.3087784	-3.4725	-2.90306	0.0323599	homogentisate 1,2-dioxygenase
714	8093278	HGD	-2.3087784	-3.4725	-2.90306	0.0323599	homogentisate 1,2-dioxygenase
715	7909510	HHAT	-2.260664	-3.5416	-0.89552	0.0357051	hedgehog acyltransferase
716	8161147	HINT2	-2.1923141	-3.6384	-0.65556	0.0410061	histidine triad nucleotide binding protein 2
717	8121886	HINT3	-2.2284705	-3.5874	-0.49189	0.0381181	histidine triad nucleotide binding protein 3
718	7959500	HIP1R	2.2909802	-3.4981	0.617983	0.0335621	huntingtin interacting protein 1 related
719	8000748	HIRIP3	-2.461578	-3.249	-0.78558	0.0235657	HIRA interacting protein 3
720	8117334	HIST1H4A	-2.1517839	-3.6952	-1.60788	0.0444804	histone cluster 1, H4a
721	7919584	HIST2H2BF	-3.3499534	-1.8691	-1.23523	0.0033628	histone cluster 2, H2bf///histone cluster 2, H2ba (pseudogene)
722	7919606	HIST2H2BF	-3.7084192	-1.2983	-1.29188	0.00149	histone cluster 2, H2bf///histone cluster 2, H2ba (pseudogene)///hist
723	8180093	HLA-DOA	2.8106911	-2.7189	0.861158	0.0111577	major histocompatibility complex, class II, DO alpha
724	8125545	HLA-DOA	2.1370224	-3.7157	0.883404	0.045811	major histocompatibility complex, class II, DO alpha
725	8125470	HLA-DOB	2.3928041	-3.3503	1.1888	0.0272046	major histocompatibility complex, class II, DO beta
726	8178833	HLA-DOB	2.3928041	-3.3503	1.1888	0.0272046	major histocompatibility complex, class II, DO beta
727	7909890	HLX	2.5528398	-3.1127	0.465953	0.0194378	H2.0 like homeobox
728	7931181	HMX3	2.7979258	-2.7387	0.538724	0.0114725	H6 family homeobox 3

729	7959234	HNF1A	2.5473147	-3.121	0.472882	0.0196669	HNF1 homeobox A
730	8110450	HNRNPAB	-2.1694006	-3.6706	-0.56416	0.0429386	heterogeneous nuclear ribonucleoprotein A/B
731	8047059	HNRNPCL1	-2.4242418	-3.3042	-0.41018	0.0254806	heterogeneous nuclear ribonucleoprotein C-like 1///heterogeneous n
732	8162047	HNRNPK	-2.2223587	-3.596	-0.32292	0.0385928	heterogeneous nuclear ribonucleoprotein K
733	7901765	HOOK1	-2.3686614	-3.3856	-0.89697	0.0286018	hook microtubule tethering protein 1
734	8034521	HOOK2	-2.4543329	-3.2597	-0.55146	0.0239264	hook microtubule tethering protein 2
735	8146243	HOOK3	2.4859393	-3.2128	0.578836	0.0223898	hook microtubule tethering protein 3
736	7919787	HORMAD1	2.7081651	-2.8769	2.459273	0.0139382	HORMA domain containing 1
737	8016438	HOXB2	-3.5051281	-1.6219	-1.58772	0.0023665	homeobox B2
738	8016444	HOXB3	-5.7548945	1.7227	-1.4743	0.0000151	homeobox B3
739	8016452	HOXB4	-2.2537836	-3.5514	-0.51179	0.0362087	homeobox B4
740	8016457	HOXB5	-2.3162042	-3.4618	-1.4023	0.0318703	homeobox B5
741	8016463	HOXB6	-2.6858803	-2.911	-1.78962	0.0146245	homeobox B6
742	7955855	HOXC11	-2.5320121	-3.144	-1.81193	0.020315	homeobox C11
743	7955887	HOXC5	-2.9295429	-2.5339	-1.21201	0.0085986	homeobox C5
744	7955873	HOXC6///H	-2.8156655	-2.7112	-1.24719	0.0110373	homeobox C6///homeobox C4
745	8101780	HPGDS	-2.1214761	-3.7373	-1.04166	0.0472514	hematopoietic prostaglandin D synthase
746	7929988	HPS6	-2.4092961	-3.3261	-0.59738	0.0262871	HPS6, biogenesis of lysosomal organelles complex 2 subunit 3
747	8067394	HRH3	2.0948631	-3.774	0.610223	0.0498129	histamine receptor H3
748	7969665	HS6ST3	-2.7544241	-2.8058	-1.64422	0.0126103	heparan sulfate 6-O-sulfotransferase 3
749	8072143	HSCB	-2.2076736	-3.6168	-0.4816	0.0397556	HscB mitochondrial iron-sulfur cluster cochaperone
750	8047228	HSPE1-MC	-2.5837721	-3.0662	-0.63543	0.0182005	HSPE1-MOB4 readthrough///MOB family member 4, phocein
751	8120983	HTR1E	-3.2125807	-2.0875	-1.50062	0.0045813	5-hydroxytryptamine receptor 1E
752	8068180	HUNK	2.3195432	-3.457	0.604855	0.0316523	hormonally up-regulated Neu-associated kinase
753	7985192	HYKK	-2.6483536	-2.9683	-0.73927	0.0158533	hydroxylysine kinase
754	8138202	ICA1	-2.733631	-2.8378	-0.7691	0.0131915	islet cell autoantigen 1
755	7989347	ICE2	-2.9208691	-2.5475	-0.70186	0.0087644	interactor of little elongation complex ELL subunit 2
756	8070720	ICOSLG	2.77871	-2.7684	1.008337	0.0119624	inducible T-cell costimulator ligand
757	7931748	IDI2	3.1546087	-2.1794	1.016915	0.0052166	isopentenyl-diphosphate delta isomerase 2
758	8023175	IER3IP1	-2.0980306	-3.7696	-0.61546	0.0495016	immediate early response 3 interacting protein 1
759	8164554	IER5L	2.153129	-3.6933	0.33705	0.0443609	immediate early response 5 like
760	8068238	IFNAR2	2.8866304	-2.6009	0.714856	0.0094496	interferon alpha and beta receptor subunit 2
761	8160435	IFNE	-2.6823106	-2.9165	-2.91851	0.0147374	interferon epsilon
762	7998542	IFT140///IF	-2.1066886	-3.7577	-0.67351	0.0486596	intraflagellar transport 140///intraflagellar transport 140
763	7952069	IFT46	-2.5794472	-3.0727	-0.73727	0.0183689	intraflagellar transport 46
764	8091737	IFT80	-2.5769496	-3.0765	-0.64433	0.0184669	intraflagellar transport 80
765	7958620	IFT81	-2.9796603	-2.4554	-0.84898	0.0076982	intraflagellar transport 81
766	7967933	IFT88	-3.1600886	-2.1708	-0.84152	0.0051531	intraflagellar transport 88
767	7973900	IGBP1P1	-2.1959587	-3.6333	-0.79032	0.0407062	immunoglobulin (CD79A) binding protein 1 pseudogene 1
768	7986359	IGF1R	-2.6982433	-2.8921	-1.49585	0.0142399	insulin like growth factor 1 receptor
769	7986383	IGF1R	-2.53229	-3.1436	-1.34899	0.0203031	insulin like growth factor 1 receptor
770	8036136	IGFLR1	2.2705501	-3.5274	0.5312	0.0349928	IGF like family receptor 1
771	7981720	IGHA1///S	2.1072877	-3.7569	1.639721	0.0486018	immunoglobulin heavy constant alpha 1///single-chain Fv fragment/
772	7981601	IGHM///IG	2.8491178	-2.6593	1.982582	0.0102593	immunoglobulin heavy constant mu///immunoglobulin heavy consta
773	7981728	IGHM///IG	2.2038347	-3.6222	1.807653	0.0400649	immunoglobulin heavy constant mu///immunoglobulin heavy variab
774	8001104	IGHV3-48/	2.1244429	-3.7332	1.610462	0.0469734	immunoglobulin heavy variable 3-48///immunoglobulin heavy variab
775	8043449	IGK///IGKC	2.8370841	-2.678	2.013162	0.0105329	immunoglobulin kappa locus///immunoglobulin kappa constant
776	8043360	IGK///IGKC	2.7815061	-2.7641	2.043072	0.0118899	immunoglobulin kappa locus///immunoglobulin kappa constant
777	8043438	IGKC	3.5157713	-1.6049	1.759678	0.00231	immunoglobulin kappa constant
778	8053713	IGKC	3.515689	-1.605	1.790361	0.0023104	immunoglobulin kappa constant
779	8043433	IGKC	3.4858407	-1.6526	1.756279	0.0024724	immunoglobulin kappa constant
780	8043474	IGKC	2.7170637	-2.8633	1.722549	0.0136728	immunoglobulin kappa constant
781	8043465	IGKC	2.5359947	-3.138	1.98651	0.0201445	immunoglobulin kappa constant
782	8043459	IGKC	2.3755434	-3.3756	1.962586	0.0281968	immunoglobulin kappa constant
783	8043484	IGKC	2.129976	-3.7255	1.671009	0.0464589	immunoglobulin kappa constant
784	8053735	IGKC	2.129976	-3.7255	1.671009	0.0464589	immunoglobulin kappa constant
785	8161563	IGKC	2.129976	-3.7255	1.671009	0.0464589	immunoglobulin kappa constant
786	8043480	IGKC	2.1040594	-3.7613	1.663472	0.0489139	immunoglobulin kappa constant
787	8074909	IGLL1	2.2461436	-3.5623	0.424602	0.0367755	immunoglobulin lambda like polypeptide 1
788	8132819	IKZF1	2.1399624	-3.7117	1.036939	0.0455432	IKAROS family zinc finger 1
789	8014891	IKZF3	2.7852297	-2.7583	1.633519	0.011794	IKAROS family zinc finger 3
790	7944152	IL10RA	2.2151043	-3.6063	1.050642	0.0391633	interleukin 10 receptor subunit alpha
791	8068254	IL10RB///IL	3.01188	-2.4048	0.533583	0.0071683	interleukin 10 receptor subunit beta///interleukin 10 receptor subuni
792	7985364	IL16	2.9705982	-2.4696	1.534915	0.007854	interleukin 16
793	8080562	IL17RB	-2.5236246	-3.1566	-1.27143	0.0206787	interleukin 17 receptor B
794	8044035	IL18R1///IL	2.2821772	-3.5108	0.920817	0.0341718	interleukin 18 receptor 1///interleukin 18 receptor 1
795	8044049	IL18RAP	2.325574	-3.4482	0.855668	0.0312622	interleukin 18 receptor accessory protein
796	7909261	IL20	-2.5145124	-3.1702	-1.95504	0.0210808	interleukin 20
797	7902189	IL23R	-3.5617416	-1.5317	-1.29693	0.0020809	interleukin 23 receptor
798	8000567	IL27	-2.4195351	-3.3111	-0.62107	0.025732	interleukin 27
799	8026272	IL27RA	2.7309789	-2.8419	1.237775	0.0132675	interleukin 27 receptor subunit alpha
800	8173444	IL2RG	2.3771884	-3.3732	1.229016	0.0281008	interleukin 2 receptor subunit gamma
801	8085062	IL5RA	2.2429065	-3.5669	0.657064	0.0370181	interleukin 5 receptor subunit alpha

802	8089970	ILDR1	-3.2620589	-2.0089	-1.01849	0.0040994	immunoglobulin like domain containing receptor 1
803	8020183	IMPA2	2.2628427	-3.5384	1.413963	0.035547	inositol monophosphatase 2
804	7956423	INHBC	2.3733914	-3.3787	0.556682	0.0283229	inhibin beta C subunit
805	8163428	INIP	-2.495309	-3.1989	-0.63074	0.0219524	INTS3 and NABP interacting protein
806	8022902	INO80C	-2.2908204	-3.4984	-0.63552	0.0335731	INO80 complex subunit C
807	8072436	INPP5J	-3.1690377	-2.1566	-1.76689	0.0050509	inositol polyphosphate-5-phosphatase J
808	8044766	INSIG2	-2.6730895	-2.9306	-0.76877	0.0150328	insulin induced gene 2
809	8144894	INTS10	2.138738	-3.7134	0.556535	0.0456545	integrator complex subunit 10
810	8170027	INTS6L	3.3294154	-1.9018	1.671102	0.0035223	integrator complex subunit 6 like
811	8093258	IQCG	-2.6016093	-3.0392	-0.81382	0.0175213	IQ motif containing G
812	7984380	IQCH	-3.4281652	-1.7445	-1.02203	0.0028177	IQ motif containing H
813	7993713	IQCK	-2.1289398	-3.7269	-0.62796	0.0465548	IQ motif containing K
814	8085412	IQSEC1	2.1821251	-3.6528	0.575201	0.0418554	IQ motif and Sec7 domain 1
815	8037723	IRF2BP1	-2.8750812	-2.6189	-0.43716	0.0096921	interferon regulatory factor 2 binding protein 1
816	8116559	IRF4	2.8710005	-2.6253	2.191224	0.0097792	interferon regulatory factor 4
817	7997712	IRF8	2.1564127	-3.6887	1.070653	0.0440705	interferon regulatory factor 8
818	8174444	IRS4	2.8372485	-2.6777	3.26561	0.0105291	insulin receptor substrate 4
819	8104314	IRX1	2.5988004	-3.0435	2.059331	0.0176267	iroquois homeobox 1
820	8001147	ISY1-RAB4	-2.3588955	-3.3999	-0.61485	0.0291856	ISY1-RAB43 readthrough///RAB43, member RAS oncogene family
821	8016044	ITGA2B	2.1167891	-3.7438	0.412053	0.0476937	integrin subunit alpha 2b
822	8046695	ITGA4	2.202183	-3.6245	0.974451	0.0401986	integrin subunit alpha 4
823	8078619	ITGA9	2.1575042	-3.6872	0.867188	0.0439743	integrin subunit alpha 9
824	7995128	ITGAX	2.9712947	-2.4685	1.083381	0.0078419	integrin subunit alpha X
825	8090162	ITGB5	-2.5235049	-3.1567	-0.86313	0.020684	integrin subunit beta 5
826	7926061	ITIH2	2.8060987	-2.726	0.727504	0.01127	inter-alpha-trypsin inhibitor heavy chain 2
827	8109507	ITK	2.452237	-3.2628	1.47541	0.0240317	IL2 inducible T-cell kinase
828	8060611	ITPA	-2.1417694	-3.7091	-0.49188	0.0453792	inosine triphosphatase
829	7993622	ITPRIPL2	-2.2577915	-3.5457	-0.65558	0.0359145	inositol 1,4,5-trisphosphate receptor interacting protein like 2
830	8038532	IZUMO2	2.2784398	-3.5161	0.597573	0.0344337	IZUMO family member 2
831	7952022	JAML	2.5438404	-3.1263	1.261285	0.0198123	junction adhesion molecule like
832	7974725	JKAMP	-2.5177884	-3.1653	-0.43191	0.0209354	JNK1/MAPK8-associated membrane protein
833	7978093	JPH4	2.2090966	-3.6148	0.451249	0.0396416	junctophilin 4
834	8153328	JRK	2.8147066	-2.7127	0.432671	0.0110604	Jrk helix-turn-helix protein
835	8035445	JUND	-2.2517659	-3.5543	-0.43799	0.0363576	JunD proto-oncogene, AP-1 transcription factor subunit
836	8015412	JUP	-2.7694379	-2.7827	-0.82711	0.0122059	junction plakoglobin
837	8117189	KAAG1	-2.483073	-3.2171	-0.84719	0.0225252	kidney associated antigen 1
838	8078227	KAT2B	2.4791491	-3.2229	0.915097	0.0227119	lysine acetyltransferase 2B
839	7987180	KATNBL1P	-2.7940342	-2.7447	-0.36701	0.0115701	katanin regulatory subunit B1 like 1 pseudogene 6///katanin regulat
840	7918457	KCNA3	2.7147841	-2.8668	1.621163	0.0137403	potassium voltage-gated channel subfamily A member 3
841	8146908	KCNB2	2.4328924	-3.2914	0.503119	0.0250244	potassium voltage-gated channel subfamily B member 2
842	8135705	KCND2	-2.7826368	-2.7623	-1.49513	0.0118607	potassium voltage-gated channel subfamily D member 2
843	8051785	KCNG3	2.1030299	-3.7627	0.545224	0.0490138	potassium voltage-gated channel modifier subfamily G member 3
844	7924071	KCNH1	-2.4800597	-3.2215	-2.35087	0.0226684	potassium voltage-gated channel subfamily H member 1
845	8126173	KCNK16	-2.2736788	-3.5229	-0.65013	0.0347701	potassium two pore domain channel subfamily K member 16
846	8126153	KCNK5	2.8268969	-2.6938	2.433394	0.01077	potassium two pore domain channel subfamily K member 5
847	8115756	KCNMB1	2.1361152	-3.717	0.887824	0.045894	potassium calcium-activated channel subfamily M regulatory beta su
848	8084002	KCNMB2	3.5556295	-1.5414	0.834789	0.00211	potassium calcium-activated channel subfamily M regulatory beta su
849	7920552	KCNN3	2.1965551	-3.6325	0.685528	0.0406573	potassium calcium-activated channel subfamily N member 3
850	8152946	KCNQ3	2.5107672	-3.1758	0.382596	0.0212482	potassium voltage-gated channel subfamily Q member 3
851	8040458	KCNS3	-3.1844545	-2.1321	-1.24913	0.0048795	potassium voltage-gated channel modifier subfamily S member 3
852	8022646	KCTD1	-2.1235522	-3.7344	-0.64763	0.0470567	potassium channel tetramerization domain containing 1
853	8004331	KCTD11///	2.8988845	-2.5818	1.376237	0.0091986	potassium channel tetramerization domain containing 11///ArfGAP v
854	8009737	KCTD2	-2.3272893	-3.4458	-0.59616	0.031152	potassium channel tetramerization domain containing 2
855	7909745	KCTD3	-3.6996393	-1.3122	-1.21458	0.0015202	potassium channel tetramerization domain containing 3
856	7967175	KDM2B	2.2898554	-3.4997	0.423922	0.0336394	lysine demethylase 2B
857	8024909	KDM4B	-2.690392	-2.9041	-0.96845	0.014483	lysine demethylase 4B
858	7994269	KDM8	2.4712769	-3.2346	0.326808	0.0230908	lysine demethylase 8
859	8120675	KHDC3L	2.2795009	-3.5146	0.62393	0.0343592	KH domain containing 3 like, subcortical maternal complex member
860	8148448	KHDRBS3	2.3750593	-3.3763	1.368558	0.0282252	KH RNA binding domain containing, signal transduction associated 3
861	8138686	KIAA0087	-2.1278066	-3.7285	-3.17671	0.04666	KIAA0087
862	7989647	KIAA0101	-2.4179668	-3.3134	-1.10861	0.0258163	KIAA0101
863	7971486	KIAA0226L	2.397906	-3.3429	1.164016	0.0269176	KIAA0226 like
864	8093961	KIAA0232	-2.21507	-3.6064	-0.60153	0.039166	KIAA0232
865	7973924	KIAA0391	-2.4939651	-3.2009	-0.54349	0.0220146	KIAA0391
866	7994308	KIAA0556	-3.1524447	-2.1829	-0.79941	0.0052419	KIAA0556
867	8113113	KIAA0825	-2.3716474	-3.3813	-0.65528	0.0284254	KIAA0825
868	7899821	KIAA1522	-2.617558	-3.015	-0.80211	0.0169344	KIAA1522
869	7907882	KIAA1614	2.1951243	-3.6345	0.384238	0.0407747	KIAA1614
870	8066231	KIAA1755	2.5522165	-3.1137	1.378226	0.0194635	KIAA1755
871	8163548	KIF12	-2.7512519	-2.8107	-1.98664	0.0126974	kinesin family member 12
872	8065089	KIF16B	-4.7685037	0.3395	-1.11113	0.0001335	kinesin family member 16B
873	7923233	KIF21B	3.2438261	-2.0379	1.216436	0.004271	kinesin family member 21B
874	8123437	KIF25	2.6525522	-2.9619	0.37478	0.0157111	kinesin family member 25

875	7965322	KITLG	-2.5706559	-3.0859	-1.33672	0.0187158	KIT ligand
876	7968556	KL	2.3330395	-3.4374	0.389444	0.0307854	klotho
877	7972003	KLF12	2.2335449	-3.5802	0.617678	0.0377281	Kruppel like factor 12
878	7974229	KLHDC2	-2.1310413	-3.724	-0.57632	0.0463604	kelch domain containing 2
879	8074106	KLHDC7B	2.2763324	-3.5191	0.411376	0.0345822	kelch domain containing 7B
880	8015456	KLHL11	-2.1806148	-3.6549	-0.48387	0.0419826	kelch like family member 11
881	7923489	KLHL12	-2.8709357	-2.6254	-0.62221	0.0097806	kelch like family member 12
882	7896779	KLHL17	2.2586045	-3.5445	0.374	0.0358551	kelch like family member 17
883	8046160	KLHL41	-2.5955542	-3.0484	-0.76589	0.0177492	kelch like family member 41
884	8092392	KLHL6	2.1835608	-3.6508	0.943945	0.0417348	kelch like family member 6
885	8038643	KLK15	2.5683929	-3.0893	0.445998	0.0188061	kallikrein related peptidase 15
886	7961059	KLRB1	2.3373124	-3.4312	1.290053	0.0305155	killer cell lectin like receptor B1
887	7953835	KLRG1	2.1594573	-3.6845	1.018282	0.0438027	killer cell lectin like receptor G1
888	7955637	KRT18	-2.5834326	-3.0667	-1.29287	0.0182137	keratin 18
889	8154725	KRT18	-2.2865045	-3.5046	-1.10397	0.0338708	keratin 18
890	8015349	KRT19	-4.0547482	-0.7513	-1.60343	0.0006757	keratin 19
891	8015095	KRT28	-2.3000499	-3.4851	-1.19829	0.0329444	keratin 28
892	8015301	KRT35	-3.4422198	-1.7221	-1.52076	0.0027294	keratin 35
893	7955613	KRT7	-2.1998963	-3.6278	-0.89388	0.0403844	keratin 7
894	7963567	KRT8	-2.8190162	-2.706	-1.38937	0.0109569	keratin 8
895	7963333	KRT80	-3.2616388	-2.0096	-2.17972	0.0041033	keratin 80
896	7938309	KRT8P41	-3.0504423	-2.344	-0.76691	0.0065804	keratin 8 pseudogene 41
897	8069848	KRTAP6-2	2.9504408	-2.5012	0.484019	0.0082115	keratin associated protein 6-2
898	8036072	KRTDAP	2.0992886	-3.7679	0.450696	0.0493784	keratinocyte differentiation associated protein
899	7979351	KTN1-AS1	-2.4399451	-3.281	-0.72364	0.0246581	KTN1 antisense RNA 1
900	8022145	L3MBTL4	2.5443381	-3.1255	1.677955	0.0197915	l(3)mbt-like 4 (Drosophila)
901	8092348	LAMP3	3.2393411	-2.045	2.043046	0.0043142	lysosomal associated membrane protein 3
902	7901856	LAMTOR5I	-3.3011076	-1.9468	-0.73184	0.0037545	late endosomal/lysosomal adaptor, MAPK and MTOR activator 5 pse
903	8075637	LARGE1	-3.12542	-2.2256	-0.99067	0.0055682	LARGE xylosyl- and glucuronyltransferase 1
904	7939620	LARGE2	2.9700648	-2.4704	1.354037	0.0078633	LARGE xylosyl- and glucuronyltransferase 2
905	7994541	LAT	3.4038466	-1.7833	0.943889	0.0029771	linker for activation of T-cells
906	7924603	LBR	2.6281842	-2.9989	0.846315	0.0165537	lamin B receptor
907	7905525	LCE1B	2.1423214	-3.7084	0.726464	0.0453293	late cornified envelope 1B
908	7899753	LCK	2.2977887	-3.4883	1.091668	0.0330974	LCK proto-oncogene, Src family tyrosine kinase
909	7988077	LCMT2	-3.5964566	-1.4764	-0.69399	0.0019229	leucine carboxyl methyltransferase 2
910	7938777	LDHA	-2.3782899	-3.3716	-0.52753	0.0280367	lactate dehydrogenase A
911	7961693	LDHB	2.6519066	-2.9629	1.009218	0.0157329	lactate dehydrogenase B
912	8083576	LEKR1	-2.9950533	-2.4312	-1.11477	0.0074405	leucine, glutamate and lysine rich 1
913	7988838	LEO1	-4.6128574	0.1071	-0.72916	0.0001897	LEO1 homolog, Paf1/RNA polymerase II complex component
914	7902074	LEPROT///	-2.1593686	-3.6846	-0.56159	0.0438105	leptin receptor overlapping transcript///leptin receptor
915	8075956	LGALS2	2.2125098	-3.61	0.726456	0.0393692	galectin 2
916	7910706	LGALS8	-2.2199095	-3.5995	-0.62542	0.0387846	galectin 8
917	8149685	LGI3	-2.4019297	-3.337	-0.51006	0.0266933	leucine rich repeat LGI family member 3
918	8039246	LILRA4	2.2740432	-3.5224	0.992378	0.0347442	leukocyte immunoglobulin like receptor A4
919	8180196	LILRA6	3.5758633	-1.5092	0.809081	0.0020151	leukocyte immunoglobulin like receptor A6
920	8017393	LIMD2	2.9499235	-2.502	0.837733	0.0082208	LIM domain containing 2
921	8139873	LINC00174	-2.3437364	-3.4219	-1.0286	0.0301139	long intergenic non-protein coding RNA 174
922	7898736	LINC0033!	-2.4714046	-3.2344	-0.77697	0.0230846	long intergenic non-protein coding RNA 339
923	7909561	LINC00467	-3.6654655	-1.3666	-0.9356	0.0016433	long intergenic non-protein coding RNA 467
924	8021995	LINC0047C	-2.654339	-2.9592	-3.43031	0.015651	long intergenic non-protein coding RNA 470
925	8162562	LINC00476	2.5733557	-3.0819	0.528149	0.0186086	long intergenic non-protein coding RNA 476
926	7961820	LINC00477	-2.2938833	-3.494	-0.56858	0.0333632	long intergenic non-protein coding RNA 477
927	8099438	LINC00504	-2.7143645	-2.8674	-2.14649	0.0137528	long intergenic non-protein coding RNA 504
928	8101359	LINC00575	2.3733901	-3.3787	0.568335	0.028323	long intergenic non-protein coding RNA 575
929	7927099	LINC0083!	2.5171177	-3.1663	0.52882	0.0209651	long intergenic non-protein coding RNA 839
930	8026503	LINC00905	2.8261247	-2.695	0.622268	0.0107882	long intergenic non-protein coding RNA 905
931	8116607	LINC01011	-2.1991942	-3.6288	-0.53368	0.0404416	long intergenic non-protein coding RNA 1011
932	8056730	LINC01124	-2.1441821	-3.7058	-1.27554	0.0451612	long intergenic non-protein coding RNA 1124
933	8093872	LINC01587	3.2713875	-1.9941	0.767499	0.0040143	long intergenic non-protein coding RNA 1587
934	8065547	LINC01597	2.930555	-2.5323	0.588969	0.0085795	long intergenic non-protein coding RNA 1597
935	8092541	LIPH	-2.9163072	-2.5546	-1.3684	0.0088529	lipase H
936	8053975	LMAN2L	-2.1955746	-3.6339	-0.55503	0.0407377	lectin, mannose binding 2 like
937	8111533	LMBRD2	-2.2543648	-3.5505	-0.52493	0.0361659	LMBR1 domain containing 2
938	8085033	LMLN	-2.7567985	-2.8022	-0.9411	0.0125455	leishmanolysin like peptidase
939	7921936	LMX1A	2.1168514	-3.7437	0.404339	0.0476878	LIM homeobox transcription factor 1 alpha
940	8157922	LMX1B	-2.2424701	-3.5675	-1.11611	0.037051	LIM homeobox transcription factor 1 beta
941	8100362	LNX1	-3.0549794	-2.3369	-1.08343	0.0065143	ligand of numb-protein X 1
942	7970716	LNX2	-2.1796655	-3.6562	-0.59371	0.0420628	ligand of numb-protein X 2
943	8025124	LOC10003	-2.4802321	-3.2213	-2.85584	0.0226602	uncharacterized LOC100034248///microtubule affinity regulating kin
944	8052376	LOC10012	-3.0961131	-2.272	-1.51582	0.0059444	uncharacterized LOC100129434
945	8137962	LOC10012	2.1567951	-3.6882	0.425863	0.0440368	uncharacterized LOC100129484
946	8103092	LOC10012	2.1041668	-3.7612	0.518344	0.0489035	uncharacterized LOC100129572
947	8127776	LOC10013	2.1010292	-3.7655	0.486263	0.0492085	PRO2122

948	7991047	LOC10013	-2.8148654	-2.7125	-0.98015	0.0110566	uncharacterized LOC100131860
949	7976554	LOC10013.	2.7265912	-2.8486	0.764286	0.013394	uncharacterized LOC100133207
950	7978831	LOC10028	2.859497	-2.6432	0.547151	0.0100288	uncharacterized LOC100288910
951	8117176	LOC10050	2.2901367	-3.4993	0.532636	0.0336201	putative UPF0607 protein FLJ37424///UPF0607 protein ENSP0000038
952	8088636	LOC10050	-2.1347153	-3.7189	-0.80777	0.0460222	HHSL751
953	8100768	LOC10192	-2.2500897	-3.5566	-5.49773	0.0364817	UDP-glucuronosyltransferase 2B10-like///UDP glucuronosyltransferas
954	8086024	LOC10537	2.205402	-3.62	0.47162	0.0399384	uncharacterized LOC105377021
955	8148982	LOC10537	2.1082041	-3.7556	0.757257	0.0485135	uncharacterized LOC105377774
956	8161476	LOC10798	2.2957536	-3.4913	0.485612	0.0332357	uncharacterized LOC107987020///uncharacterized LOC107987020
957	7909418	LOC14869	-3.8700625	-1.0421	-1.09164	0.0010303	uncharacterized LOC148696
958	8089464	LOC15176	2.1949954	-3.6347	1.754495	0.0407853	putative uncharacterized protein LOC151760
959	8012893	LOC40057	2.2010857	-3.6261	0.449317	0.0402876	uncharacterized LOC400576
960	8147721	LOC40147.	3.4693087	-1.679	0.579339	0.0025669	myosin IC heavy chain
961	7991577	LOC44031	2.3331615	-3.4373	0.590745	0.0307776	protein enabled homolog
962	8130807	LOC44117	2.1835765	-3.6507	3.145426	0.0417334	uncharacterized LOC441178
963	8130980	LOC44117	2.1835765	-3.6507	3.145426	0.0417334	uncharacterized LOC441178
964	7953844	LOC64284	2.2274682	-3.5888	0.699177	0.0381956	DEAD/H (Asp-Glu-Ala-Asp/His) box polypeptide 11-like///DEAD/H-b
965	7954655	LOC64284	2.2059259	-3.6193	0.715485	0.0398961	DEAD/H (Asp-Glu-Ala-Asp/His) box polypeptide 11-like///DEAD/H-b
966	7961031	LOC64284	2.2059259	-3.6193	0.715485	0.0398961	DEAD/H (Asp-Glu-Ala-Asp/His) box polypeptide 11-like///DEAD/H-b
967	8016261	LOC64417.	2.4935043	-3.2015	0.628509	0.022036	mitogen-activated protein kinase 8 interacting protein 1 pseudogene
968	8007848	LOC64417.	2.4550368	-3.2587	0.643884	0.0238911	mitogen-activated protein kinase 8 interacting protein 1 pseudogene
969	7912280	LOC64501	-2.1502933	-3.6973	-1.54005	0.0446132	uncharacterized LOC645010
970	8060790	LOC72828	2.1127721	-3.7493	0.480835	0.0480757	uncharacterized LOC728283
971	7993776	LOC81691	-2.2206707	-3.5984	-0.8964	0.0387249	exonuclease NEF-sp
972	8040340	LPIN1	2.4948181	-3.1996	1.145982	0.0219751	lipin 1
973	8088642	LRIG1	-2.4903349	-3.2063	-1.02273	0.0221836	leucine rich repeats and immunoglobulin like domains 1
974	7961339	LRP6	2.9034477	-2.5747	0.868165	0.0091068	LDL receptor related protein 6
975	8092009	LRRC34	2.3824235	-3.3655	0.915759	0.0277974	leucine rich repeat containing 34
976	8008040	LRRC46	-2.9476207	-2.5056	-1.36966	0.0082627	leucine rich repeat containing 46
977	7984569	LRRC49	-2.8089172	-2.7217	-1.00647	0.0112009	leucine rich repeat containing 49
978	8152962	LRRC6	-2.3946684	-3.3476	-1.08298	0.0270994	leucine rich repeat containing 6
979	8049532	LRRFIP1	-3.3223155	-1.9131	-0.98642	0.0035792	LRR binding FLII interacting protein 1
980	8049512	LRRFIP1	-2.9209081	-2.5474	-0.46578	0.0087637	LRR binding FLII interacting protein 1
981	7954810	LRRK2	2.8640294	-2.6361	1.002742	0.0099297	leucine rich repeat kinase 2
982	7942315	LRTOMT	-2.8792084	-2.6125	-1.32366	0.0096047	leucine rich transmembrane and O-methyltransferase domain contai
983	8138912	LSM5	-2.8786299	-2.6134	-0.71459	0.0096169	LSM5 homolog, U6 small nuclear RNA and mRNA degradation assoc
984	7937735	LSP1	2.402075	-3.3367	0.876399	0.0266852	lymphocyte-specific protein 1
985	8177976	LTA	2.3260312	-3.4476	0.455683	0.0312328	lymphotoxin alpha
986	7987650	LTK	3.1093991	-2.251	0.635662	0.0057709	leukocyte receptor tyrosine kinase
987	8107429	LVRN	-2.2700528	-3.5281	-2.73609	0.0350283	laeverin
988	7906622	LY9	2.7309385	-2.842	1.59663	0.0132686	lymphocyte antigen 9
989	8045674	LYPD6	-2.3775489	-3.3727	-1.3519	0.0280799	LY6/PLAUR domain containing 6
990	8045664	LYPD6B	-3.4288802	-1.7434	-1.5658	0.0028131	LY6/PLAUR domain containing 6B
991	7919923	LYSMD1	-2.7119998	-2.871	-0.82492	0.0138232	LysM domain containing 1
992	7988833	LYSMD2	2.9049141	-2.5724	0.856839	0.0090775	LysM domain containing 2
993	7912292	LZIC	-2.8763825	-2.6169	-0.63089	0.0096645	leucine zipper and CTNNBIP1 domain containing
994	8086555	LZTFL1	-2.8445369	-2.6664	-0.86639	0.0103626	leucine zipper transcription factor like 1
995	8138466	MACC1	-2.4500618	-3.266	-1.09168	0.0241414	metastasis associated in colon cancer 1
996	7949033	MACROD1	-2.2379548	-3.5739	-1.21161	0.0373921	MACRO domain containing 1
997	8019798	MAFG	2.221214	-3.5977	0.455223	0.0386823	MAF bZIP transcription factor G
998	8131091	MAFK	-2.4894529	-3.2076	-0.53794	0.0222248	MAF bZIP transcription factor K
999	8167815	MAGED2	-2.3050688	-3.4778	-1.13049	0.0326072	MAGE family member D2
1000	8123893	MAK	-2.4449037	-3.2737	-1.35381	0.0244036	male germ cell associated kinase
1001	8043504	MAL	2.3106684	-3.4698	0.898154	0.0322347	mal, T-cell differentiation protein
1002	8148040	MAL2	-2.3040011	-3.4794	-1.2365	0.0326786	mal, T-cell differentiation protein 2 (gene/pseudogene)
1003	8170420	MAMLD1	3.0456013	-2.3517	1.523504	0.0066515	mastermind like domain containing 1
1004	8093916	MAN2B2	-2.5116223	-3.1745	-0.641	0.0212099	mannosidase alpha class 2B member 2
1005	7983228	MAP1A	2.1347621	-3.7189	0.587586	0.0460179	microtubule associated protein 1A
1006	8005707	MAP2K3	2.5485556	-3.1192	0.664841	0.0196152	mitogen-activated protein kinase kinase 3
1007	8036525	MAP4K1	2.3310263	-3.4403	1.082144	0.0309133	mitogen-activated protein kinase kinase kinase kinase 1
1008	8000811	MAPK3	-2.7710678	-2.7802	-0.71556	0.0121628	mitogen-activated protein kinase 3
1009	8021199	MAPK4	2.4524244	-3.2625	0.713938	0.0240223	mitogen-activated protein kinase 4
1010	8074108	MAPK8IP2	-2.3407975	-3.4262	-3.56823	0.030297	mitogen-activated protein kinase 8 interacting protein 2
1011	8116402	MAPK9	-4.8192031	0.4144	-0.88956	0.0001191	mitogen-activated protein kinase 9
1012	8020878	MAPRE2	2.3962517	-3.3453	0.744027	0.0270104	microtubule associated protein RP/EB family member 2
1013	7909866	MARC2///I	-2.5331364	-3.1423	-0.93247	0.0202667	mitochondrial amidoxime reducing component 2///mitochondrial an
1014	8105899	MARVELD2	-2.9362945	-2.5233	-1.05156	0.0084717	MARVEL domain containing 2
1015	8177498	MARVELD2	-2.9362945	-2.5233	-1.05156	0.0084717	MARVEL domain containing 2
1016	8105741	MAST4	-2.8297036	-2.6894	-1.03479	0.0107042	microtubule associated serine/threonine kinase family member 4
1017	8150099	MBOAT4	-2.2026733	-3.6239	-0.81182	0.0401589	membrane bound O-acyltransferase domain containing 4
1018	8106068	MCCC2	-2.6201907	-3.0111	-0.98993	0.0168393	methylcrotonoyl-CoA carboxylase 2
1019	8177601	MCCC2	-2.6201907	-3.0111	-0.98993	0.0168393	methylcrotonoyl-CoA carboxylase 2
1020	8146738	MCMDC2	-2.9082237	-2.5672	-1.14338	0.0090117	minichromosome maintenance domain containing 2

1021	7914996	MEAF6	-2.4827976	-3.2175	-0.48822	0.0225383	MYST/Esa1 associated factor 6
1022	8032106	MED16	-2.5572816	-3.1061	-0.48393	0.0192553	mediator complex subunit 16
1023	8007643	MEIOC	-2.7456365	-2.8194	-1.20936	0.0128529	meiosis specific with coiled-coil domain
1024	8005695	MEIS3///M	-3.7792318	-1.1859	-1.24284	0.0012678	Meis homeobox 3///Meis homeobox 3 pseudogene 1
1025	8005132	MEIS3///M	-3.068973	-2.3148	-1.08335	0.0063147	Meis homeobox 3///Meis homeobox 3 pseudogene 1
1026	8096290	MEPE	-2.2690551	-3.5296	-1.66848	0.0350996	matrix extracellular phosphoglycoprotein
1027	7991332	MESP1	2.2729119	-3.524	0.409869	0.0348246	mesoderm posterior bHLH transcription factor 1
1028	8136248	MEST	2.6225733	-3.0074	1.217636	0.0167537	mesoderm specific transcript
1029	7957404	METTL25	-2.4765969	-3.2267	-1.13013	0.0228341	methyltransferase like 25
1030	8009008	METTL2A	-2.1643569	-3.6777	-0.52374	0.0433749	methyltransferase like 2A
1031	7988245	MFAP1	-3.2193391	-2.0768	-0.51158	0.0045124	microfibrillar associated protein 1
1032	8109403	MFAP3	-3.3106271	-1.9317	-0.56872	0.0036748	microfibrillar associated protein 3
1033	7897824	MFN2	-2.2826854	-3.51	-0.50506	0.0341363	mitofusin 2
1034	8040516	MFSD2B	2.7093264	-2.8751	0.759104	0.0139033	major facilitator superfamily domain containing 2B
1035	8116484	MGAT1	2.7384974	-2.8303	0.478223	0.0130532	mannosyl (alpha-1,3-)-glycoprotein beta-1,2-N-acetylglucosaminyltra
1036	8073129	MGAT3	3.0557844	-2.3356	0.627569	0.0065027	mannosyl (beta-1,4-)-glycoprotein beta-1,4-N-acetylglucosaminyltran
1037	8061129	MGME1///	-2.1925343	-3.6381	-0.44788	0.0409879	mitochondrial genome maintenance exonuclease 1///mitochondrial g
1038	8097513	MGST2	-2.1373308	-3.7153	-0.63471	0.0457829	microsomal glutathione S-transferase 2
1039	8028924	MIA-RAB4	2.1710652	-3.6683	1.471985	0.0427954	MIA-RAB4B readthrough (NMD candidate)///melanoma inhibitory ac
1040	7938519	MICALCL	-3.5764533	-1.5082	-1.27896	0.0020124	MICAL C-terminal like
1041	7970546	MICU2	-2.3957334	-3.3461	-0.48163	0.0270395	mitochondrial calcium uptake 2
1042	8166797	MID1IP1	-2.3127575	-3.4668	-0.84202	0.0320967	MID1 interacting protein 1
1043	8018343	MIF4GD	-2.1152963	-3.7458	-0.79079	0.0478353	MIF4G domain containing
1044	7970577	MIPEP	-2.2257602	-3.5912	-0.73654	0.038328	mitochondrial intermediate peptidase
1045	7973985	MIPOL1	-2.7197854	-2.8591	-0.84578	0.0135926	mirror-image polydactyly 1
1046	7957735	MIR135A2	-2.1321901	-3.7224	-2.5455	0.0462544	microRNA 135a-2
1047	7976806	MIR136	-3.5106956	-1.613	-1.46394	0.0023368	microRNA 136
1048	7997008	MIR140	2.1111208	-3.7516	0.516087	0.0482336	microRNA 140
1049	8059211	MIR153-1	2.1165317	-3.7441	0.564116	0.0477181	microRNA 153-1
1050	8068022	MIR155///I	2.6955341	-2.8963	0.974506	0.0143233	microRNA 155///MIR155 host gene
1051	7949273	MIR192	-2.8214394	-2.7023	-0.82237	0.0108991	microRNA 192
1052	8142977	MIR29B1	-2.3470543	-3.4171	-3.44367	0.0299084	microRNA 29b-1
1053	8013784	MIR451A	-2.5920079	-3.0537	-0.83054	0.0178839	microRNA 451a
1054	7957608	MIR492///I	-2.8207027	-2.7034	-1.53531	0.0109166	microRNA 492///keratin 19 pseudogene 2
1055	8031035	MIR516B1	-2.5714866	-3.0847	-4.86377	0.0186828	microRNA 516b-1
1056	8031037	MIR519A2,	-3.1605841	-2.17	-6.61461	0.0051474	microRNA 519a-2///microRNA 517c
1057	7969574	MIR622///I	-2.3784406	-3.3714	-1.14523	0.028028	microRNA 622///keratin 18
1058	8067932	MIR99AHG	2.2978323	-3.4883	2.10716	0.0330945	mir-99a-let-7c cluster host gene
1059	8030842	MIR99B	2.9654717	-2.4776	0.640717	0.0079435	microRNA 99b
1060	8073816	MIRLET7BH	-2.8299813	-2.689	-1.03147	0.0106977	MIRLET7B host gene
1061	8024013	MISP	-2.8978494	-2.5834	-1.91898	0.0092196	mitotic spindle positioning
1062	8064967	MKKS	-2.2145383	-3.6071	-0.51995	0.0392081	McKusick-Kaufman syndrome
1063	8016909	MKS1	-2.957008	-2.4909	-0.61558	0.0080933	Meckel syndrome, type 1
1064	7969093	MLNR	2.6710712	-2.9336	0.504064	0.0150982	motilin receptor
1065	8055672	MMADHC	-3.2618227	-2.0093	-0.67229	0.0041016	methylmalonic aciduria and homocystinuria, cblD type
1066	8160521	MOB3B	2.5511675	-3.1153	1.273385	0.0195068	MOB kinase activator 3B
1067	8048725	MOGAT1	3.1453395	-2.1941	0.714984	0.0053259	monoacylglycerol O-acyltransferase 1
1068	8141676	MOGAT3	2.3734217	-3.3787	0.446663	0.0283211	monoacylglycerol O-acyltransferase 3
1069	7911701	MORN1	-2.9938258	-2.4331	-0.97826	0.0074607	MORN repeat containing 1
1070	8058824	MREG	-2.2716427	-3.5259	-0.74104	0.0349149	melanoregulin
1071	8093936	MRFAP1	-3.0671487	-2.3177	-0.62458	0.0063404	Morf4 family associated protein 1
1072	8153426	MROH6	2.3016526	-3.4828	0.642652	0.0328364	maestro heat like repeat family member 6
1073	8016366	MRPL10	-2.2609396	-3.5412	-0.39976	0.035685	mitochondrial ribosomal protein L10
1074	7937763	MRPL23	2.4580516	-3.2542	0.580896	0.0237406	mitochondrial ribosomal protein L23
1075	8043278	MRPL35	-2.2021491	-3.6246	-0.52295	0.0402013	mitochondrial ribosomal protein L35
1076	8053165	MRPL53	-2.7175192	-2.8626	-0.7057	0.0136594	mitochondrial ribosomal protein L53
1077	8085531	MRPS25	2.3378142	-3.4305	0.701636	0.030484	mitochondrial ribosomal protein S25
1078	8105852	MRPS36	-2.1833057	-3.6511	-0.39183	0.0417562	mitochondrial ribosomal protein S36
1079	7940287	MS4A1	3.4143608	-1.7665	2.991084	0.0029071	membrane spanning 4-domains A1
1080	7940299	MS4A12	-2.1361067	-3.717	-0.70315	0.0458948	membrane spanning 4-domains A12
1081	8093756	MSANTD1	2.2311642	-3.5836	0.394157	0.0379106	Myb/SANT DNA binding domain containing 1
1082	7967039	MSI1	2.8456297	-2.6647	0.78021	0.0103379	musashi RNA binding protein 1
1083	8166015	MSL3	2.9721424	-2.4672	0.534173	0.0078273	male-specific lethal 3 homolog (Drosophila)
1084	7927529	MSMB	-2.2605555	-3.5417	-3.43244	0.035713	microseminoprotein beta
1085	8087547	MST1R	-4.5742875	0.049	-2.05626	0.000207	macrophage stimulating 1 receptor
1086	8110084	MSX2	-2.6736525	-2.9297	-1.78612	0.0150146	msh homeobox 2
1087	8008716	MSX2P1	2.1851917	-3.6485	0.629141	0.0415981	msh homeobox 2 pseudogene 1
1088	7995806	MT1A	-2.6074286	-3.0304	-1.00958	0.017305	metallothionein 1A
1089	7995820	MT1B	-2.380541	-3.3683	-0.66748	0.0279062	metallothionein 1B
1090	8001531	MT1G	-2.7311277	-2.8417	-1.1426	0.0132632	metallothionein 1G
1091	7995829	MT1H	-2.2458087	-3.5627	-0.88342	0.0368005	metallothionein 1H
1092	7995803	MT1JP	-2.3979264	-3.3428	-0.77239	0.0269165	metallothionein 1J, pseudogene
1093	7995787	MT1M	-2.5180743	-3.1649	-0.41081	0.0209228	metallothionein 1M

1094	8095362	MT2A	-2.2362546	-3.5763	-0.76117	0.0375213	metallothionein 2A
1095	8095376	MT2A	-2.2362546	-3.5763	-0.76117	0.0375213	metallothionein 2A
1096	7966046	MTERF2	-2.8899681	-2.5957	-0.54843	0.0093806	mitochondrial transcription termination factor 2
1097	7912412	MTOR	-2.2014004	-3.6257	-0.38679	0.0402621	mechanistic target of rapamycin
1098	8130383	MTRF1L	-2.334766	-3.4349	-0.67387	0.0306761	mitochondrial translational release factor 1 like
1099	8098705	MTRF1L	-2.236483	-3.576	-0.61787	0.0375039	mitochondrial translational release factor 1 like
1100	8046573	MTX2	-2.5064317	-3.1823	-0.5943	0.0214435	metaxin 2
1101	7947156	MUC15	2.2608074	-3.5414	2.008025	0.0356946	mucin 15, cell surface associated
1102	8135048	MUC17	-2.258998	-3.5439	-3.16964	0.0358264	mucin 17, cell surface associated
1103	8135015	MUC3B///I	2.152234	-3.6946	0.494217	0.0444404	mucin 3B, cell surface associated///mucin 3A, cell surface associated
1104	8169145	MUM1L1	-2.2521026	-3.5538	-1.53661	0.0363327	MUM1 like 1
1105	8026751	MVB12A	-2.3260827	-3.4475	-0.55179	0.0312294	multivesicular body subunit 12A
1106	8122202	MYB///MY	-3.4290078	-1.7432	-2.43626	0.0028123	MYB proto-oncogene, transcription factor///MYB proto-oncogene, tr
1107	8012625	MYH13	2.4442757	-3.2746	0.585271	0.0244357	myosin heavy chain 13
1108	7956211	MYL6	-2.1057819	-3.7589	-0.43025	0.0487471	myosin light chain 6
1109	8033605	MYO1F	2.1944437	-3.6355	0.79732	0.0408306	myosin IF
1110	8023267	MYO5B	-2.3566164	-3.4032	-0.86312	0.0293235	myosin VB
1111	7988876	MYO5C	-3.7242642	-1.2731	-0.99929	0.0014372	myosin VC
1112	8120783	MYO6	-2.7761299	-2.7724	-0.72241	0.0120297	myosin VI
1113	8114511	MZB1	2.2279938	-3.588	1.528367	0.038155	marginal zone B and B1 cell specific protein
1114	7956220	NABP2	-2.4163137	-3.3158	-0.65514	0.0259055	nucleic acid binding protein 2
1115	7972601	NALCN	3.1430209	-2.1978	1.437633	0.0053535	sodium leak channel, non-selective
1116	8144857	NAT1	-3.270153	-1.9961	-2.30624	0.0040255	N-acetyltransferase 1
1117	8141669	NAT16	2.6591265	-2.9519	0.725737	0.0154909	N-acetyltransferase 16 (putative)
1118	8093578	NAT8L	2.3054473	-3.4773	0.728425	0.0325819	N-acetyltransferase 8 like
1119	8047606	NBEAL1	-2.4177751	-3.3137	-0.50299	0.0258266	neurobeachin like 1
1120	8007471	NBR1	-2.6761132	-2.9259	-0.6494	0.0149353	NBR1, autophagy cargo receptor
1121	8152119	NCALD	3.2719752	-1.9932	1.92483	0.004009	neurocalcin delta
1122	8067985	NCAM2	-2.5250202	-3.1545	-1.59227	0.0206178	neural cell adhesion molecule 2
1123	8133518	NCF1C///N	2.2560285	-3.5482	1.013463	0.0360436	neutrophil cytosolic factor 1C pseudogene///neutrophil cytosolic fac
1124	8140227	NCF1C///N	2.2560285	-3.5482	1.013463	0.0360436	neutrophil cytosolic factor 1C pseudogene///neutrophil cytosolic fac
1125	8072744	NCF4	2.2284654	-3.5874	1.135975	0.0381185	neutrophil cytosolic factor 4
1126	8057517	NCKAP1	-2.5489661	-3.1186	-0.50553	0.0195982	NCK associated protein 1
1127	7977621	NDRG2	2.3703035	-3.3832	1.436135	0.0285047	NDRG family member 2
1128	7987642	NDUFAF1	-2.9111022	-2.5627	-0.84744	0.0089548	NADH:ubiquinone oxidoreductase complex assembly factor 1
1129	8105499	NDUFAF2	2.2076564	-3.6168	0.436152	0.039757	NADH:ubiquinone oxidoreductase complex assembly factor 2
1130	8081945	NDUFB4	-2.7922404	-2.7475	-0.63897	0.0116154	NADH:ubiquinone oxidoreductase subunit B4
1131	8093314	NDUFB4	-2.7922404	-2.7475	-0.63897	0.0116154	NADH:ubiquinone oxidoreductase subunit B4
1132	8058428	NDUFS1	-2.1133585	-3.7485	-0.35975	0.0480198	NADH:ubiquinone oxidoreductase core subunit S1
1133	8029507	NECTIN2	-2.5450462	-3.1245	-0.9045	0.0197618	nectin cell adhesion molecule 2
1134	7957715	NEDD1	-2.1502005	-3.6974	-0.49062	0.0446214	neural precursor cell expressed, developmentally down-regulated 1
1135	8082643	NEK11	-5.8997882	1.9117	-1.66468	0.0000111	NIMA related kinase 11
1136	7971757	NEK5	-2.9357988	-2.5241	-1.38023	0.0084809	NIMA related kinase 5
1137	7908543	NEK7	-2.5397847	-3.1324	-0.61093	0.0199834	NIMA related kinase 7
1138	7984704	NEO1	-2.4055883	-3.3316	-0.78698	0.0264908	neogenin 1
1139	7921088	NES	2.988997	-2.4407	1.508793	0.0075409	nestin
1140	8024623	NFIC	-2.4850259	-3.2142	-0.62616	0.0224329	nuclear factor I C
1141	7930074	NFKB2	2.3941408	-3.3484	0.572938	0.0271292	nuclear factor kappa B subunit 2
1142	8126666	NFKBIE	2.2882028	-3.5021	0.428611	0.0337534	NFKB inhibitor epsilon
1143	7965956	NFYB	-2.3175058	-3.4599	-0.45279	0.0317851	nuclear transcription factor Y subunit beta
1144	7985983	NGRN	-3.0040071	-2.4171	-0.6586	0.0072944	neugrin, neurite outgrowth associated
1145	8162455	NINJ1	-2.1136283	-3.7481	-0.66251	0.0479941	ninjurin 1
1146	8094938	NIPAL1	2.3208273	-3.4551	0.855559	0.0315689	NIPA like domain containing 1
1147	7906652	NIT1	-2.4377665	-3.2842	-0.61315	0.0247707	nitrilase 1
1148	8081277	NIT2	-2.3249278	-3.4492	-0.47769	0.0313037	nitrilase family member 2
1149	8039557	NLRP13	2.2375478	-3.5745	0.536565	0.037423	NLR family pyrin domain containing 13
1150	8039280	NLRP7	2.4716333	-3.2341	0.585665	0.0230735	NLR family pyrin domain containing 7
1151	8083757	NMD3	-2.2033451	-3.6229	-0.43246	0.0401045	NMD3 ribosome export adaptor
1152	8114354	NME5	-3.062936	-2.3243	-5.48583	0.0064001	NME/NM23 family member 5
1153	7932227	NMT2	2.3658408	-3.3898	0.786965	0.0287693	N-myristoyltransferase 2
1154	8022856	NOL4	3.3778735	-1.8246	1.047058	0.0031572	nucleolar protein 4
1155	8046099	NOSTRIN	-2.276434	-3.519	-1.32948	0.0345751	nitric oxide synthase trafficking
1156	8010696	NPB	2.1849793	-3.6488	0.438512	0.0416159	neuropeptide B
1157	8019693	NPB	2.1849793	-3.6488	0.438512	0.0416159	neuropeptide B
1158	7922627	NPHS2	3.2271386	-2.0644	0.685092	0.0044341	NPHS2, podocin
1159	8019074	NPTX1	2.1190407	-3.7407	0.441614	0.0474808	neuronal pentraxin 1
1160	8002303	NQO1	-2.1903574	-3.6412	-1.53075	0.041168	NAD(P)H quinone dehydrogenase 1
1161	7957835	NR1H4	2.7019186	-2.8865	6.09547	0.0141274	nuclear receptor subfamily 1 group H member 4
1162	8169115	NRK	-2.7437495	-2.8223	-1.80426	0.0129056	Nik related kinase
1163	8084951	NRROS	2.146022	-3.7032	0.756583	0.0449955	negative regulator of reactive oxygen species
1164	8060339	NRSN2	-2.133767	-3.7203	-0.67707	0.0461093	neurensin 2
1165	7924172	NSL1	-2.1360719	-3.7171	-0.53052	0.0458979	NSL1, MIS12 kinetochore complex component
1166	8121578	NT5DC1	-2.9683396	-2.4731	-0.77909	0.0078933	5'-nucleotidase domain containing 1

1167	7909142	NUCKS1	-2.6186584	-3.0134	-0.70122	0.0168946	nuclear casein kinase and cyclin dependent kinase substrate 1
1168	8113413	NUDT12	-2.7155812	-2.8655	-0.91412	0.0137167	nudix hydrolase 12
1169	8082667	NUDT16	-5.7749502	1.7491	-1.01613	0.0000145	nudix hydrolase 16
1170	7993019	NUDT16L1	-2.1482723	-3.7001	-0.56277	0.0447937	nudix hydrolase 16 like 1
1171	8149979	NUGGC	2.9840628	-2.4485	0.81894	0.0076237	nuclear GTPase, germinal center associated
1172	7968132	NUP58	-2.8612891	-2.6404	-0.5572	0.0099895	nucleoporin 58
1173	8000574	NUPR1	-2.2422771	-3.5678	-0.74499	0.0370655	nuclear protein 1, transcriptional regulator
1174	8131506	NXPH1	-2.2581413	-3.5452	-4.32506	0.035889	neurexophilin 1
1175	7986838	OCA2	2.333744	-3.4364	0.644731	0.0307407	OCA2 melanosomal transmembrane protein
1176	8105908	OCLN///LC	-3.9036623	-0.989	-1.24866	0.0009542	occludin///occludin pseudogene
1177	8095456	ODAM	2.5449254	-3.1246	1.166492	0.0197668	odontogenic, ameloblast asssociated
1178	8033818	OLFM2	4.6058987	0.0966	1.215024	0.0001927	olfactomedin 2
1179	8014057	OMG	-2.6757431	-2.9265	-2.09101	0.0149472	oligodendrocyte myelin glycoprotein
1180	8021357	ONECUT2	2.1300216	-3.7254	0.441129	0.0464547	one cut homeobox 2
1181	7938172	OR10A5	-3.348731	-1.871	-4.14507	0.0033721	olfactory receptor family 10 subfamily A member 5
1182	7977767	OR10G3	-2.1815029	-3.6536	-2.27945	0.0419078	olfactory receptor family 10 subfamily G member 3
1183	8178289	OR12D3	-2.9930069	-2.4344	-0.82415	0.0074743	olfactory receptor family 12 subfamily D member 3
1184	8124645	OR12D3	-2.2347485	-3.5785	-0.55557	0.0376361	olfactory receptor family 12 subfamily D member 3
1185	7992863	OR1F1	2.6638545	-2.9447	0.566348	0.0153343	olfactory receptor family 1 subfamily F member 1
1186	8157698	OR1K1	-2.4032213	-3.3351	-0.70849	0.0266216	olfactory receptor family 1 subfamily K member 1
1187	8157679	OR1Q1	-2.2486832	-3.5586	-0.95981	0.0365862	olfactory receptor family 1 subfamily Q member 1
1188	7911265	OR2M2	-2.1574624	-3.6873	-1.45685	0.043978	olfactory receptor family 2 subfamily M member 2
1189	7925755	OR2T11	2.2573154	-3.5463	0.668133	0.0359494	olfactory receptor family 2 subfamily T member 11 (gene/pseudoger
1190	7940177	OR4D10	2.5496479	-3.1175	0.770839	0.0195698	olfactory receptor family 4 subfamily D member 10
1191	7973296	OR4E2	-2.1286542	-3.7273	-2.59758	0.0465813	olfactory receptor family 4 subfamily E member 2
1192	7896744	OR4F29///	-2.8231281	-2.6996	-1.42584	0.010859	olfactory receptor family 4 subfamily F member 29///olfactory recep
1193	7911345	OR4F29///	-2.8231281	-2.6996	-1.42584	0.010859	olfactory receptor family 4 subfamily F member 29///olfactory recep
1194	8110672	OR4F29///	-2.8231281	-2.6996	-1.42584	0.010859	olfactory receptor family 4 subfamily F member 29///olfactory recep
1195	8148962	OR4F29///	-2.8231281	-2.6996	-1.42584	0.010859	olfactory receptor family 4 subfamily F member 29///olfactory recep
1196	7946125	OR52B2	-2.0977719	-3.77	-0.93211	0.0495269	olfactory receptor family 52 subfamily B member 2
1197	7937986	OR52J3	-2.3043047	-3.479	-0.59367	0.0326583	olfactory receptor family 52 subfamily J member 3
1198	7937942	OR52K1	-2.1445291	-3.7053	-0.7488	0.0451299	olfactory receptor family 52 subfamily K member 1
1199	7946105	OR52N5	-2.2344826	-3.5788	-4.28869	0.0376564	olfactory receptor family 52 subfamily N member 5
1200	8081192	OR5AC2	2.48792	-3.2099	0.723675	0.0222967	olfactory receptor family 5 subfamily AC member 2
1201	7938261	OR5E1P	3.0476319	-2.3485	0.656979	0.0066216	olfactory receptor family 5 subfamily E member 1 pseudogene
1202	8060084	OR6B3	-2.3264882	-3.4469	-0.41448	0.0312034	olfactory receptor family 6 subfamily B member 3
1203	7973303	OR6C4	2.6776141	-2.9237	1.405841	0.0148871	olfactory receptor family 6 subfamily C member 4
1204	7952373	OR6X1	3.3738786	-1.831	0.662136	0.0031859	olfactory receptor family 6 subfamily X member 1
1205	8138151	OR7E37P//	2.5294034	-3.1479	0.567655	0.0204275	olfactory receptor family 7 subfamily E member 37 pseudogene///M
1206	8156319	OR7E37P//	2.1588312	-3.6854	0.493935	0.0438577	olfactory receptor family 7 subfamily E member 37 pseudogene///M
1207	8082244	OR7E37P//	2.288651	-3.5015	0.537748	0.0337224	olfactory receptor family 7 subfamily E member 37 pseudogene///ol
1208	8082248	OR7E37P//	2.1751309	-3.6626	0.500396	0.0424476	olfactory receptor family 7 subfamily E member 37 pseudogene///ol
1209	7971241	OR7E37P//	2.1276083	-3.7288	0.491846	0.0466784	olfactory receptor family 7 subfamily E member 37 pseudogene///ol
1210	8045887	OR7E37P//	2.1276083	-3.7288	0.491846	0.0466784	olfactory receptor family 7 subfamily E member 37 pseudogene///ol
1211	7939994	OR9G9///C	-3.1440668	-2.1961	-0.55728	0.005341	olfactory receptor family 9 subfamily G member 9///olfactory recept
1212	7956031	ORMDL2	-2.6187637	-3.0132	-0.76465	0.0168908	ORMDL sphingolipid biosynthesis regulator 2
1213	8046646	OSBPL6	-2.6131564	-3.0217	-1.2819	0.0170945	oxysterol binding protein like 6
1214	7914923	OSCP1	-3.0082589	-2.4104	-0.85135	0.007226	organic solute carrier partner 1
1215	8050497	OSR1	3.0178017	-2.3954	0.901907	0.0070748	odd-skipped related transciption factor 1
1216	8009705	OTOP2	2.4068769	-3.3297	0.5201	0.0264198	otopetrin 2
1217	7976425	OTUB2	-2.1719293	-3.6671	-0.85874	0.0427213	OTU deubiquitinase, ubiquitin aldehyde binding 2
1218	7919699	OTUD7B	-2.423858	-3.3047	-0.67141	0.025501	OTU deubiquitinase 7B
1219	8078173	OXNAD1	2.6371233	-2.9853	0.620224	0.0162398	oxidoreductase NAD binding domain containing 1
1220	8011499	P2RX1	2.8368907	-2.6783	1.231838	0.0105374	purinergic receptor P2X 1
1221	7959267	P2RX4	-2.101263	-3.7652	-0.61869	0.0491857	purinergic receptor P2X 4
1222	8011415	P2RX5-TA)	2.7957969	-2.742	1.50768	0.0115258	P2RX5-TAX1BP3 readthrough (NMD candidate)///purinergic receptor
1223	8091511	P2RY14	2.2749604	-3.5211	1.064913	0.0346792	purinergic receptor P2Y14
1224	8171136	P2RY8	2.8964163	-2.5856	1.386184	0.0092487	purinergic receptor P2Y8
1225	8177026	P2RY8	2.8964163	-2.5856	1.386184	0.0092487	purinergic receptor P2Y8
1226	7915444	P3H1	2.3232457	-3.4516	0.809869	0.0314123	prolyl 3-hydroxylase 1
1227	8079677	P4HTM	-2.7965202	-2.7409	-0.75742	0.0115077	prolyl 4-hydroxylase, transmembrane
1228	7942476	PAAF1	-2.3633887	-3.3933	-1.00906	0.0289156	proteasomal ATPase associated factor 1
1229	8167592	PAGE4	3.146268	-2.1926	0.592888	0.0053148	PAGE family member 4
1230	8115681	PANK3	-2.6406041	-2.9801	-0.6465	0.016119	pantothenate kinase 3
1231	8120215	PAQR8	2.1919797	-3.6389	0.868804	0.0410337	progestin and adipoQ receptor family member 8
1232	8047709	PARD3B	-2.5045944	-3.185	-0.71233	0.0215268	par-3 family cell polarity regulator beta
1233	7897404	PARK7	-3.1480604	-2.1898	-0.54672	0.0052936	Parkinsonism associated deglycase
1234	8082086	PARP15	2.4205044	-3.3097	1.411462	0.02568	poly(ADP-ribose) polymerase family member 15
1235	8073682	PARVG	2.8519068	-2.655	0.903847	0.0101969	parvin gamma
1236	7901804	PATJ	-2.3531866	-3.4082	-0.7368	0.0295321	PATJ, crumbs cell polarity complex component
1237	7988327	PATL2	2.2779904	-3.5168	0.771739	0.0344654	PAT1 homolog 2
1238	7965112	PAWR	-3.53823	-1.5691	-0.7979	0.0021951	pro-apoptotic WT1 regulator
1239	8161211	PAX5	2.9716099	-2.468	1.594964	0.0078365	paired box 5

1240	7973974	PAX9	-3.7408148	-1.2468	-2.22984	0.0013839	paired box 9
1241	8129876	PBOV1	-2.2376947	-3.5743	-2.7562	0.0374119	prostate and breast cancer overexpressed 1
1242	7955817	PCBP2	-2.2327323	-3.5813	-0.46991	0.0377903	poly(rC) binding protein 2
1243	8108720	PCDHB9	2.2525533	-3.5531	1.1251	0.0362994	protocadherin beta 9
1244	7906742	PCP4L1	2.1988985	-3.6292	0.571648	0.0404657	Purkinje cell protein 4 like 1
1245	8060294	PDCD1	2.447914	-3.2692	0.59128	0.0242503	programmed cell death 1
1246	8057004	PDE11A	-2.5615219	-3.0997	-2.3077	0.0190827	phosphodiesterase 11A
1247	8151074	PDE7A	3.1147197	-2.2426	0.952472	0.0057028	phosphodiesterase 7A
1248	8122222	PDE7B	2.2039999	-3.622	1.134567	0.0400515	phosphodiesterase 7B
1249	8088384	PDHB	-2.232946	-3.581	-0.47724	0.0377739	pyruvate dehydrogenase (lipoamide) beta
1250	7991815	PDIA2	2.4767213	-3.2265	0.458206	0.0228281	protein disulfide isomerase family A member 2
1251	8052654	PELI1	2.6027982	-3.0374	0.869946	0.0174769	pellino E3 ubiquitin protein ligase 1
1252	7991323	PEX11A	-2.4929264	-3.2024	-0.9871	0.0220628	peroxisomal biogenesis factor 11 alpha
1253	8014264	PEX12	-2.8537999	-2.652	-0.97484	0.0101547	peroxisomal biogenesis factor 12
1254	7940421	PGA4///PG	2.1037949	-3.7617	0.445061	0.0489395	pepsinogen 4, group I (pepsinogen A)///pepsinogen 3, group I (peps
1255	8113591	PGGT1B	-2.4372501	-3.285	-0.64184	0.0247975	protein geranylgeranyltransferase type I subunit beta
1256	8126916	PGK2	-2.1531072	-3.6934	-3.02643	0.0443629	phosphoglycerate kinase 2
1257	8116932	PHACTR1	2.2932186	-3.4949	1.335593	0.0334087	phosphatase and actin regulator 1
1258	7953812	PHC1	2.5805237	-3.0711	0.930857	0.0183269	polyhomeotic homolog 1
1259	7963869	PHC1	2.5805237	-3.0711	0.930857	0.0183269	polyhomeotic homolog 1
1260	8083850	PHC3	-2.2270544	-3.5894	-1.10021	0.0382276	polyhomeotic homolog 3
1261	7897322	PHF13	-2.4544163	-3.2596	-0.62643	0.0239222	PHD finger protein 13
1262	8046186	PHOSPHO.	-2.2932705	-3.4948	-0.85721	0.0334051	PHOSPHO2-KLHL23 readthrough///phosphatase, orphan 2///kelch lik
1263	8159441	PHPT1	-2.300441	-3.4845	-0.38623	0.032918	phosphohistidine phosphatase 1
1264	7960682	PIANP	2.5164735	-3.1673	0.404419	0.0209937	PILR alpha associated neural protein
1265	7989680	PIF1	2.4960975	-3.1977	0.428238	0.0219159	PIF1 5'-to-3' DNA helicase
1266	7983811	PIGB	-3.1044856	-2.2587	-0.6184	0.0058345	phosphatidylinositol glycan anchor biosynthesis class B
1267	7899244	PIGV	-2.2937216	-3.4942	-0.68562	0.0333743	phosphatidylinositol glycan anchor biosynthesis class V
1268	8084955	PIGX	-3.0972715	-2.2701	-0.86016	0.0059291	phosphatidylinositol glycan anchor biosynthesis class X
1269	7951672	PIH1D2	-3.8345561	-1.0982	-1.96877	0.0011173	PIH1 domain containing 2
1270	8090639	PIK3R4	-2.1618241	-3.6812	-0.47677	0.0435956	phosphoinositide-3-kinase regulatory subunit 4
1271	8172471	PIM2	4.0600012	-0.7431	1.544748	0.0006676	Pim-2 proto-oncogene, serine/threonine kinase
1272	7956524	PIP4K2C	-2.4676034	-3.24	-0.80254	0.0232696	phosphatidylinositol-5-phosphate 4-kinase type 2 gamma
1273	8139534	PKD1L1	-2.1053115	-3.7596	-1.11657	0.0487926	polycystin 1 like 1, transient receptor potential channel interacting
1274	8126954	PKHD1	-2.1272348	-3.7293	-2.86724	0.0467131	polycystic kidney and hepatic disease 1 (autosomal recessive)
1275	8121768	PKIB	-2.502146	-3.1887	-1.73559	0.0216383	protein kinase (cAMP-dependent, catalytic) inhibitor beta
1276	7990151	PKM	-2.1773739	-3.6594	-0.61156	0.0422569	pyruvate kinase, muscle
1277	7948987	PLA2G16	-3.605224	-1.4624	-1.334	0.0018849	phospholipase A2 group XVI
1278	7913226	PLA2G2D	2.2458903	-3.5626	0.852525	0.0367944	phospholipase A2 group IID
1279	7908351	PLA2G4A	2.9807681	-2.4536	1.555519	0.0076794	phospholipase A2 group IVA
1280	8126784	PLA2G7	3.5868212	-1.4917	1.478808	0.0019655	phospholipase A2 group VII
1281	8101429	PLAC8	2.5743418	-3.0804	1.717992	0.0185696	placenta specific 8
1282	7961440	PLBD1	3.1136889	-2.2442	0.971077	0.0057159	phospholipase B domain containing 1
1283	7997453	PLCG2	3.4664342	-1.6835	1.454234	0.0025837	phospholipase C gamma 2
1284	8078187	PLCL2	2.1700792	-3.6697	0.891898	0.0428802	phospholipase C like 2
1285	8111887	PLCXD3	-2.139051	-3.7129	-2.42937	0.045626	phosphatidylinositol specific phospholipase C X domain containing 3
1286	7925511	PLD5	2.1454903	-3.704	0.594502	0.0450434	phospholipase D family member 5
1287	8045247	PLEKHB2	-2.191624	-3.6394	-0.56929	0.0410631	pleckstrin homology domain containing B2
1288	8027398	PLEKHF1	2.4946237	-3.1999	0.725007	0.0219841	pleckstrin homology and FYVE domain containing 1
1289	8147439	PLEKHF2	-2.5239049	-3.1561	-0.85861	0.0206665	pleckstrin homology and FYVE domain containing 2
1290	8122756	PLEKHG1//	3.4938019	-1.6399	1.358957	0.0024281	pleckstrin homology and RhoGEF domain containing G1///pleckstrin
1291	7896798	PLEKHN1	-3.4173396	-1.7618	-0.86401	0.0028876	pleckstrin homology domain containing N1
1292	7905116	PLEKHO1	3.3714435	-1.8349	0.925977	0.0032035	pleckstrin homology domain containing O1
1293	8169061	PLP1	2.3876009	-3.358	0.76457	0.0275003	proteolipid protein 1
1294	8083146	PLS1	-3.356037	-1.8594	-1.52873	0.0033169	plastin 1
1295	8121087	PM20D2	2.287643	-3.5029	0.889371	0.033792	peptidase M20 domain containing 2
1296	8104627	PMCHL1	-2.3984496	-3.3421	-3.43725	0.0268872	pro-melanin concentrating hormone like 1 (pseudogene)
1297	8145603	PNOC	2.4408029	-3.2797	0.452223	0.0246139	prepronociceptin
1298	7937485	PNPLA2	-2.6105624	-3.0257	-0.72755	0.0171895	patatin like phospholipase domain containing 2
1299	8171229	PNPLA4	-2.7735071	-2.7764	-1.06195	0.0120985	patatin like phospholipase domain containing 4
1300	8008064	PNPO	-2.7511301	-2.8109	-0.62627	0.0127007	pyridoxamine 5'-phosphate oxidase
1301	7965343	POC1B	-2.3081096	-3.4735	-0.43792	0.0324044	POC1 centriolar protein B
1302	8173869	POF1B	-2.8134608	-2.7146	-2.6509	0.0110905	premature ovarian failure, 1B
1303	8042953	POLE4	-2.6748647	-2.9279	-0.66763	0.0149755	DNA polymerase epsilon 4, accessory subunit
1304	7945573	POLR2L	-2.6891109	-2.9061	-0.81932	0.014523	RNA polymerase II subunit L
1305	8022428	POTEB3///	-3.2034326	-2.102	-4.5693	0.0046763	POTE ankyrin domain family member B3///POTE ankyrin domain fam
1306	8055222	POTEB3///	-3.1450016	-2.1946	-6.12466	0.0053299	POTE ankyrin domain family member B3///POTE ankyrin domain fam
1307	7977456	POTEB3///	-3.0288979	-2.378	-3.994	0.0069028	POTE ankyrin domain family member B3///POTE ankyrin domain fam
1308	8067844	POTEB3///	-2.7235019	-2.8534	-4.40202	0.0134838	POTE ankyrin domain family member B3///POTE ankyrin domain fam
1309	7986605	POTEB3///	-2.5226477	-3.158	-3.56781	0.0207215	POTE ankyrin domain family member B3///POTE ankyrin domain fam
1310	8074170	POTEB3///	-2.4996509	-3.1924	-3.64427	0.0217524	POTE ankyrin domain family member B3///POTE ankyrin domain fam
1311	8045257	POTEB3///	-2.4742475	-3.2302	-4.13314	0.0229471	POTE ankyrin domain family member B3///POTE ankyrin domain fam
1312	8055153	POTEB3///	-2.4742475	-3.2302	-4.13314	0.0229471	POTE ankyrin domain family member B3///POTE ankyrin domain fam

1313	8045208	POTEF///P(	-2.7610421	-2.7956	-2.6417	0.0124305	POTE ankyrin domain family member F///POTE ankyrin domain fami
1314	8045321	POTEF///P(	-2.2391498	-3.5722	-0.86997	0.0373015	POTE ankyrin domain family member F///POTE ankyrin domain fami
1315	7951596	POU2AF1	3.1181788	-2.2371	0.735221	0.005659	POU class 2 associating factor 1
1316	8108949	POU4F3	-2.774387	-2.7751	-0.51768	0.0120754	POU class 4 homeobox 3
1317	7964577	PP12719	-2.2246844	-3.5927	-0.58693	0.0384116	uncharacterized LOC100653022
1318	8073826	PPARA	3.3680378	-1.8403	0.738252	0.0032282	peroxisome proliferator activated receptor alpha
1319	8100990	PPBPP2	2.1735919	-3.6647	0.959595	0.042579	pro-platelet basic protein pseudogene 2
1320	8064100	PPDPF	-2.1098284	-3.7534	-0.61507	0.0483574	pancreatic progenitor cell differentiation and proliferation factor
1321	8030220	PPFIA3	2.4030295	-3.3353	1.025392	0.0266323	PTPRF interacting protein alpha 3
1322	7904997	PPIAL4C///	-2.4646914	-3.2444	-0.4053	0.0234122	peptidylprolyl isomerase A like 4C///peptidylprolyl isomerase A like 4
1323	7905016	PPIAL4C///	-2.4646914	-3.2444	-0.4053	0.0234122	peptidylprolyl isomerase A like 4C///peptidylprolyl isomerase A like 4
1324	7919436	PPIAL4C///	-2.4646914	-3.2444	-0.4053	0.0234122	peptidylprolyl isomerase A like 4C///peptidylprolyl isomerase A like 4
1325	7919157	PPIAL4C///	-2.4029066	-3.3355	-0.38662	0.0266391	peptidylprolyl isomerase A like 4C///peptidylprolyl isomerase A like 4
1326	7919407	PPIAL4C///	-2.3111008	-3.4692	-0.3955	0.032206	peptidylprolyl isomerase A like 4C///peptidylprolyl isomerase A like 4
1327	8008768	PPM1E	2.3875741	-3.358	0.994193	0.0275018	protein phosphatase, Mg2+/Mn2+ dependent 1E
1328	8083749	PPM1L	2.3498612	-3.413	0.795228	0.0297356	protein phosphatase, Mg2+/Mn2+ dependent 1L
1329	8172197	PPP1R2P9	2.662084	-2.9474	0.571318	0.0153928	protein phosphatase 1 regulatory inhibitor subunit 2 pseudogene 9
1330	7934997	PPP1R3C	-2.1143341	-3.7472	-1.05223	0.0479268	protein phosphatase 1 regulatory subunit 3C
1331	8167476	PPP1R3F	2.3286742	-3.4438	0.494513	0.0310633	protein phosphatase 1 regulatory subunit 3F
1332	8114900	PPP2R2B	2.2241502	-3.5935	1.033841	0.0384531	protein phosphatase 2 regulatory subunit Bbeta
1333	8121257	PRDM1	2.6380182	-2.984	1.113636	0.0162086	PR/SET domain 1
1334	7940996	PRDX5	-2.7088917	-2.8758	-0.71869	0.0139164	peroxiredoxin 5
1335	7934161	PRF1	2.3802666	-3.3687	0.80038	0.027922	perforin 1
1336	7962375	PRICKLE1	2.2255148	-3.5916	0.95962	0.038347	prickle planar cell polarity protein 1
1337	8088550	PRICKLE2	-2.6271559	-3.0005	-0.93119	0.0165902	prickle planar cell polarity protein 2
1338	7901720	PRKAA2	-3.1784634	-2.1416	-1.92763	0.0049454	protein kinase AMP-activated catalytic subunit alpha 2
1339	7994131	PRKCB	2.7603112	-2.7968	1.515094	0.0124502	protein kinase C beta
1340	7931930	PRKCQ	2.4949857	-3.1993	1.420985	0.0219673	protein kinase C theta
1341	8171182	PRKX	2.5342597	-3.1406	1.090036	0.0202186	protein kinase, X-linked
1342	7936590	PRLHR	2.3663848	-3.389	0.864642	0.0287369	prolactin releasing hormone receptor
1343	7903884	PROK1	3.0374842	-2.3645	0.485514	0.0067725	prokineticin 1
1344	8043522	PROM2	-2.4819303	-3.2188	-0.92031	0.0225794	prominin 2
1345	8028016	PROSER3	-2.1161688	-3.7446	-0.65775	0.0477525	proline and serine rich 3
1346	7955250	PRPF40B	-2.567586	-3.0906	-0.7019	0.0188384	pre-mRNA processing factor 40 homolog B
1347	7955810	PRR13	-2.3125748	-3.467	-0.548	0.0321087	proline rich 13
1348	8036808	PRR13	-2.1683352	-3.6721	-0.5223	0.0430304	proline rich 13
1349	8132031	PRR15	-3.0743397	-2.3063	-2.27941	0.0062398	proline rich 15
1350	8016387	PRR15L	-2.229923	-3.5853	-1.15935	0.0380061	proline rich 15 like
1351	8091037	PRR23A///	2.2356252	-3.5772	0.37792	0.0375693	proline rich 23A///proline rich 23B
1352	8095451	PRR27	2.9459963	-2.5082	3.393026	0.0082924	proline rich 27
1353	8033455	PRR36	-3.976284	-0.8745	-0.98817	0.0008083	proline rich 36
1354	8107750	PRRC1	-2.5482724	-3.1196	-0.38879	0.019627	proline rich coiled-coil 1
1355	8030393	PRRG2	-3.5203357	-1.5976	-0.91012	0.0022862	proline rich and Gla domain 2
1356	7939150	PRRG4	-3.8267944	-1.1105	-1.21724	0.0011373	proline rich and Gla domain 4
1357	8136807	PRSS3P2//,	-2.9201365	-2.5486	-2.85581	0.0087786	protease, serine 3 pseudogene 2///protease, serine 2///protease, ser
1358	8161884	PRUNE2	2.1044553	-3.7608	0.792791	0.0488755	prune homolog 2
1359	8054769	PS1TP4///S	-2.691253	-2.9028	-0.78363	0.0144562	HBV preS1-transactivated protein 4///solute carrier family 35 membe
1360	8037246	PSG6	2.7986671	-2.7375	0.567701	0.011454	pregnancy specific beta-1-glycoprotein 6
1361	8088535	PSMD6	-3.2341936	-2.0532	-0.72564	0.0043644	proteasome 26S subunit, non-ATPase 6
1362	7959322	PSMD9	-3.2363525	-2.0498	-0.76574	0.0043433	proteasome 26S subunit, non-ATPase 9
1363	8118076	PSORS1C1	-3.0261127	-2.3824	-0.77909	0.0069456	psoriasis susceptibility 1 candidate 1
1364	8177938	PSORS1C1	-3.0261127	-2.3824	-0.77909	0.0069456	psoriasis susceptibility 1 candidate 1
1365	8106107	PTCD2	-3.0692749	-2.3143	-0.57742	0.0063105	pentatricopeptide repeat domain 2
1366	8162533	PTCH1	2.6210123	-3.0098	1.31628	0.0168097	patched 1
1367	8166447	PTCHD1	4.1330716	-0.6288	2.281558	0.000565	patched domain containing 1
1368	7926356	PTER	-2.8479085	-2.6612	-0.71319	0.0102865	phosphotriesterase related
1369	8159521	PTGDS	2.6910529	-2.9031	1.417103	0.0144624	prostaglandin D2 synthase
1370	7964250	PTGES3	-2.3531231	-3.4083	-0.46612	0.0295359	prostaglandin E synthase 3
1371	7922976	PTGS2	3.0528234	-2.3403	3.597509	0.0065456	prostaglandin-endoperoxide synthase 2
1372	8047910	PTH2R	2.1895227	-3.6424	0.950149	0.0412372	parathyroid hormone 2 receptor
1373	8145490	PTK2B	2.7734374	-2.7765	0.833227	0.0121003	protein tyrosine kinase 2 beta
1374	8067662	PTK6	-2.1952259	-3.6344	-0.93235	0.0407663	protein tyrosine kinase 6
1375	8119689	PTK7	2.2339883	-3.5795	0.965811	0.0376942	protein tyrosine kinase 7 (inactive)
1376	7918657	PTPN22	2.2384623	-3.5732	1.10899	0.0373536	protein tyrosine phosphatase, non-receptor type 22
1377	8163149	PTPN3	-2.8790748	-2.6127	-0.78102	0.0096075	protein tyrosine phosphatase, non-receptor type 3
1378	7908553	PTPRC	2.1036723	-3.7619	1.059771	0.0489514	protein tyrosine phosphatase, receptor type C
1379	8001030	PYCARD	-3.3234369	-1.9113	-1.20133	0.0035702	PYD and CARD domain containing
1380	7906386	PYHIN1	2.2182783	-3.6018	1.311132	0.0389128	pyrin and HIN domain family member 1
1381	7963965	PYM1	-2.434101	-3.2896	-0.54976	0.0249612	PYM homolog 1, exon junction complex associated factor
1382	8099541	QDPR	-2.4378163	-3.2841	-0.95963	0.0247681	quinoid dihydropteridine reductase
1383	8116722	QIQN5815	2.6510646	-2.9641	0.471113	0.0157614	uncharacterized LOC100129033
1384	8041508	QPCT	2.8193175	-2.7056	1.329513	0.0109497	glutaminyl-peptide cyclotransferase
1385	7907830	QSOX1	2.1574574	-3.6873	0.988865	0.0439784	quiescin sulfhydryl oxidase 1

1386	8165094	QSOX2	2.3955476	-3.3463	0.880612	0.02705	quiescin sulfhydryl oxidase 2
1387	7984289	RAB11A	-3.7493494	-1.2333	-0.57632	0.0013572	RAB11A, member RAS oncogene family
1388	8052994	RAB11FIP5	-2.3949324	-3.3472	-0.44945	0.0270846	RAB11 family interacting protein 5
1389	7963986	RAB13	-2.6342982	-2.9896	-0.52538	0.0163384	RAB13, member RAS oncogene family
1390	7979663	RAB15	2.1775359	-3.6592	1.045211	0.0422431	RAB15, member RAS oncogene family
1391	8136580	RAB19	-3.1320997	-2.2151	-0.82672	0.0054858	RAB19, member RAS oncogene family
1392	7992518	RAB26	-2.7726964	-2.7777	-1.33226	0.0121198	RAB26, member RAS oncogene family
1393	8021301	RAB27B	-2.8543596	-2.6512	-1.74813	0.0101423	RAB27B, member RAS oncogene family
1394	8099395	RAB28	-2.4750816	-3.2289	-0.53921	0.0229069	RAB28, member RAS oncogene family
1395	8009666	RAB37	2.9871264	-2.4437	0.70374	0.0075722	RAB37, member RAS oncogene family
1396	7950899	RAB38	-2.2986285	-3.4871	-1.55893	0.0330405	RAB38, member RAS oncogene family
1397	8176230	RAB39B	3.3481034	-1.872	1.001832	0.0033769	RAB39B, member RAS oncogene family
1398	7957072	RAB3IP	-2.5511794	-3.1152	-1.33818	0.0195064	RAB3A interacting protein
1399	8169006	RAB40AL	-2.3159794	-3.4621	-0.58431	0.031885	RAB40A, member RAS oncogene family-like
1400	7956088	RAB5B	-2.4916646	-3.2043	-0.54491	0.0221216	RAB5B, member RAS oncogene family
1401	8015545	RAB5C	-2.1069384	-3.7574	-0.42562	0.0486355	RAB5C, member RAS oncogene family
1402	8180340	RAB7B	4.1530092	-0.5977	1.033745	0.0005399	RAB7B, member RAS oncogene family
1403	7907492	RABGAP1L	2.4632794	-3.2465	0.886191	0.0234817	RAB GTPase activating protein 1 like
1404	8089867	RABL3	-3.6999193	-1.3118	-0.75447	0.0015192	RAB, member of RAS oncogene family like 3
1405	8131406	RAC1	-2.8764051	-2.6168	-0.54261	0.009664	ras-related C3 botulinum toxin substrate 1 (rho family, small GTP bir
1406	8107942	RAD50	-2.9621338	-2.4829	-0.54025	0.0080022	RAD50 double strand break repair protein
1407	8008754	RAD51C	-2.257102	-3.5466	-0.61955	0.035965	RAD51 paralog C
1408	7907657	RALGPS2	-3.5686646	-1.5206	-1.13685	0.0020484	Ral GEF with PH domain and SH3 binding motif 2
1409	8164692	RAPGEF1	2.5794406	-3.0727	1.026222	0.0183692	Rap guanine nucleotide exchange factor 1
1410	8007084	RARA///BC	-2.9634885	-2.4807	-0.92036	0.0079783	retinoic acid receptor alpha///BCL6 corepressor
1411	7963631	RARG///RA	-3.0907773	-2.2804	-0.91428	0.0060155	retinoic acid receptor gamma///retinoic acid receptor gamma
1412	8109802	RARS	-3.2163092	-2.0816	-0.64829	0.0045432	arginyl-tRNA synthetase
1413	7972946	RASA3	2.2463135	-3.562	0.71559	0.0367628	RAS p21 protein activator 3
1414	8035050	RASAL3	2.7854985	-2.7579	0.978177	0.0117871	RAS protein activator like 3
1415	8161945	RASEF	-3.6455758	-1.3982	-1.41002	0.0017195	RAS and EF-hand domain containing
1416	8116384	RASGEF1C	3.018938	-2.3937	0.665577	0.007057	RasGEF domain family member 1C
1417	7949104	RASGRP2	2.9769022	-2.4597	2.330201	0.0077453	RAS guanyl releasing protein 2
1418	8041422	RASGRP3	2.3280058	-3.4447	0.771079	0.0311061	RAS guanyl releasing protein 3
1419	8075211	RASL10A	2.2313783	-3.5832	0.474595	0.0378942	RAS like family 10 member A
1420	7909214	RASSF5	2.2017681	-3.6251	0.86948	0.0402322	Ras association domain family member 5
1421	7902023	RAVER2	2.3581717	-3.4009	1.0311	0.0292293	ribonucleoprotein, PTB binding 2
1422	8023549	RAX	2.4867801	-3.2116	0.520342	0.0223502	retina and anterior neural fold homeobox
1423	8060353	RBCK1	-3.3916381	-1.8027	-0.97118	0.0030605	RANBP2-type and C3HC4-type zinc finger containing 1
1424	8163948	RBM18	-2.3374823	-3.431	-0.59761	0.0305048	RNA binding motif protein 18
1425	8117045	RBM24	-2.7226706	-2.8547	-3.61665	0.0135081	RNA binding motif protein 24
1426	8063583	RBM38	3.7032433	-1.3065	0.764161	0.0015077	RNA binding motif protein 38
1427	8046628	RBM45	-2.3497618	-3.4132	-0.44993	0.0297417	RNA binding motif protein 45
1428	8169463	RBMXL3	2.608262	-3.0291	1.218281	0.0172742	RNA binding motif protein, X-linked like 3
1429	8073334	RBX1	-2.5538635	-3.1112	-0.56141	0.0193956	ring-box 1
1430	8101013	RCHY1	-2.096291	-3.772	-0.64346	0.0496723	ring finger and CHY zinc finger domain containing 1
1431	7949021	RCOR2	2.5955664	-3.0483	1.870953	0.0177487	REST corepressor 2
1432	7909529	RCOR3	-2.1007857	-3.7658	-0.53197	0.0492322	REST corepressor 3
1433	7907079	RCSD1	4.2426026	-0.4585	1.654244	0.00044	RCSD domain containing 1
1434	8053551	REEP1	-2.1822023	-3.6527	-1.21475	0.0418489	receptor accessory protein 1
1435	7927799	REEP3	-2.4476512	-3.2696	-0.65928	0.0242636	receptor accessory protein 3
1436	8053337	REG1CP	-2.1330887	-3.7212	-2.87829	0.0461717	regenerating family member 1 gamma, pseudogene
1437	8166243	REPS2	-2.2452747	-3.5635	-1.20052	0.0368405	RALBP1 associated Eps domain containing 2
1438	7961595	RERGL	2.3269736	-3.4462	1.896016	0.0311723	RERG like
1439	8095262	REST	-2.2837304	-3.5085	-0.45483	0.0340635	RE1 silencing transcription factor
1440	7927120	RET///RET/	-2.6271984	-3.0004	-4.24049	0.0165887	ret proto-oncogene///ret proto-oncogene///kinesin family member !
1441	8054227	REV1	-2.2749407	-3.5211	-0.43806	0.0346806	REV1, DNA directed polymerase
1442	7968789	RGCC	2.284159	-3.5079	0.916026	0.0340337	regulator of cell cycle
1443	7991459	RGMA	2.6295144	-2.9969	0.751962	0.0165066	repulsive guidance molecule family member a
1444	8107100	RGMB	-2.3495568	-3.4135	-0.60838	0.0297543	repulsive guidance molecule family member b
1445	8155110	RGP1	-2.3013595	-3.4832	-0.39936	0.0328561	RGP1 homolog, RAB6A GEF complex partner 1
1446	7908388	RGS1	2.1978142	-3.6307	0.932697	0.0405543	regulator of G-protein signaling 1
1447	7908409	RGS2	3.5981973	-1.4736	1.57169	0.0019153	regulator of G-protein signaling 2
1448	7908382	RGS21	2.8055513	-2.7269	1.297533	0.0112834	regulator of G-protein signaling 21
1449	8126878	RHAG	3.5304624	-1.5815	0.826722	0.0022342	Rh-associated glycoprotein
1450	8087409	RHOA	-2.4444782	-3.2743	-0.37191	0.0244253	ras homolog family member A
1451	8040473	RHOB	-2.5923626	-3.0532	-0.84932	0.0178704	ras homolog family member B
1452	7967202	RHOF	2.938242	-2.5203	1.05693	0.0084354	ras homolog family member F, filopodia associated
1453	8035980	RHPN2	-2.7386377	-2.8301	-1.13934	0.0130493	rhophilin Rho GTPase binding protein 2
1454	8073766	RIBC2	-3.3610642	-1.8514	-1.85915	0.0032795	RIB43A domain with coiled-coils 2
1455	7967456	RILPL2	-2.2644853	-3.5361	-0.55599	0.0354282	Rab interacting lysosomal protein like 2
1456	8074771	RIMBP3B//	2.5961308	-3.0475	0.582028	0.0177273	RIMS binding protein 3B///RIMS binding protein 3C///RIMS binding
1457	8074593	RIMBP3B//	2.3440652	-3.4214	0.568687	0.0300935	RIMS binding protein 3B///RIMS binding protein 3C///RIMS binding
1458	7900540	RIMKLA	-2.6498944	-2.9659	-2.53088	0.015801	ribosomal modification protein rimK like family member A

1459	7987536	RMDN3	-2.1167762	-3.7438	-0.41429	0.0476949	regulator of microtubule dynamics 3
1460	8130191	RMND1	-2.6074873	-3.0303	-1.0669	0.0173028	required for meiotic nuclear division 1 homolog
1461	8110437	RMND5B	-4.3145854	-0.3473	-0.89289	0.0003735	required for meiotic nuclear division 5 homolog B
1462	7962884	RND1	-2.2951336	-3.4922	-1.94409	0.0332779	Rho family GTPase 1
1463	8053576	RNF103	-2.9684692	-2.4729	-0.7247	0.007891	ring finger protein 103
1464	8053562	RNF103-CI	-2.2840804	-3.508	-0.37535	0.0340391	RNF103-CHMP3 readthrough///charged multivesicular body protein
1465	8150186	RNF122	2.6685588	-2.9375	0.718234	0.01518	ring finger protein 122
1466	8169174	RNF128	-2.7853978	-2.758	-2.09903	0.0117897	ring finger protein 128, E3 ubiquitin protein ligase
1467	8040090	RNF144A	2.9308182	-2.5319	1.018399	0.0085745	ring finger protein 144A
1468	8043209	RNF181	-2.2523346	-3.5534	-0.5112	0.0363155	ring finger protein 181
1469	7964021	RNF41	-3.0514125	-2.3425	-0.63443	0.0065662	ring finger protein 41
1470	7945420	RNH1	-2.6850982	-2.9122	-0.58676	0.0146492	ribonuclease/angiogenin inhibitor 1
1471	7908779	RNPEP	-3.2093877	-2.0926	-0.69905	0.0046143	arginyl aminopeptidase
1472	8081001	ROBO2	-2.7048774	-2.8819	-3.73154	0.0140375	roundabout guidance receptor 2
1473	7901969	ROR1	2.5851904	-3.064	1.255125	0.0181456	receptor tyrosine kinase like orphan receptor 1
1474	7920082	RORC	-3.3982136	-1.7922	-1.71363	0.0030153	RAR related orphan receptor C
1475	8168723	RPA4	2.4907623	-3.2056	0.654213	0.0221636	replication protein A4
1476	7958860	RPH3A	2.5378106	-3.1353	0.601567	0.0200671	rabphilin 3A
1477	7936320	RPL13AP6	2.1829362	-3.6516	0.404559	0.0417872	ribosomal protein L13a pseudogene 6
1478	8092067	RPL22L1	2.2549066	-3.5498	0.630163	0.036126	ribosomal protein L22 like 1
1479	8127526	RPL39P5//,	2.1828685	-3.6517	0.492895	0.0417929	ribosomal protein L39 pseudogene 5///zinc finger protein 525///ribc
1480	8174710	RPL39P5//,	2.1828685	-3.6517	0.492895	0.0417929	ribosomal protein L39 pseudogene 5///zinc finger protein 525///ribc
1481	8022914	RPRD1A	-3.1870933	-2.128	-0.59675	0.0048508	regulation of nuclear pre-mRNA domain containing 1A
1482	7908692	RPS10P7	-2.3955921	-3.3463	-0.48233	0.0270474	ribosomal protein S10 pseudogene 7
1483	8118644	RPS18P9//,	2.319442	-3.4571	0.462254	0.0316589	ribosomal protein S18 pseudogene 9///ribosomal protein S18
1484	8178253	RPS18P9//,	2.319442	-3.4571	0.462254	0.0316589	ribosomal protein S18 pseudogene 9///ribosomal protein S18
1485	8179544	RPS18P9//,	2.319442	-3.4571	0.462254	0.0316589	ribosomal protein S18 pseudogene 9///ribosomal protein S18
1486	7909661	RPS6KC1	-2.2256168	-3.5914	-0.48192	0.0383391	ribosomal protein S6 kinase C1
1487	8078905	RPSAP36//	2.1882333	-3.6442	0.539654	0.0413444	ribosomal protein SA pseudogene 36///ribosomal protein SA pseudo
1488	8027354	RPSAP58	-2.4310594	-3.2941	-0.80372	0.0251204	ribosomal protein SA pseudogene 58
1489	8038407	RRAS	-3.0579812	-2.3322	-0.88373	0.006471	related RAS viral (r-ras) oncogene homolog
1490	7906223	RRNAD1	-3.0344539	-2.3692	-0.61006	0.0068182	ribosomal RNA adenine dimethylase domain containing 1
1491	8130528	RSPH3	-2.4168145	-3.3151	-0.51741	0.0258784	radial spoke 3 homolog
1492	8052204	RTN4	-2.2072429	-3.6174	-0.45158	0.0397902	reticulon 4
1493	8011214	RTN4RL1	-2.3290483	-3.4432	-1.17498	0.0310394	reticulon 4 receptor like 1
1494	8049952	RTP5	2.1091515	-3.7543	0.38627	0.0484224	receptor transporter protein 5 (putative)
1495	8007435	RUNDC1	-2.3637162	-3.3928	-0.54201	0.028896	RUN domain containing 1
1496	8007607	RUNDC3A	2.329719	-3.4422	0.519455	0.0309966	RUN domain containing 3A
1497	8121613	RWDD1	-2.8377037	-2.677	-0.56921	0.0105186	RWD domain containing 1
1498	8088820	RYBP	-2.2110068	-3.6121	-0.47825	0.0394889	RING1 and YY1 binding protein
1499	7982392	RYR3	2.1481482	-3.7003	1.607494	0.0448048	ryanodine receptor 3
1500	7920303	S100A13	-2.6494818	-2.9666	-1.00606	0.015815	S100 calcium binding protein A13
1501	7920297	S100A14	-3.8117144	-1.1344	-2.43194	0.0011772	S100 calcium binding protein A14
1502	7920291	S100A16	-2.4523788	-3.2626	-1.26473	0.0240246	S100 calcium binding protein A16
1503	7903393	S1PR1	2.3117248	-3.4683	1.016754	0.0321648	sphingosine-1-phosphate receptor 1
1504	8067792	SAMD10	2.8559231	-2.6487	0.614511	0.0101076	sterile alpha motif domain containing 10
1505	8152506	SAMD12	-2.1698841	-3.6699	-0.91409	0.0428969	sterile alpha motif domain containing 12
1506	8129482	SAMD3	2.2140274	-3.6078	1.088794	0.0392486	sterile alpha motif domain containing 3
1507	7967976	SAP18	-2.8427289	-2.6692	-0.49325	0.0104037	Sin3A associated protein 18
1508	8169859	SASH3	2.8663389	-2.6325	0.826544	0.0098796	SAM and SH3 domain containing 3
1509	8085716	SATB1	2.307504	-3.4743	1.425171	0.0324447	SATB homeobox 1
1510	8106479	SCAMP1	-2.1164867	-3.7442	-0.43706	0.0477224	secretory carrier membrane protein 1
1511	8021181	SCARNA17	2.3593475	-3.3992	0.408728	0.0291584	small Cajal body-specific RNA 17
1512	7911155	SCCPDH	-3.4200768	-1.7574	-1.33559	0.0028698	saccharopine dehydrogenase (putative)
1513	7981718	SCFV///IGH	2.223743	-3.5941	1.706866	0.0384848	single-chain Fv fragment///immunoglobulin heavy constant mu///sin
1514	7981740	SCFV///IGL	2.1357696	-3.7175	1.763732	0.0459256	single-chain Fv fragment///immunoglobulin lambda joining 3///imm
1515	7981724	SCFV///IGL	2.2706227	-3.5273	1.76642	0.0349876	single-chain Fv fragment///immunoglobulin lambda joining 3///imm
1516	7995263	SCFV///IGL	2.1662799	-3.675	1.73316	0.0432081	single-chain Fv fragment///immunoglobulin lambda joining 3///imm
1517	8059345	SCG2	2.4670928	-3.2408	0.898211	0.0232945	secretogranin II
1518	8131550	SCIN	-2.5688768	-3.0886	-1.878	0.0187867	scinderin
1519	8128638	SCML4	2.1066506	-3.7578	0.984274	0.0486632	sex comb on midleg-like 4 (Drosophila)
1520	8046020	SCN2A	2.1031629	-3.7626	1.541865	0.0490009	sodium voltage-gated channel alpha subunit 2
1521	7955533	SCN8A	-2.2369942	-3.5753	-1.49752	0.0374651	sodium voltage-gated channel alpha subunit 8
1522	7960529	SCNN1A	-4.3374064	-0.3121	-2.24939	0.0003545	sodium channel epithelial 1 alpha subunit
1523	8097521	SCOC	-2.8183145	-2.7071	-0.75131	0.0109737	short coiled-coil protein
1524	8046502	SCRN3	-3.0465091	-2.3502	-0.67305	0.0066381	secernin 3
1525	8148796	SCX	2.1548988	-3.6909	0.555642	0.0442042	scleraxis bHLH transcription factor
1526	8148821	SCX	2.1548988	-3.6909	0.555642	0.0442042	scleraxis bHLH transcription factor
1527	8071559	SDF2L1	2.8179013	-2.7078	0.516445	0.0109836	stromal cell derived factor 2 like 1
1528	8134415	SDHAF3	-2.2194826	-3.6001	-0.94109	0.0388181	succinate dehydrogenase complex assembly factor 3
1529	7912928	SDHB	-2.4164502	-3.3156	-0.60323	0.0258981	succinate dehydrogenase complex iron sulfur subunit B
1530	8011212	SDHC	-2.1593969	-3.6846	-0.44044	0.043808	succinate dehydrogenase complex subunit C
1531	8150889	SDR16C5	-3.7628149	-1.2119	-4.91753	0.0013162	short chain dehydrogenase/reductase family 16C, member 5

1532	7964285	SDR9C7	2.1933872	-3.6369	0.673367	0.0409176	short chain dehydrogenase/reductase family 9C, member 7
1533	8021453	SEC11C	2.2880618	-3.5023	0.775829	0.0337631	SEC11 homolog C, signal peptidase complex subunit
1534	8099721	SEL1L3	2.5760233	-3.0778	1.24023	0.0185033	SEL1L family member 3
1535	7922229	SELE	3.2231586	-2.0707	1.329174	0.0044739	selectin E
1536	7922219	SELL	3.4424907	-1.7217	2.016261	0.0027277	selectin L
1537	8079966	SEMA3B	-2.7789546	-2.768	-2.00552	0.0119561	semaphorin 3B
1538	8140534	SEMA3C	-4.2576623	-0.4352	-1.60749	0.0004252	semaphorin 3C
1539	8140650	SEMA3E	-3.5007605	-1.6288	-1.86655	0.0023901	semaphorin 3E
1540	8079896	SEMA3F	-2.5259205	-3.1531	-1.12476	0.0205786	semaphorin 3F
1541	7984626	SENP8	-2.2922652	-3.4963	-0.62674	0.0334739	SUMO/sentrin peptidase family member, NEDD8 specific
1542	8000869	SEPT1	2.8674736	-2.6308	1.258511	0.0098551	septin 1
1543	8054467	SEPT10	-3.0214532	-2.3897	-0.7379	0.0070178	septin 10
1544	8049827	SEPT2	-2.4377541	-3.2842	-0.39169	0.0247713	septin 2
1545	8174692	SEPT6	2.7480786	-2.8156	1.005141	0.012785	septin 6
1546	8129317	SERINC1	-2.201682	-3.6253	-0.55809	0.0402392	serine incorporator 1
1547	7899615	SERINC2	-2.3973164	-3.3437	-1.02247	0.0269507	serine incorporator 2
1548	8123621	SERPINB6	-3.5671166	-1.5231	-0.75101	0.0020556	serpin family B member 6
1549	8123609	SERPINB9	2.1526992	-3.6939	0.807461	0.0443991	serpin family B member 9
1550	8071420	SERPIND1	2.7068273	-2.879	0.59831	0.0139785	serpin family D member 1
1551	7931951	SFMBT2	2.4004965	-3.3391	1.022892	0.026773	Scm-like with four mbt domains 2
1552	7899265	SFN	-3.1700082	-2.155	-1.76619	0.00504	stratifin
1553	8150428	SFRP1	2.6291151	-2.9975	2.784663	0.0165207	secreted frizzled related protein 1
1554	7935528	SFRP5	2.6532153	-2.9609	0.537484	0.0156888	secreted frizzled related protein 5
1555	7907135	SFT2D2	2.9658304	-2.4771	0.84328	0.0079372	SFT2 domain containing 2
1556	8129677	SGK1	2.2170739	-3.6035	0.76625	0.0390077	serum/glucocorticoid regulated kinase 1
1557	7928171	SGPL1	-2.0976897	-3.7701	-0.44816	0.049535	sphingosine-1-phosphate lyase 1
1558	8135149	SH2B2	2.3250826	-3.4489	1.230318	0.0312938	SH2B adaptor protein 2
1559	8169792	SH2D1A	2.741732	-2.8254	1.763305	0.0129621	SH2 domain containing 1A
1560	7921144	SH2D2A	2.5021833	-3.1886	0.560314	0.0216366	SH2 domain containing 2A
1561	8164252	SH2D3C	2.1683251	-3.6721	0.790999	0.0430313	SH2 domain containing 3C
1562	8072847	SH3BP1	3.8763114	-1.0322	1.291875	0.0010157	SH3 domain binding protein 1
1563	8085556	SH3BP5	2.5507477	-3.1159	0.967916	0.0195242	SH3 domain binding protein 5
1564	8171684	SH3KBP1	2.2342086	-3.5792	0.912899	0.0376774	SH3 domain containing kinase binding protein 1
1565	7988563	SHC4	2.4221728	-3.3072	1.69665	0.0255908	SHC adaptor protein 4
1566	8114068	SHROOM1	-2.7574811	-2.8011	-0.82238	0.0125269	shroom family member 1
1567	8095834	SHROOM3	-2.1760002	-3.6614	-0.85781	0.0423736	shroom family member 3
1568	8030563	SIGLEC16	-3.6627958	-1.3708	-0.68381	0.0016533	sialic acid binding Ig like lectin 16 (gene/pseudogene)
1569	7990487	SIN3A	-2.9958947	-2.4299	-0.61878	0.0074266	SIN3 transcription regulator family member A
1570	8064485	SIRPG	2.2993145	-3.4861	0.99961	0.0329941	signal regulatory protein gamma
1571	8032770	SIRT6	-2.478924	-3.2232	-0.5242	0.0227226	sirtuin 6
1572	8161017	SIT1	2.7138466	-2.8682	1.161844	0.0137682	signaling threshold regulating transmembrane adaptor 1
1573	8180312	SKP1	-2.5025987	-3.188	-0.52443	0.0216176	S-phase kinase-associated protein 1
1574	8152988	SLA	2.119974	-3.7394	0.875039	0.0473928	Src-like-adaptor
1575	8066038	SLA2	2.2434516	-3.5661	1.051698	0.0369772	Src like adaptor 2
1576	7921652	SLAMF1	2.3882894	-3.357	1.470424	0.027461	signaling lymphocytic activation molecule family member 1
1577	7921625	SLAMF6	2.1071438	-3.7571	1.097321	0.0486157	SLAM family member 6
1578	7906613	SLAMF7	2.1913215	-3.6398	1.304383	0.0410882	SLAM family member 7
1579	8151559	SLC10A5	-3.6298472	-1.4232	-1.39724	0.0017822	solute carrier family 10 member 5
1580	7963212	SLC11A2	-2.9719891	-2.4674	-0.67491	0.0078299	solute carrier family 11 member 2
1581	8142628	SLC13A1	2.1286296	-3.7274	0.381936	0.0465836	solute carrier family 13 member 1
1582	8009746	SLC16A5	-2.8420101	-2.6703	-1.08806	0.0104201	solute carrier family 16 member 5
1583	8017843	SLC16A6	-2.094879	-3.774	-1.4421	0.0498113	solute carrier family 16 member 6
1584	8154135	SLC1A1	-2.8705575	-2.626	-1.81638	0.0097887	solute carrier family 1 member 1
1585	7904226	SLC22A15	-2.2332518	-3.5806	-0.89702	0.0377505	solute carrier family 22 member 15
1586	7937852	SLC22A18	-2.0952049	-3.7735	-0.77915	0.0497792	solute carrier family 22 member 18
1587	8123658	SLC22A23	-2.3495223	-3.4135	-0.8536	0.0297564	solute carrier family 22 member 23
1588	8071107	SLC25A18	2.2501216	-3.5566	1.266692	0.0364794	solute carrier family 25 member 18
1589	7918188	SLC25A24	-3.2600777	-2.0121	-0.76926	0.0041177	solute carrier family 25 member 24
1590	8120460	SLC25A51I	-2.3317755	-3.4393	-1.00846	0.0308656	solute carrier family 25 member 51 pseudogene 1
1591	8174288	SLC25A53	2.3959757	-3.3457	0.381234	0.0270259	solute carrier family 25 member 53
1592	8174675	SLC25A5-A	2.2809494	-3.5125	0.602229	0.0342576	SLC25A5 antisense RNA 1
1593	7956573	SLC26A10	3.4112828	-1.7714	0.923417	0.0029275	solute carrier family 26 member 10
1594	8141922	SLC26A5	-2.1181324	-3.7419	-3.14886	0.0475666	solute carrier family 26 member 5
1595	7983650	SLC27A2	-3.4209121	-1.7561	-2.87495	0.0028643	solute carrier family 27 member 2
1596	8063177	SLC2A10	-2.8824505	-2.6074	-1.30765	0.0095366	solute carrier family 2 member 10
1597	7960850	SLC2A14	3.149104	-2.1882	0.761133	0.0052812	solute carrier family 2 member 14
1598	7960865	SLC2A3	2.8999287	-2.5802	0.836512	0.0091775	solute carrier family 2 member 3
1599	7924342	SLC30A10	2.5580713	-3.1049	0.449697	0.0192231	solute carrier family 30 member 10
1600	8105801	SLC30A5	-2.6947457	-2.8975	-0.50467	0.0143477	solute carrier family 30 member 5
1601	7903281	SLC35A3	-2.5489884	-3.1185	-0.67878	0.0195972	solute carrier family 35 member A3
1602	8054771	SLC35F5	-2.2565533	-3.5474	-0.70427	0.0360052	solute carrier family 35 member F5
1603	7962537	SLC38A2	-2.5851218	-3.0641	-0.63937	0.0181483	solute carrier family 38 member 2
1604	8022927	SLC39A6	-2.6486744	-2.9678	-1.63293	0.0158424	solute carrier family 39 member 6

1605	7975344	SLC39A9	-2.2636972	-3.5372	-0.65099	0.0354851	solute carrier family 39 member 9
1606	8157038	SLC44A1	-2.5282749	-3.1496	-0.68769	0.0204763	solute carrier family 44 member 1
1607	8125149	SLC44A4	-4.0000128	-0.8372	-3.24691	0.0007657	solute carrier family 44 member 4
1608	8178653	SLC44A4	-4.0000128	-0.8372	-3.24691	0.0007657	solute carrier family 44 member 4
1609	8179861	SLC44A4	-4.0000128	-0.8372	-3.24691	0.0007657	solute carrier family 44 member 4
1610	7917052	SLC44A5	-3.181235	-2.1372	-2.12546	0.0049148	solute carrier family 44 member 5
1611	8104281	SLC6A18	2.4957208	-3.1982	0.449807	0.0219333	solute carrier family 6 member 18
1612	8086540	SLC6A20	2.2629108	-3.5383	0.555307	0.035542	solute carrier family 6 member 20
1613	8151637	SLC7A13	-2.4516272	-3.2637	-3.20339	0.0240624	solute carrier family 7 member 13
1614	7977933	SLC7A8	-2.1672617	-3.6736	-1.2809	0.0431231	solute carrier family 7 member 8
1615	8009685	SLC9A3R1	-2.1289697	-3.7269	-0.96791	0.0465521	SLC9A3 regulator 1
1616	8151223	SLCO5A1	3.5564859	-1.54	1.902395	0.0021059	solute carrier organic anion transporter family member 5A1
1617	7969569	SLITRK5	2.7201082	-2.8586	1.501067	0.0135831	SLIT and NTRK like family member 5
1618	8080685	SLMAP	-2.292467	-3.496	-0.36601	0.0334601	sarcolemma associated protein
1619	8174985	SMARCA1	-2.3690568	-3.3851	-0.88714	0.0285784	SWI/SNF related, matrix associated, actin dependent regulator of chı
1620	8050443	SMC6	2.1303535	-3.725	0.581978	0.046424	structural maintenance of chromosomes 6
1621	8019877	SMCHD1	2.6243607	-3.0047	0.558404	0.0166897	structural maintenance of chromosomes flexible hinge domain conta
1622	8099912	SMIM14	-3.1541425	-2.1802	-1.48677	0.0052221	small integral membrane protein 14
1623	8112302	SMIM15	-2.4555058	-3.258	-0.40076	0.0238676	small integral membrane protein 15
1624	8136080	SMO	2.4953552	-3.1988	0.776403	0.0219502	smoothened, frizzled class receptor
1625	7975390	SMOC1	2.5152369	-3.1691	0.942074	0.0210486	SPARC related modular calcium binding 1
1626	7963741	SMUG1	-3.8965577	-1.0002	-0.93827	0.0009698	single-strand-selective monofunctional uracil-DNA glycosylase 1
1627	7994926	SNORA30	-2.1988307	-3.6293	-0.52288	0.0404712	small nucleolar RNA, H/ACA box 30
1628	8078918	SNORA62	2.2040143	-3.622	0.91383	0.0400504	small nucleolar RNA, H/ACA box 62
1629	8090565	SNORA7B	2.3295527	-3.4425	0.45265	0.0310072	small nucleolar RNA, H/ACA box 7B
1630	7982014	SNORD115	2.2341954	-3.5792	3.594201	0.0376784	small nucleolar RNA, C/D box 115-4
1631	8146403	SNTG1	2.5845679	-3.065	1.204408	0.0181697	syntrophin gamma 1
1632	7984174	SNX22	2.787554	-2.7547	0.824585	0.0117345	sorting nexin 22
1633	7993825	SNX29P1//	2.3688937	-3.3853	0.727927	0.028588	sorting nexin 29 pseudogene 1///sorting nexin 29 pseudogene 2///s
1634	7994576	SNX29P1//	2.2317275	-3.5828	0.671809	0.0378674	sorting nexin 29 pseudogene 1///sorting nexin 29 pseudogene 2///s
1635	7903203	SNX7	-4.1465416	-0.6078	-1.13292	0.0005479	sorting nexin 7
1636	7907702	SOAT1	2.9076863	-2.5681	0.958192	0.0090224	sterol O-acyltransferase 1
1637	8121319	SOBP	2.1874815	-3.6452	2.203522	0.0414069	sine oculis binding protein homolog
1638	8021707	SOCS6	-2.8651286	-2.6344	-0.65006	0.0099059	suppressor of cytokine signaling 6
1639	8165038	SOHLH1	2.0967538	-3.7714	0.416851	0.0496269	spermatogenesis and oogenesis specific basic helix-loop-helix 1
1640	8169657	SOWAHD	2.2082794	-3.6159	0.544286	0.039707	sosondowah ankyrin repeat domain family member D
1641	7970146	SOX1	2.1370485	-3.7157	0.392042	0.0458086	SRY-box 1
1642	8075992	SOX10	2.7794909	-2.7672	2.25882	0.0119421	SRY-box 10
1643	8146462	SOX17	2.7054846	-2.881	0.541321	0.0140191	SRY-box 17
1644	8048898	SP140	2.2290891	-3.5865	1.219554	0.0380704	SP140 nuclear body protein
1645	8056798	SP3	-2.2843807	-3.5076	-0.3784	0.0340182	Sp3 transcription factor
1646	7944869	SPA17	-2.6222323	-3.008	-0.61675	0.0167659	sperm autoantigenic protein 17
1647	7918973	SPAG17	-3.6239199	-1.4327	-4.6838	0.0018064	sperm associated antigen 17
1648	8135835	SPAM1	-3.0221344	-2.3886	-1.4654	0.0070072	sperm adhesion molecule 1
1649	7909768	SPATA17	-3.3262182	-1.9069	-1.84144	0.0035478	spermatogenesis associated 17
1650	8008388	SPATA20	-2.9739497	-2.4643	-0.92871	0.0077961	spermatogenesis associated 20
1651	8134785	SPDYE3	2.2792481	-3.515	0.406975	0.0343769	speedy/RINGO cell cycle regulator family member E3
1652	8010061	SPHK1	3.8788993	-1.0281	0.864488	0.0010097	sphingosine kinase 1
1653	8030671	SPIB	3.8819947	-1.0232	2.095019	0.0010026	Spi-B transcription factor
1654	8089627	SPICE1	-2.7008534	-2.8881	-0.699	0.0141599	spindle and centriole associated protein 1
1655	8156263	SPIN1	-2.1226135	-3.7357	-0.45475	0.0471447	spindlin 1
1656	8173198	SPIN2A	-2.2068632	-3.6179	-0.87674	0.0398207	spindlin family member 2A
1657	8173181	SPIN3	-2.3671768	-3.3878	-0.55463	0.0286898	spindlin family member 3
1658	8154779	SPINK4	-2.3557903	-3.4044	-0.5521	0.0293736	serine peptidase inhibitor, Kazal type 4
1659	7982829	SPINT1	-2.1774219	-3.6594	-0.66116	0.0422528	serine peptidase inhibitor, Kunitz type 1
1660	7994603	SPN	2.4664866	-3.2417	0.969022	0.0233242	sialophorin
1661	8045514	SPOPL	-2.7118933	-2.8712	-0.84994	0.0138264	speckle type BTB/POZ protein like
1662	7988753	SPPL2A	-2.5171072	-3.1663	-0.44878	0.0209656	signal peptide peptidase like 2A
1663	8042696	SPR	-3.6683477	-1.362	-1.09171	0.0016326	sepiapterin reductase (7,8-dihydrobiopterin:NADP+ oxidoreductase)
1664	7920196	SPRR2D	-2.5757273	-3.0783	-4.46464	0.018515	small proline rich protein 2D
1665	7963577	SPRYD3	-2.444587	-3.2741	-0.44565	0.0244198	SPRY domain containing 3
1666	8083071	SPSB4	2.3209451	-3.4549	0.547218	0.0315612	splA/ryanodine receptor domain and SOCS box containing 4
1667	8091799	SPTSSB	-2.8421514	-2.6701	-4.96094	0.0104169	serine palmitoyltransferase small subunit B
1668	7983512	SQRDL	-2.8905925	-2.5947	-0.66506	0.0093677	sulfide quinone reductase-like (yeast)
1669	8110569	SQSTM1	-2.4634634	-3.2462	-0.5086	0.0234726	sequestosome 1
1670	8014679	SRCIN1	-2.2350995	-3.578	-1.4695	0.0376093	SRC kinase signaling inhibitor 1
1671	8112337	SREK1IP1	-2.726787	-2.8483	-0.61746	0.0133884	SREK1 interacting protein 1
1672	7956759	SRGAP1	-2.3129045	-3.4665	-0.8208	0.032087	SLIT-ROBO Rho GTPase activating protein 1
1673	8107350	SRP19	-2.2501023	-3.5566	-0.99069	0.0364808	signal recognition particle 19
1674	8128079	SRSF12	3.3143095	-1.9258	0.624327	0.0036444	serine and arginine rich splicing factor 12
1675	7941843	SSH3	-2.7805229	-2.7656	-0.90773	0.0119154	slingshot protein phosphatase 3
1676	8070863	SSR4P1	2.4045935	-3.3331	0.386589	0.0265457	signal sequence receptor subunit 4 pseudogene 1
1677	7985253	ST20-AS1	2.2867781	-3.5042	0.519799	0.0338519	ST20 antisense RNA 1

1678	7990810	ST20-MTH	-2.5687548	-3.0888	-0.41982	0.0187916	ST20-MTHFS readthrough///5,10-methenyltetrahydrofolate synthetas
1679	8002370	ST3GAL2	3.7493778	-1.2332	0.699166	0.0013571	ST3 beta-galactoside alpha-2,3-sialyltransferase 2
1680	7961757	ST8SIA1	3.4760882	-1.6681	1.653232	0.0025277	ST8 alpha-N-acetyl-neuraminide alpha-2,8-sialyltransferase 1
1681	7932407	ST8SIA6	-2.4240379	-3.3045	-2.11961	0.0254914	ST8 alpha-N-acetyl-neuraminide alpha-2,8-sialyltransferase 6
1682	8055890	STAM2	-2.1335209	-3.7206	-0.4721	0.0461319	signal transducing adaptor molecule 2
1683	8095343	STAP1	4.0425452	-0.7704	2.284793	0.0006948	signal transducing adaptor family member 1
1684	7950235	STARD10	-2.6266474	-3.0013	-1.92295	0.0166082	StAR related lipid transfer domain containing 10
1685	8132376	STARD3NL	-2.2175471	-3.6028	-0.55355	0.0389703	STARD3 N-terminal like
1686	8023394	STARD6	2.2464532	-3.5618	0.758214	0.0367524	StAR related lipid transfer domain containing 6
1687	8007212	STAT5A	2.1273498	-3.7292	0.687542	0.0467024	signal transducer and activator of transcription 5A
1688	8115851	STC2	-3.4239547	-1.7512	-2.96024	0.0028447	stanniocalcin 2
1689	8138527	STEAP1B	2.194211	-3.6358	0.830422	0.0408498	STEAP family member 1B
1690	8134036	STEAP2	-2.2113519	-3.6116	-0.84742	0.0394614	STEAP2 metalloreductase
1691	8024204	STK11	-2.2773577	-3.5177	-0.46737	0.0345099	serine/threonine kinase 11
1692	8048505	STK16	-2.2107032	-3.6125	-0.68127	0.0395132	serine/threonine kinase 16
1693	8093858	STK32B	-2.3740752	-3.3777	-1.57489	0.0282828	serine/threonine kinase 32B
1694	8048381	STK36	-2.2959436	-3.491	-0.64952	0.0332228	serine/threonine kinase 36
1695	8149898	STMN4	2.5167777	-3.1668	0.649606	0.0209802	stathmin 4
1696	8098549	STOX2	2.9439574	-2.5113	1.643196	0.0083297	storkhead box 2
1697	7978428	STRN3	-2.0978846	-3.7698	-0.37906	0.0495159	striatin 3
1698	8000590	SULT1A1	-2.5017259	-3.1893	-0.96524	0.0216574	sulfotransferase family 1A member 1
1699	8000582	SULT1A2	-2.2875452	-3.5031	-0.89583	0.0337988	sulfotransferase family 1A member 2
1700	8174636	SUMO4///!	-2.5479149	-3.1201	-0.53517	0.0196419	small ubiquitin-like modifier 4///small ubiquitin-like modifier 2
1701	8018315	SUMO4///!	-2.4124337	-3.3215	-0.50974	0.0261158	small ubiquitin-like modifier 4///small ubiquitin-like modifier 2
1702	8178322	SUMO4///!	-2.3994292	-3.3406	-0.50676	0.0268325	small ubiquitin-like modifier 4///small ubiquitin-like modifier 2
1703	8159008	SURF2	-2.1541838	-3.6919	-0.78224	0.0442674	surfeit 2
1704	7932796	SVIL	2.6976505	-2.893	0.984304	0.0142581	supervillin
1705	8077879	SYN2	2.1125157	-3.7497	1.079086	0.0481002	synapsin II
1706	8075616	SYN3	3.8261159	-1.1116	1.073096	0.0011391	synapsin III
1707	7914557	SYNC	-2.9872328	-2.4435	-1.01586	0.0075704	syncoilin, intermediate filament protein
1708	7947590	SYT13	-2.9632044	-2.4812	-4.32666	0.0079833	synaptotagmin 13
1709	7993624	SYT17	-2.3891494	-3.3557	-1.70406	0.027412	synaptotagmin 17
1710	7950810	SYTL2	-2.4449128	-3.2737	-1.86048	0.0244031	synaptotagmin like 2
1711	8173955	SYTL4	-2.3795372	-3.3698	-1.41782	0.0279643	synaptotagmin like 4
1712	7898328	SZRD1	-2.0962338	-3.7721	-0.4308	0.049678	SUZ RNA binding domain containing 1
1713	8122131	TAAR6	2.2232478	-3.5948	0.485229	0.0385234	trace amine associated receptor 6
1714	7916584	TACSTD2	-2.3205088	-3.4556	-1.0453	0.0315895	tumor-associated calcium signal transducer 2
1715	8020702	TAF4B	3.1908479	-2.122	1.168431	0.0048101	TATA-box binding protein associated factor 4b
1716	8173732	TAF9B	-2.6825464	-2.9161	-0.72621	0.0147299	TATA-box binding protein associated factor 9b
1717	8176263	TAF9B	-2.6825464	-2.9161	-0.72621	0.0147299	TATA-box binding protein associated factor 9b
1718	8130539	TAGAP	2.1425686	-3.708	1.048434	0.0453069	T-cell activation RhoGTPase activating protein
1719	8081620	TAGLN3	2.2140039	-3.6079	0.679006	0.0392505	transgelin 3
1720	7994692	TAOK2	-2.6396202	-2.9816	-0.59052	0.0161531	TAO kinase 2
1721	8099506	TAPT1	-3.0184523	-2.3944	-0.88065	0.0070646	transmembrane anterior posterior transformation 1
1722	7897663	TARDBP	-2.4962909	-3.1974	-0.37649	0.021907	TAR DNA binding protein
1723	8136645	TAS2R4	-2.1818953	-3.6531	-1.03054	0.0418747	taste 2 receptor member 4
1724	8136846	TAS2R40	-2.1661544	-3.6751	-0.64661	0.043219	taste 2 receptor member 40
1725	8077804	TATDN2	2.1545012	-3.6914	0.563495	0.0442393	TatD DNase domain containing 2
1726	8000856	TBC1D10B	-2.3796987	-3.3695	-0.65638	0.0279549	TBC1 domain family member 10B
1727	7941879	TBC1D10C	3.4430553	-1.7208	0.918115	0.0027242	TBC1 domain family member 10C
1728	8030539	TBC1D17	-2.2427232	-3.5671	-0.44281	0.0370319	TBC1 domain family member 17
1729	7984759	TBC1D21	3.0099181	-2.4078	0.611181	0.0071995	TBC1 domain family member 21
1730	7956826	TBC1D30	-2.5213654	-3.1599	-1.22949	0.0207778	TBC1 domain family member 30
1731	7972021	TBC1D4	2.1207308	-3.7383	0.68018	0.0473215	TBC1 domain family member 4
1732	8054308	TBC1D8	-3.7025726	-1.3076	-0.98952	0.00151	TBC1 domain family member 8
1733	8102912	TBC1D9	-2.3452591	-3.4197	-1.46047	0.0300194	TBC1 domain family member 9
1734	7966690	TBX3	-2.8869992	-2.6003	-1.50356	0.0094419	T-box 3
1735	8143597	TCAF1	-2.587616	-3.0604	-0.60301	0.0180521	TRPM8 channel associated factor 1
1736	8136940	TCAF2P1//	2.1622469	-3.6806	0.837433	0.0435587	TRPM8 channel associated factor 2 pseudogene 1///TRPM8 channel
1737	8136954	TCAF2P1//	2.1539229	-3.6922	0.767991	0.0442905	TRPM8 channel associated factor 2 pseudogene 1///TRPM8 channel
1738	7913593	TCEA3	-3.2504003	-2.0275	-0.99732	0.0042083	transcription elongation factor A3
1739	8168892	TCEAL2	2.1656863	-3.6758	0.543841	0.0432595	transcription elongation factor A like 2
1740	8040827	TCF23	2.1980747	-3.6303	0.356964	0.040533	transcription factor 23
1741	8108050	TCF7	3.1220379	-2.231	1.083427	0.0056104	transcription factor 7 (T-cell specific, HMG-box)
1742	7948444	TCN1	2.3353508	-3.4341	1.773821	0.0306391	transcobalamin 1
1743	8072360	TCN2	2.3698816	-3.3839	0.750662	0.0285296	transcobalamin 2
1744	7958692	TCTN1	-2.6130445	-3.0219	-0.70617	0.0170986	tectonic family member 1
1745	7959638	TCTN2	-2.8873342	-2.5998	-0.84826	0.009435	tectonic family member 2
1746	8156688	TDRD7	-2.6697314	-2.9357	-0.72044	0.0151418	tudor domain containing 7
1747	7983828	TEX9	-2.4852879	-3.2138	-1.35189	0.0224205	testis expressed 9
1748	7925611	TFB2M	-2.8521445	-2.6546	-0.59507	0.0101916	transcription factor B2, mitochondrial
1749	7963244	TFCP2	-2.738191	-2.8308	-0.61349	0.0130619	transcription factor CP2
1750	8126312	TFEB	2.3394927	-3.4281	0.481932	0.0303786	transcription factor EB

1751	8070579	TFF1	-2.1497383	-3.6981	-2.85083	0.0446627	trefoil factor 1
1752	8070567	TFF3	-2.613275	-3.0215	-2.44223	0.0170901	trefoil factor 3
1753	8019939	TGIF1	-2.1501772	-3.6974	-0.42809	0.0446235	TGFB induced factor homeobox 1
1754	7990086	THAP10	-2.7703383	-2.7813	-0.71622	0.0121821	THAP domain containing 10
1755	7957161	THAP2	-2.5801966	-3.0716	-1.10207	0.0183397	THAP domain containing 2
1756	8144151	THAP5P1	-2.2349983	-3.5781	-0.6868	0.0376171	THAP domain containing 5 pseudogene 1
1757	8043393	THNSL2	-2.3496054	-3.4134	-1.59201	0.0297513	threonine synthase like 2
1758	8088526	THOC7	-3.0996698	-2.2663	-0.62939	0.0058975	THO complex 7
1759	7984588	THSD4	-3.1201701	-2.2339	-2.20188	0.0056339	thrombospondin type 1 domain containing 4
1760	8114239	TIFAB///DC	2.4263988	-3.301	0.841778	0.0253661	TIFA inhibitor///dendritic cell associated nuclear protein
1761	8083569	TIPARP	-3.3182222	-1.9196	-1.01296	0.0036124	TCDD inducible poly(ADP-ribose) polymerase
1762	7989915	TIPIN	-2.5432445	-3.1272	-0.43657	0.0198374	TIMELESS interacting protein
1763	7986977	TJP1	-2.5635905	-3.0966	-0.61929	0.018999	tight junction protein 1
1764	8024687	TJP3	-2.702556	-2.8855	-1.01422	0.014108	tight junction protein 3
1765	7940451	TKFC	-3.2043709	-2.1005	-1.00763	0.0046665	triokinase and FMN cyclase
1766	8099826	TLR10	2.5518407	-3.1143	1.360352	0.019479	toll like receptor 10
1767	8109969	TLX3	-2.7172486	-2.863	-0.52081	0.0136674	T-cell leukemia homeobox 3
1768	7916643	TM2D1	-2.180187	-3.6555	-0.62434	0.0420187	TM2 domain containing 1
1769	7961983	TM7SF3	2.3918368	-3.3518	0.622747	0.0272594	transmembrane 7 superfamily member 3
1770	8039144	TMC4	-3.1801966	-2.1389	-1.32972	0.0049263	transmembrane channel like 4
1771	8010212	TMC8	2.4366512	-3.2859	1.124626	0.0248285	transmembrane channel like 8
1772	7970301	TMCO3	-3.2552393	-2.0198	-0.79303	0.0041628	transmembrane and coiled-coil domains 3
1773	8004404	TMEM102	2.1766934	-3.6604	0.300055	0.0423147	transmembrane protein 102
1774	8131539	TMEM106E	-2.1392375	-3.7127	-0.58186	0.0456091	transmembrane protein 106B
1775	8088047	TMEM110-	2.3650959	-3.3908	0.378846	0.0288137	TMEM110-MUSTN1 readthrough///musculoskeletal, embryonic nucle
1776	7900654	TMEM125	-2.1407183	-3.7106	-0.80745	0.0454745	transmembrane protein 125
1777	8099099	TMEM128	-2.1672215	-3.6736	-0.46746	0.0431266	transmembrane protein 128
1778	8159379	TMEM141	-2.8545021	-2.6509	-0.94265	0.0101391	transmembrane protein 141
1779	8098041	TMEM144	-2.4518515	-3.2634	-0.76877	0.0240511	transmembrane protein 144
1780	8120239	TMEM14A	-3.2673107	-2.0006	-0.87241	0.0040513	transmembrane protein 14A
1781	8116867	TMEM14B	-2.3860981	-3.3602	-0.53985	0.0275862	transmembrane protein 14B
1782	8116859	TMEM14C	-2.8011417	-2.7337	-0.6221	0.0113924	transmembrane protein 14C
1783	8099850	TMEM156	3.1926759	-2.1191	1.75757	0.0047905	transmembrane protein 156
1784	8113023	TMEM161E	-2.3416517	-3.4249	-0.47664	0.0302437	transmembrane protein 161B
1785	8093413	TMEM175	-2.3620048	-3.3953	-0.55163	0.0289985	transmembrane protein 175
1786	8041570	TMEM178/	-2.7413804	-2.8259	-0.45863	0.012972	transmembrane protein 178A
1787	8137783	TMEM184/	-2.9354993	-2.5246	-1.60019	0.0084865	transmembrane protein 184A
1788	8097704	TMEM184(	-2.6075462	-3.0302	-0.62463	0.0173006	transmembrane protein 184C
1789	8048628	TMEM198	2.6245309	-3.0045	0.449216	0.0166837	transmembrane protein 198
1790	8113483	TMEM232	-2.1149506	-3.7463	-1.18667	0.0478682	transmembrane protein 232
1791	7914500	TMEM234	-2.4390042	-3.2824	-0.62073	0.0247067	transmembrane protein 234
1792	7944275	TMEM25	-3.3463361	-1.8749	-0.96342	0.0033904	transmembrane protein 25
1793	8026579	TMEM38A	2.2413005	-3.5692	0.793003	0.037139	transmembrane protein 38A
1794	7946504	TMEM41B	-2.4360902	-3.2867	-0.56895	0.0248577	transmembrane protein 41B
1795	8077993	TMEM43	2.4153005	-3.3173	0.678626	0.0259602	transmembrane protein 43
1796	7945169	TMEM45B	-2.6072485	-3.0307	-1.86743	0.0173116	transmembrane protein 45B
1797	7956749	TMEM5///T	-2.4368069	-3.2856	-0.58429	0.0248205	transmembrane protein 5///transmembrane protein 5
1798	7899005	TMEM50A	-2.6135945	-3.0211	-0.44257	0.0170785	transmembrane protein 50A
1799	7916372	TMEM59	-2.2780609	-3.5167	-0.48803	0.0344604	transmembrane protein 59
1800	8147313	TMEM67	-3.3904462	-1.8046	-0.90293	0.0030688	transmembrane protein 67
1801	8152976	TMEM71	2.4655309	-3.2431	1.378508	0.023371	transmembrane protein 71
1802	7987869	TMEM87A	-2.9103102	-2.564	-0.49026	0.0089705	transmembrane protein 87A
1803	8155148	TMEM8B	-2.4366488	-3.2859	-0.68661	0.0248287	transmembrane protein 8B
1804	7957478	TMTC3	-2.2213564	-3.5975	-0.48678	0.0386712	transmembrane and tetratricopeptide repeat containing 3
1805	8122265	TNFAIP3	3.8671976	-1.0466	1.151069	0.0010371	TNF alpha induced protein 3
1806	8021528	TNFRSF11/	2.4833806	-3.2166	1.009117	0.0225107	TNF receptor superfamily member 11a
1807	7993267	TNFRSF17	2.6416552	-2.9785	1.607141	0.0160828	TNF receptor superfamily member 17
1808	7897877	TNFRSF1B	2.0989188	-3.7684	0.77643	0.0494146	TNF receptor superfamily member 1B
1809	7911413	TNFRSF4	2.3151073	-3.4634	0.470931	0.0319422	TNF receptor superfamily member 4
1810	8004372	TNK1///TN	-3.1186874	-2.2363	-0.80695	0.0056525	tyrosine kinase non receptor 1///tyrosine kinase non receptor 1
1811	7907549	TNN	2.14268	-3.7079	1.379924	0.0452968	tenascin N
1812	8087925	TNNC1	-2.284565	-3.5073	-0.7362	0.0340054	troponin C1, slow skeletal and cardiac type
1813	7923360	TNNI1	2.3235291	-3.4512	0.557909	0.0313939	troponin I1, slow skeletal type
1814	8016739	TOB1	-2.714711	-2.8669	-0.71233	0.0137425	transducer of ERBB2, 1
1815	8008547	TOM1L1	-2.8907258	-2.5945	-0.99137	0.009365	target of myb1 like 1 membrane trafficking protein
1816	8150962	TOX	2.2153581	-3.6059	1.06552	0.0391432	thymocyte selection associated high mobility group box
1817	8001394	TOX3	-3.4425741	-1.7216	-2.20413	0.0027272	TOX high mobility group box family member 3
1818	8140739	TP53TG1	-2.3172279	-3.4603	-0.70398	0.0318033	TP53 target 1 (non-protein coding)
1819	7911854	TP73-AS1	-2.2138149	-3.6081	-0.48278	0.0392655	TP73 antisense RNA 1
1820	8120880	TPBG	-3.9554725	-0.9073	-1.49366	0.0008477	trophoblast glycoprotein
1821	8053379	TRABD2A	2.2297825	-3.5855	0.621215	0.0380169	TraB domain containing 2A
1822	8163825	TRAF1	2.1921357	-3.6387	0.685982	0.0410209	TNF receptor associated factor 1
1823	8049635	TRAF3IP1	-2.5751512	-3.0792	-0.53124	0.0185377	TRAF3 interacting protein 1

1824	7909455	TRAF3IP3	2.3823369	-3.3657	1.116619	0.0278024 TRAF3 interacting protein 3
1825	7973221	TRAJ17///T	2.2456085	-3.563	1.370494	0.0368155 T cell receptor alpha joining 17///T cell receptor alpha variable 12-2
1826	8105661	TRAPPC13	-2.8614597	-2.6401	-3.57966	0.0099858 trafficking protein particle complex 13
1827	8060344	TRIB3	-2.1426963	-3.7079	-0.95831	0.0452954 tribbles pseudokinase 3
1828	7946180	TRIM3	-2.1084009	-3.7553	-0.82107	0.0484946 tripartite motif containing 3
1829	7913907	TRIM63	2.7313935	-2.8413	0.865786	0.0132556 tripartite motif containing 63
1830	7938012	TRIM6-TRI	-2.2026804	-3.6238	-0.8212	0.0401583 TRIM6-TRIM34 readthrough///tripartite motif containing 6///tripartit
1831	8098697	TRIML1	2.7175328	-2.8625	1.01446	0.013659 tripartite motif family like 1
1832	7984190	TRIP4	-2.7699568	-2.7819	-0.48909	0.0121922 thyroid hormone receptor interactor 4
1833	7949075	TRMT112	-2.3220132	-3.4534	-0.45114	0.031492 tRNA methyltransferase 11-2 homolog (S. cerevisiae)
1834	7908421	TROVE2	-2.7072565	-2.8783	-0.66053	0.0139656 TROVE domain family member 2
1835	8102678	TRPC3	2.5118213	-3.1742	0.653734	0.021201 transient receptor potential cation channel subfamily C member 3
1836	7949046	TRPT1	-2.3553496	-3.405	-0.53908	0.0294004 tRNA phosphotransferase 1
1837	8011354	TRPV3	2.6208374	-3.0101	1.17313	0.016816 transient receptor potential cation channel subfamily V member 3
1838	8134470	TRRAP	2.1283439	-3.7278	0.397237	0.0466101 transformation/transcription domain associated protein
1839	8174361	TSC22D3	-2.2035608	-3.6226	-0.56925	0.040087 TSC22 domain family member 3
1840	8141526	TSC22D4	2.7819888	-2.7633	0.782257	0.0118774 TSC22 domain family member 4
1841	7910550	TSNAX-DIS	-3.4224345	-1.7536	-0.81321	0.0028545 TSNAX-DISC1 readthrough (NMD candidate)///translin associated fa
1842	7901175	TSPAN1	-4.7667366	0.3369	-4.72819	0.000134 tetraspanin 1
1843	8131600	TSPAN13	-2.3728517	-3.3795	-1.15204	0.0283546 tetraspanin 13
1844	7928046	TSPAN15	-2.4732434	-3.2317	-1.10391	0.0229956 tetraspanin 15
1845	7956613	TSPAN31/,	-2.3502044	-3.4125	-0.77682	0.0297145 tetraspanin 31///tetraspanin 31
1846	8136067	TSPAN33	2.4528929	-3.2618	0.956389	0.0239987 tetraspanin 33
1847	8035684	TSSK6	-2.222052	-3.5965	-0.97155	0.0386168 testis specific serine kinase 6
1848	8162676	TSTD2	-2.2113988	-3.6115	-0.46498	0.0394577 thiosulfate sulfurtransferase like domain containing 2
1849	8136495	TTC26	-2.1114261	-3.7512	-0.70017	0.0482044 tetratricopeptide repeat domain 26
1850	8056999	TTC30A	-3.5389704	-1.5679	-1.15363	0.0021914 tetratricopeptide repeat domain 30A
1851	7916024	TTC39A	-2.4648347	-3.2442	-1.75028	0.0234052 tetratricopeptide repeat domain 39A
1852	7965935	TTC41P	-2.5342534	-3.1406	-1.58102	0.0202189 tetratricopeptide repeat domain 41, pseudogene
1853	7974008	TTC6	-3.6136402	-1.449	-2.6865	0.0018492 tetratricopeptide repeat domain 6
1854	7974029	TTC6	-2.6941665	-2.8983	-2.69018	0.0143656 tetratricopeptide repeat domain 6
1855	7980720	TTC7B	3.2766419	-1.9857	0.694659	0.0039671 tetratricopeptide repeat domain 7B
1856	7976128	TTC8	-3.1558049	-2.1775	-0.98892	0.0052027 tetratricopeptide repeat domain 8
1857	8057056	TTN	2.4768494	-3.2263	1.851341	0.022822 titin
1858	8151042	TTPA	-3.44064	-1.7246	-1.52224	0.0027392 alpha tocopherol transfer protein
1859	8031157	TTYH1	2.6901609	-2.9045	1.681877	0.0144902 tweety family member 1
1860	8116649	TUBB2A	2.3113814	-3.4687	0.481318	0.0321875 tubulin beta 2A class IIa
1861	8116653	TUBB2A	2.3113814	-3.4687	0.481318	0.0321875 tubulin beta 2A class IIa
1862	7998063	TUBB3///M	2.2333736	-3.5804	0.610653	0.0377412 tubulin beta 3 class III///melanocortin 1 receptor
1863	8159642	TUBB4B	-2.4349614	-3.2884	-0.54686	0.0249164 tubulin beta 4B class IVb
1864	7931674	TUBB8	2.2923001	-3.4962	0.379059	0.0334715 tubulin beta 8 class VIII
1865	7981798	TUBGCP5	-2.3433758	-3.4224	-0.54627	0.0301363 tubulin gamma complex associated protein 5
1866	8013906	TWF1	-3.422557	-1.7535	-0.74664	0.0028537 twinfilin actin binding protein 1
1867	7962441	TWF1	-3.2918125	-1.9616	-0.71179	0.0038339 twinfilin actin binding protein 1
1868	8100210	TXK	2.2104395	-3.6129	1.029788	0.0395342 TXK tyrosine kinase
1869	8058108	TYW5	-2.9249292	-2.5411	-0.45079	0.0086864 tRNA-yW synthesizing protein 5
1870	8068788	UBASH3A	2.2096403	-3.614	1.63635	0.0395981 ubiquitin associated and SH3 domain containing A
1871	8049582	UBE2F-SCL	-2.5768639	-3.0766	-0.89621	0.0184702 UBE2F-SCLY readthrough (NMD candidate)///selenocysteine lyase
1872	7986769	UBE3A	-2.6420622	-2.9778	-0.51833	0.0160687 ubiquitin protein ligase E3A
1873	7958532	UBE3B	-2.520805	-3.1608	-0.54186	0.0208024 ubiquitin protein ligase E3B
1874	7913918	UBXN11	-2.4861444	-3.2125	-0.58344	0.0223801 UBX domain protein 11
1875	8145691	UBXN8	-2.8616181	-2.6399	-0.89507	0.0099823 UBX domain protein 8
1876	7947027	UEVLD	-2.6505539	-2.9649	-0.69418	0.0157787 UEV and lactate/malate dehyrogenase domains
1877	7968670	UFM1	-2.4098505	-3.3253	-0.35133	0.0262567 ubiquitin fold modifier 1
1878	8141643	UFSP1	2.1032871	-3.7624	0.591884	0.0489888 UFM1 specific peptidase 1 (inactive)
1879	8099897	UGDH	-3.3537147	-1.8631	-1.41775	0.0033344 UDP-glucose 6-dehydrogenase
1880	8097017	UGT8	2.2615204	-3.5403	1.723639	0.0356428 UDP glycosyltransferase 8
1881	7965723	UHRF1BP1	-3.6116933	-1.4521	-0.77092	0.0018574 UHRF1 binding protein 1 like
1882	8035880	UQCRFS1	2.2464515	-3.5618	0.526614	0.0367525 ubiquinol-cytochrome c reductase, Rieske iron-sulfur polypeptide 1
1883	8069943	URB1	2.3358959	-3.4333	0.60941	0.0306047 URB1 ribosome biogenesis 1 homolog (S. cerevisiae)
1884	8018258	USH1G	2.5917866	-3.0541	0.607704	0.0178923 USH1 protein network component sans
1885	7958439	USP30	-2.5071338	-3.1812	-0.5729	0.0214118 ubiquitin specific peptidase 30
1886	8059801	USP40	-3.6634772	-1.3697	-0.82774	0.0016508 ubiquitin specific peptidase 40
1887	8106193	UTP15	-2.2145044	-3.6072	-0.40368	0.0392108 UTP15, small subunit processome component
1888	7897370	VAMP3	-2.9197443	-2.5492	-0.52393	0.0087862 vesicle associated membrane protein 3
1889	8020129	VAPA	-2.1116217	-3.7509	-0.32819	0.0481856 VAMP associated protein A
1890	7918157	VAV3	-2.172881	-3.6657	-1.30257	0.0426398 vav guanine nucleotide exchange factor 3
1891	8042532	VAX2	2.6828433	-2.9157	0.482966	0.0147205 ventral anterior homeobox 2
1892	7978923	VCPKMT	-2.740434	-2.8274	-0.59908	0.0129986 valosin containing protein lysine methyltransferase
1893	8016891	VEZF1	-2.8486099	-2.6601	-0.59878	0.0102707 vascular endothelial zinc finger 1
1894	7957613	VEZT	-2.1581389	-3.6863	-0.496	0.0439185 vezatin, adherens junctions transmembrane protein
1895	8170179	VGLL1	2.7401232	-2.8278	1.881651	0.0130074 vestigial like family member 1
1896	7948504	VPS37C	-2.2112578	-3.6117	-0.71056	0.0394689 VPS37C, ESCRT-I subunit

1897	7987840	VPS39	-2.2359018	-3.5768	-0.35805	0.0375482	VPS39, HOPS complex subunit
1898	7966839	VSIG10	-2.1804956	-3.6551	-0.62824	0.0419927	V-set and immunoglobulin domain containing 10
1899	8142671	WASL	-2.2652623	-3.535	-0.53525	0.0353721	Wiskott-Aldrich syndrome like
1900	7927405	WDFY4	2.9983572	-2.426	1.416156	0.0073863	WDFY family member 4
1901	7927425	WDFY4	2.8581508	-2.6453	1.336647	0.0100584	WDFY family member 4
1902	7976766	WDR25	-2.0992752	-3.7679	-0.53434	0.0493797	WD repeat domain 25
1903	8163491	WDR31	-2.7861191	-2.7569	-0.57927	0.0117712	WD repeat domain 31
1904	8157818	WDR38	2.2037159	-3.6224	0.373887	0.0400745	WD repeat domain 38
1905	8091922	WDR49	-2.1078077	-3.7562	-0.67427	0.0485517	WD repeat domain 49
1906	7959330	WDR66	-3.3349817	-1.8929	-1.0831	0.0034784	WD repeat domain 66
1907	7991126	WDR73	-2.8006318	-2.7345	-0.53137	0.0114051	WD repeat domain 73
1908	8066574	WFDC10B	2.4088592	-3.3268	0.729482	0.026311	WAP four-disulfide core domain 10B
1909	8093906	WFS1	-2.4259377	-3.3017	-1.04302	0.0253905	wolframin ER transmembrane glycoprotein
1910	7964722	WIF1	2.4472554	-3.2702	3.384427	0.0242837	WNT inhibitory factor 1
1911	7924832	WNT9A	-2.9236218	-2.5432	-0.85532	0.0087115	Wnt family member 9A
1912	8147156	WWP1	-2.7127081	-2.8699	-1.05785	0.0138021	WW domain containing E3 ubiquitin protein ligase 1
1913	7936284	XPNPEP1	2.0974211	-3.7705	0.45391	0.0495614	X-prolyl aminopeptidase 1
1914	7999754	XYLT1	2.3997498	-3.3402	0.951094	0.0268146	xylosyltransferase 1
1915	8132399	YAE1D1	-2.2383873	-3.5733	-0.41917	0.0373593	Yae1 domain containing 1
1916	7916343	YIPF1	-2.0991038	-3.7681	-0.69278	0.0493965	Yip1 domain family member 1
1917	8114829	YIPF5	-2.1019224	-3.7643	-0.40434	0.0491215	Yip1 domain family member 5
1918	8043725	ZAP70	2.3515015	-3.4106	1.095998	0.029635	zeta chain of T cell receptor associated protein kinase 70
1919	8091887	ZBBX	-2.2774106	-3.5176	-4.19683	0.0345062	zinc finger B-box domain containing
1920	8115600	ZBED8	-4.2324904	-0.4742	-1.43123	0.0004503	zinc finger BED-type containing 8
1921	8081358	ZBTB11-AS	-2.5777082	-3.0753	-0.6654	0.0184371	ZBTB11 antisense RNA 1
1922	7923119	ZBTB41	-2.9594802	-2.487	-0.74954	0.0080493	zinc finger and BTB domain containing 41
1923	7977294	ZBTB42	-2.6530482	-2.9611	-0.62193	0.0156944	zinc finger and BTB domain containing 42
1924	8039791	ZBTB45	-3.2832586	-1.9752	-0.62388	0.0039085	zinc finger and BTB domain containing 45
1925	8067743	ZBTB46	2.4044079	-3.3333	0.50617	0.026556	zinc finger and BTB domain containing 46
1926	8023212	ZBTB7C	-2.1985296	-3.6297	-1.10947	0.0404958	zinc finger and BTB domain containing 7C
1927	7914550	ZBTB8OS	-2.5355067	-3.1388	-0.52598	0.0201653	zinc finger and BTB domain containing 8 opposite strand
1928	8173261	ZC4H2	2.1448085	-3.7049	1.212061	0.0451047	zinc finger C4H2-type containing
1929	7916185	ZCCHC11	2.5435074	-3.1268	0.911565	0.0198263	zinc finger CCHC-type containing 11
1930	8094393	ZCCHC4	-2.6568013	-2.9554	-0.78008	0.0155685	zinc finger CCHC-type containing 4
1931	8002029	ZDHHC1	-2.1168889	-3.7436	-0.51518	0.0476842	zinc finger DHHC-type containing 1
1932	7899253	ZDHHC18/	2.4444049	-3.2744	0.520225	0.0244291	zinc finger DHHC-type containing 18///zinc finger DHHC-type conta
1933	8131414	ZDHHC4	-2.4819087	-3.2188	-0.66134	0.0225805	zinc finger DHHC-type containing 4
1934	7985259	ZFAND6	-2.2713874	-3.5262	-0.54939	0.034933	zinc finger AN1-type containing 6
1935	8153043	ZFAT	2.0951855	-3.7735	0.813074	0.0497811	zinc finger and AT-hook domain containing
1936	8176384	ZFY	2.1338127	-3.7202	0.370903	0.0461051	zinc finger protein, Y-linked
1937	7982812	ZFYVE19	-2.1335207	-3.7206	-0.41801	0.0461319	zinc finger FYVE-type containing 19
1938	8000399	ZKSCAN2	-2.3477914	-3.416	-0.92535	0.029863	zinc finger with KRAB and SCAN domains 2
1939	7915425	ZMYND12	-2.1116244	-3.7509	-0.91486	0.0481854	zinc finger MYND-type containing 12
1940	8033789	ZNF121	2.3763375	-3.3744	0.569527	0.0281505	zinc finger protein 121
1941	8031744	ZNF17	-2.1005057	-3.7662	-0.45058	0.0492595	zinc finger protein 17
1942	7946288	ZNF214	-2.8251392	-2.6965	-0.87255	0.0108114	zinc finger protein 214
1943	8029347	ZNF230	-2.9931449	-2.4342	-1.38446	0.007472	zinc finger protein 230
1944	8031690	ZNF264	-2.1183623	-3.7416	-0.38709	0.0475448	zinc finger protein 264
1945	8005110	ZNF286B//	2.8439491	-2.6673	0.83798	0.010376	zinc finger protein 286B///zinc finger protein 286A
1946	8013305	ZNF286B//	2.2692095	-3.5293	0.749211	0.0350886	zinc finger protein 286B///zinc finger protein 286A
1947	7963774	ZNF385A	-3.0821939	-2.294	-1.17004	0.0061316	zinc finger protein 385A
1948	8031962	ZNF446	-2.4651796	-3.2436	-0.5177	0.0233883	zinc finger protein 446
1949	8157105	ZNF462	2.1109528	-3.7518	1.140323	0.0482497	zinc finger protein 462
1950	8143749	ZNF467///.	-2.6103608	-3.026	-0.86611	0.0171969	zinc finger protein 467///zinc finger protein 467
1951	7927383	ZNF488	2.3261981	-3.4473	0.51653	0.031222	zinc finger protein 488
1952	7928529	ZNF503	2.6462098	-2.9715	0.588144	0.0159264	zinc finger protein 503
1953	7934553	ZNF503	-2.6185196	-3.0136	-1.01286	0.0168996	zinc finger protein 503
1954	8022612	ZNF521	3.4845575	-1.6547	1.835924	0.0024796	zinc finger protein 521
1955	8031720	ZNF543	-2.4113017	-3.3232	-0.58741	0.0261775	zinc finger protein 543
1956	8031884	ZNF544	-2.2058157	-3.6194	-0.39996	0.039905	zinc finger protein 544
1957	8039687	ZNF552	-3.4362266	-1.7317	-1.86854	0.0027667	zinc finger protein 552
1958	7984203	ZNF609	-2.4230911	-3.3059	-0.52747	0.0255418	zinc finger protein 609
1959	7922686	ZNF648	-2.2408753	-3.5698	-2.72064	0.0371711	zinc finger protein 648
1960	8081069	ZNF654	-2.9101364	-2.5642	-0.74142	0.0089739	zinc finger protein 654
1961	8133042	ZNF679	2.3297702	-3.4422	0.434401	0.0309933	zinc finger protein 679
1962	8148615	ZNF696	2.2202996	-3.599	0.387834	0.038754	zinc finger protein 696
1963	7919051	ZNF697	2.7413581	-2.8259	0.653511	0.0129726	zinc finger protein 697
1964	8145829	ZNF703	-3.0434469	-2.3551	-1.61734	0.0066834	zinc finger protein 703
1965	8088911	ZNF717	-2.1101502	-3.7529	-0.5883	0.0483266	zinc finger protein 717
1966	7987361	ZNF770	-2.5213572	-3.16	-0.46221	0.0207781	zinc finger protein 770
1967	8031815	ZNF776	-2.6043723	-3.035	-0.47838	0.0174183	zinc finger protein 776
1968	8039692	ZNF814	-2.458	-3.2543	-0.88932	0.0237432	zinc finger protein 814
1969	8063723	ZNF831	2.5649596	-3.0945	0.979331	0.0189438	zinc finger protein 831

1970	8039605	ZNF835	2.2604183	-3.5419	0.524625	0.0357229	zinc finger protein 835
1971	8024934	ZNRF4	2.1107571	-3.7521	0.794216	0.0482684	zinc and ring finger 4
1972	8022872	ZSCAN30	-2.240556	-3.5702	-0.56016	0.0371952	zinc finger and SCAN domain containing 30
1973	7896571		-5.1097653	0.8363	-7.99641	0.0000622	
1974	7893161		-4.4506494	-0.1386	-6.67867	0.000274	
1975	7892743		-4.3105245	-0.3535	-6.04747	0.0003769	
1976	7896604		-4.1561098	-0.5929	-5.4606	0.000536	
1977	7894123		-4.1414273	-0.6158	-5.55989	0.0005543	
1978	8161418		-4.0239707	-0.7996	-1.21213	0.0007249	
1979	7937977		-3.9991891	-0.8385	-0.99569	0.0007671	
1980	7893671		-3.9935711	-0.8473	-5.72545	0.000777	
1981	7893894		-3.8930902	-1.0057	-1.22885	0.0009775	
1982	7896014		-3.7780141	-1.1878	-4.01688	0.0012713	
1983	8023308		-3.7729802	-1.1958	-1.17539	0.001286	
1984	8124057		-3.7517631	-1.2294	-6.9413	0.0013498	
1985	7895713		-3.7150944	-1.2877	-4.65343	0.0014675	
1986	8122125		-3.714625	-1.2884	-1.10389	0.0014691	
1987	8037760		-3.6930453	-1.3227	-7.45878	0.0015432	
1988	7895857		-3.6831532	-1.3384	-0.76497	0.0015784	
1989	7896703		3.6742169	-1.3526	2.043324	0.0016109	
1990	8083407		-3.6737553	-1.3534	-5.86183	0.0016126	
1991	7894171		-3.6700688	-1.3592	-0.59367	0.0016262	
1992	8059339		3.6480997	-1.3942	0.79938	0.0017096	
1993	7894778		-3.6234732	-1.4334	-6.85888	0.0018082	
1994	7894321		3.6223654	-1.4351	1.029299	0.0018128	
1995	7950724		-3.600097	-1.4706	-6.19418	0.0019071	
1996	8139121		3.5746598	-1.5111	1.550797	0.0020207	
1997	8102781		-3.5214715	-1.5958	-1.1908	0.0022803	
1998	7894921		-3.512824	-1.6096	-2.75311	0.0023255	
1999	7927058		-3.5012355	-1.6281	-4.89585	0.0023875	
2000	8146788		-3.4911838	-1.6441	-1.0818	0.0024426	
2001	8043446		3.4517033	-1.707	2.454731	0.0026714	
2002	7896687		3.4424907	-1.7217	2.016261	0.0027277	
2003	8175662		-3.441201	-1.7237	-4.42095	0.0027357	
2004	8097011		3.433012	-1.7368	0.886882	0.0027869	
2005	8139031		-3.4303131	-1.7411	-0.96991	0.002804	
2006	7934613		-3.4123157	-1.7698	-5.32173	0.0029206	
2007	7960813		-3.4105812	-1.7725	-5.53078	0.0029321	
2008	7933088		-3.4074485	-1.7775	-2.03656	0.002953	
2009	8130027		-3.4039086	-1.7832	-1.73541	0.0029767	
2010	8070169		3.3864364	-1.811	1.023433	0.0030967	
2011	7892879		-3.3855997	-1.8123	-1.8818	0.0031026	
2012	8180239		-3.3769154	-1.8262	-1.0085	0.0031641	
2013	7892780		-3.3702035	-1.8368	-1.63436	0.0032124	
2014	7999384		-3.3636916	-1.8472	-1.7157	0.0032601	
2015	7893453		-3.361842	-1.8502	-0.61219	0.0032737	
2016	7906751		-3.3473595	-1.8732	-5.24961	0.0033825	
2017	7944829		-3.3343167	-1.894	-0.61017	0.0034836	
2018	7976146		-3.3280906	-1.9039	-3.3816	0.0035329	
2019	7923162		-3.3266415	-1.9062	-2.19962	0.0035444	
2020	7919049		3.3215856	-1.9143	0.701635	0.0035851	
2021	8124327		3.3092298	-1.9339	0.694444	0.0036864	
2022	7893627		-3.3039967	-1.9422	-0.92846	0.0037301	
2023	8043098		-3.2951513	-1.9563	-4.97665	0.0038052	
2024	8132988		-3.2932675	-1.9593	-1.86831	0.0038214	
2025	7894637		-3.2754734	-1.9876	-0.72943	0.0039776	
2026	7896625		-3.26694	-2.0012	-0.77388	0.0040547	
2027	7894081		-3.2629393	-2.0075	-1.24321	0.0040913	
2028	7998927		-3.2537915	-2.0221	-4.43621	0.0041764	
2029	8059714		-3.2427966	-2.0395	-1.72696	0.0042808	
2030	8058660		-3.2291904	-2.0611	-1.55394	0.0044137	
2031	8117286		3.228724	-2.0619	0.677791	0.0044183	
2032	8013259		3.2062829	-2.0975	0.651727	0.0046465	
2033	8136457		-3.1985174	-2.1098	-4.3616	0.0047281	
2034	7910636		-3.1687467	-2.157	-0.62224	0.0050542	
2035	8044611		3.1641873	-2.1643	1.428363	0.005106	
2036	7974027		-3.1526455	-2.1825	-4.83268	0.0052396	
2037	7894515		3.1502084	-2.1864	0.776662	0.0052682	
2038	7892752		3.1312558	-2.2164	1.546991	0.0054961	
2039	7896039		-3.1213526	-2.2321	-1.1336	0.005619	
2040	8111415		-3.1103335	-2.2495	-1.41941	0.0057589	
2041	7896695		3.1092711	-2.2512	1.958178	0.0057725	
2042	8043456		3.0974469	-2.2699	1.530565	0.0059268	

2043	7896524	3.0960108	-2.2721	0.974298	0.0059458
2044	8004802	3.0905318	-2.2808	0.89274	0.0060188
2045	8068218	3.088218	-2.2844	0.635039	0.0060499
2046	7984014	3.0760517	-2.3036	0.773048	0.006216
2047	7896422	-3.0754637	-2.3046	-0.5712	0.0062242
2048	8161854	-3.0667657	-2.3183	-1.3192	0.0063458
2049	8053715	3.0539662	-2.3385	1.608755	0.006529
2050	8139767	-3.0494263	-2.3456	-1.7791	0.0065952
2051	8083790	-3.0454132	-2.352	-0.92669	0.0066543
2052	8118098	3.0444394	-2.3535	0.517679	0.0066687
2053	8045810	-3.0436298	-2.3548	-1.7244	0.0066807
2054	8104070	-3.0434167	-2.3551	-0.87391	0.0066839
2055	8035080	3.0416571	-2.3579	1.288593	0.00671
2056	8091883	-3.0405571	-2.3596	-5.77511	0.0067264
2057	8101716	-3.0373748	-2.3646	-0.94772	0.0067741
2058	8081217	-3.0181839	-2.3948	-1.31661	0.0070688
2059	7896312	-3.0066737	-2.4129	-0.78574	0.0072515
2060	7911213	-2.9998906	-2.4236	-1.51365	0.0073612
2061	7894118	-2.9894976	-2.4399	-0.58247	0.0075325
2062	7896555	-2.9875795	-2.4429	-4.19248	0.0075646
2063	7896137	2.9832215	-2.4498	0.815674	0.0076379
2064	7895202	-2.9800376	-2.4548	-0.83106	0.0076918
2065	7917739	-2.9793924	-2.4558	-1.32367	0.0077028
2066	7895577	2.9769494	-2.4596	0.483284	0.0077445
2067	7969058	2.9751808	-2.4624	0.82243	0.0077749
2068	8139055	2.9673432	-2.4747	0.763312	0.0079107
2069	7923956	2.9458309	-2.5084	0.582888	0.0082954
2070	7893347	-2.937736	-2.5211	-5.83661	0.0084448
2071	7892874	-2.9324776	-2.5293	-2.26245	0.0085432
2072	7917695	-2.9068698	-2.5693	-0.98478	0.0090386
2073	8172195	-2.9030046	-2.5754	-6.35347	0.0091157
2074	7964260	-2.898287	-2.5827	-0.56534	0.0092107
2075	7894834	-2.8927869	-2.5913	-0.63179	0.0093227
2076	7919566	-2.877559	-2.615	-0.79043	0.0096395
2077	8088893	2.8679647	-2.63	0.565214	0.0098445
2078	8061092	-2.8619918	-2.6393	-0.82755	0.0099742
2079	8022426	2.8468609	-2.6628	1.070956	0.0103101
2080	7893132	-2.8459429	-2.6642	-0.96709	0.0103308
2081	8074192	-2.8429478	-2.6689	-1.78516	0.0103987
2082	7893568	-2.8339493	-2.6829	-0.80775	0.0106053
2083	7893179	-2.8324385	-2.6852	-5.29924	0.0106404
2084	7894190	-2.8318372	-2.6861	-3.85473	0.0106544
2085	8043426	2.8309442	-2.6875	1.09637	0.0106752
2086	8112996	-2.8276843	-2.6926	-5.84611	0.0107515
2087	7894635	-2.8167323	-2.7096	-0.96833	0.0110116
2088	8139786	-2.8148812	-2.7124	-4.90234	0.0110562
2089	7895831	-2.8104109	-2.7194	-0.66812	0.0111645
2090	8113467	-2.8102453	-2.7196	-0.52966	0.0111685
2091	8084605	-2.808267	-2.7227	-4.58184	0.0112168
2092	8176931	2.8070935	-2.7245	0.862153	0.0112456
2093	8177403	2.8070935	-2.7245	0.862153	0.0112456
2094	7894579	-2.8066274	-2.7252	-0.52849	0.011257
2095	8045559	2.8045444	-2.7284	0.786914	0.0113082
2096	8053662	-2.792903	-2.7464	-0.42352	0.0115987
2097	8121317	-2.7903467	-2.7504	-1.70012	0.0116634
2098	8176677	2.784445	-2.7595	0.799875	0.0118141
2099	8177178	2.784445	-2.7595	0.799875	0.0118141
2100	8023373	-2.7811776	-2.7646	-4.05762	0.0118984
2101	8002301	-2.775676	-2.7731	-0.79888	0.0120416
2102	8161863	-2.7695341	-2.7825	-0.8641	0.0122034
2103	7895101	-2.7691156	-2.7832	-0.58741	0.0122145
2104	7893815	-2.7688783	-2.7836	-0.50806	0.0122208
2105	8095035	2.7686496	-2.7839	4.899246	0.0122268
2106	7976552	-2.7680479	-2.7848	-3.10293	0.0122428
2107	7966568	-2.7668859	-2.7866	-1.1965	0.0122738
2108	8036881	-2.7624185	-2.7935	-0.8102	0.0123934
2109	7965671	-2.7612763	-2.7953	-0.84033	0.0124242
2110	8150590	-2.7571193	-2.8017	-3.82579	0.0125368
2111	8089873	-2.7552592	-2.8045	-2.68569	0.0125875
2112	7896724	2.7543196	-2.806	6.295223	0.0126132
2113	8168565	-2.7540308	-2.8064	-3.4946	0.0126211
2114	7892952	-2.748324	-2.8152	-0.64303	0.0127782
2115	8043423	2.7477471	-2.8161	2.826347	0.0127942

2116	7897995	-2.7431548	-2.8232	-3.30014	0.0129222
2117	7912589	-2.7431548	-2.8232	-3.30014	0.0129222
2118	8043443	2.7395667	-2.8287	1.365925	0.013023
2119	8180240	-2.7327554	-2.8392	-0.95431	0.0132166
2120	7971711	-2.7294724	-2.8442	-0.57533	0.0133108
2121	7957606	-2.7284436	-2.8458	-0.57363	0.0133405
2122	8002379	-2.7273003	-2.8476	-0.60273	0.0133735
2123	7893012	-2.7190503	-2.8602	-0.40632	0.0136142
2124	7894383	2.7186679	-2.8608	1.426351	0.0136255
2125	8040336	-2.7169353	-2.8635	-2.40663	0.0136766
2126	8020666	2.7137842	-2.8683	0.714878	0.0137701
2127	7896367	-2.7101409	-2.8739	-0.53059	0.0138789
2128	7905037	2.7076926	-2.8776	0.698836	0.0139524
2129	8123362	-2.7061549	-2.88	-0.712	0.0139988
2130	8173671	-2.701564	-2.887	-0.80989	0.0141382
2131	8151540	-2.6999952	-2.8894	-0.32334	0.0141862
2132	8073680	-2.6995531	-2.8901	-1.30002	0.0141997
2133	7996945	2.6961345	-2.8953	1.190631	0.0143048
2134	7892921	-2.6920509	-2.9016	-1.50479	0.0144313
2135	7896015	-2.6912304	-2.9028	-3.96632	0.0144569
2136	7895117	2.6878396	-2.908	0.898886	0.0145629
2137	7929130	-2.6788571	-2.9218	-0.6442	0.0148474
2138	8065120	-2.678066	-2.923	-0.87573	0.0148727
2139	7894618	2.6769563	-2.9247	0.998944	0.0149082
2140	7895039	-2.6767974	-2.9249	-0.45416	0.0149133
2141	7893552	2.6728098	-2.931	0.883284	0.0150418
2142	8042460	-2.6690137	-2.9368	-1.58369	0.0151652
2143	7893864	-2.663212	-2.9456	-3.37551	0.0153555
2144	8041711	-2.6599243	-2.9506	-0.92969	0.0154644
2145	7894657	-2.6586493	-2.9526	-0.59236	0.0155068
2146	8067199	-2.6565258	-2.9558	-0.68854	0.0155777
2147	7896719	2.6513246	-2.9637	1.279429	0.0157526
2148	8044572	-2.6499413	-2.9659	-0.94063	0.0157994
2149	8122856	-2.6434677	-2.9757	-2.48667	0.0160204
2150	7895485	-2.6428315	-2.9767	-5.27535	0.0160422
2151	7895404	-2.6409488	-2.9795	-1.31492	0.0161071
2152	7895541	-2.6408815	-2.9796	-1.72965	0.0161095
2153	8102783	2.6350113	-2.9886	0.687414	0.0163134
2154	7894080	-2.631878	-2.9933	-0.6668	0.0164233
2155	7942910	-2.6316636	-2.9936	-0.62685	0.0164308
2156	7896108	-2.6313001	-2.9942	-0.61029	0.0164436
2157	8114191	-2.6288196	-2.998	-2.84748	0.0165312
2158	7942822	2.6281523	-2.999	1.848382	0.0165548
2159	7979129	2.6227912	-3.0071	0.668077	0.0167459
2160	7892946	-2.6174721	-3.0152	-0.8401	0.0169375
2161	7894136	-2.6174323	-3.0152	-0.52363	0.0169389
2162	8078134	2.6167053	-3.0163	5.948119	0.0169653
2163	7893400	-2.6161396	-3.0172	-1.19944	0.0169858
2164	8124192	2.5989591	-3.0432	0.823653	0.0176207
2165	8046755	2.5948671	-3.0494	0.630338	0.0177752
2166	8121390	-2.5944479	-3.05	-3.07749	0.0177911
2167	7919564	2.5927959	-3.0525	0.623986	0.0178539
2168	7894185	-2.5927356	-3.0526	-0.55631	0.0178562
2169	8143205	2.592165	-3.0535	0.671111	0.0178779
2170	8058159	-2.5876142	-3.0604	-0.77161	0.0180522
2171	8124636	2.5874308	-3.0606	0.825488	0.0180592
2172	7932508	-2.5861298	-3.0626	-3.61522	0.0181094
2173	7893312	2.5856538	-3.0633	5.070371	0.0181277
2174	8029375	-2.583795	-3.0661	-1.10729	0.0181997
2175	7960434	2.5830893	-3.0672	0.560118	0.018227
2176	8176373	2.5828147	-3.0676	0.434256	0.0182377
2177	8173627	-2.5757004	-3.0783	-0.76985	0.018516
2178	7894166	-2.5756353	-3.0784	-5.49431	0.0185186
2179	8045319	-2.5742674	-3.0805	-1.669	0.0185726
2180	8055234	-2.5742674	-3.0805	-1.669	0.0185726
2181	7911110	-2.5736371	-3.0814	-1.13648	0.0185975
2182	7894936	-2.5723794	-3.0833	-2.85866	0.0186473
2183	7971561	2.5696944	-3.0874	0.541422	0.0187541
2184	7997228	2.5676236	-3.0905	0.83249	0.0188369
2185	7925902	2.5633533	-3.0969	0.562133	0.0190086
2186	7895015	-2.5619168	-3.0991	-0.9684	0.0190667
2187	8082795	-2.5592104	-3.1032	-1.08149	0.0191766
2188	7893994	-2.5568907	-3.1067	-1.82074	0.0192713

2189	7894173	-2.556539	-3.1072	-0.72928	0.0192857
2190	7895766	2.5540499	-3.1109	0.370062	0.0193879
2191	8081113	-2.5487859	-3.1188	-0.55076	0.0196057
2192	7916998	-2.5453681	-3.124	-2.94599	0.0197483
2193	8150163	-2.5452189	-3.1242	-1.31029	0.0197545
2194	7895657	-2.5448443	-3.1248	-0.367	0.0197702
2195	7894463	-2.5447408	-3.1249	-0.84543	0.0197746
2196	7895564	-2.5440228	-3.126	-4.22188	0.0198047
2197	8148206	-2.5429293	-3.1276	-4.41231	0.0198506
2198	7893688	-2.538231	-3.1347	-2.34359	0.0200493
2199	7896698	2.5337884	-3.1413	1.045837	0.0202388
2200	7893197	-2.5323457	-3.1435	-4.62278	0.0203007
2201	8053364	2.5305704	-3.1462	0.648143	0.0203771
2202	7925432	2.5297922	-3.1473	5.580358	0.0204107
2203	7893667	-2.5297293	-3.1474	-2.41392	0.0204134
2204	8043476	2.5265647	-3.1522	1.978085	0.0205506
2205	8005630	2.5238089	-3.1563	1.145031	0.0206707
2206	7910913	-2.5211242	-3.1603	-1.75906	0.0207884
2207	8017141	2.5201392	-3.1618	1.61065	0.0208317
2208	7894360	-2.5179839	-3.165	-0.71294	0.0209268
2209	7948371	2.515455	-3.1688	0.741588	0.0210389
2210	8131612	-2.5115068	-3.1747	-0.71707	0.0212151
2211	7946426	-2.5111694	-3.1752	-0.6713	0.0212302
2212	7895529	-2.506766	-3.1818	-4.53313	0.0214284
2213	8014142	-2.5046823	-3.1849	-1.08226	0.0215228
2214	8053690	2.5034346	-3.1867	1.792684	0.0215795
2215	7896489	2.4999436	-3.1919	0.8905	0.021739
2216	8136656	-2.4995124	-3.1926	-1.86342	0.0217588
2217	7896119	-2.4985401	-3.194	-2.5525	0.0218034
2218	8019561	-2.4962834	-3.1974	-3.58204	0.0219073
2219	8088546	-2.4939529	-3.2009	-1.46146	0.0220152
2220	8096897	-2.493767	-3.2011	-0.5896	0.0220238
2221	8168674	-2.4936419	-3.2013	-1.48629	0.0220296
2222	8071658	2.4912287	-3.2049	1.884444	0.0221419
2223	8046512	-2.4909956	-3.2053	-0.9599	0.0221527
2224	7893526	2.4899613	-3.2068	1.832181	0.022201
2225	7974164	-2.4878048	-3.21	-1.57978	0.0223021
2226	8152090	-2.4870677	-3.2111	-0.82901	0.0223367
2227	7954102	2.485488	-3.2135	1.481555	0.0224111
2228	8103458	-2.4851528	-3.214	-3.42643	0.0224269
2229	7993798	-2.4836569	-3.2162	-3.5866	0.0224976
2230	8042115	-2.4828688	-3.2174	-1.35533	0.0225349
2231	8147564	-2.4826049	-3.2178	-4.4169	0.0225474
2232	7896498	-2.4822639	-3.2183	-0.42931	0.0225636
2233	7893371	-2.4819126	-3.2188	-0.39014	0.0225803
2234	8058668	-2.4818771	-3.2188	-1.17128	0.022582
2235	8106401	2.4808133	-3.2204	0.577021	0.0226326
2236	8141303	2.4729825	-3.2321	0.868383	0.0230082
2237	8084215	2.4695895	-3.2371	0.506262	0.0231727
2238	7893174	-2.4640656	-3.2453	-1.74819	0.023443
2239	7942525	2.4627683	-3.2472	0.46435	0.0235069
2240	8089928	2.4585418	-3.2535	0.585853	0.0237162
2241	8136159	-2.4578951	-3.2544	-1.29635	0.0237484
2242	7901383	-2.456715	-3.2562	-1.28261	0.0238072
2243	8054090	2.4532484	-3.2613	0.574537	0.0239808
2244	7896713	2.452327	-3.2627	0.462984	0.0240272
2245	8155024	2.4473645	-3.27	0.541218	0.0242782
2246	7896473	-2.4473525	-3.27	-3.3913	0.0242788
2247	8137470	2.4471288	-3.2704	0.730713	0.0242902
2248	8174977	2.4457638	-3.2724	0.558721	0.0243597
2249	8124036	-2.442511	-3.2772	-2.72677	0.0245261
2250	8158511	2.441831	-3.2782	0.51713	0.024561
2251	7896030	-2.4405488	-3.2801	-0.41129	0.024627
2252	8146837	2.4403358	-3.2804	0.910432	0.024638
2253	7896716	2.4400317	-3.2809	1.021214	0.0246536
2254	7895911	-2.4387853	-3.2827	-0.41837	0.024718
2255	7894294	-2.4372648	-3.285	-0.41684	0.0247967
2256	8002018	-2.4355997	-3.2874	-0.63464	0.0248832
2257	8001102	2.4313284	-3.2937	1.540279	0.0251063
2258	7895982	-2.4307634	-3.2945	-0.38329	0.0251359
2259	7894211	-2.4302699	-3.2953	-0.52344	0.0251619
2260	8146754	-2.4298204	-3.2959	-0.64472	0.0251855
2261	7943517	-2.4285526	-3.2978	-1.02703	0.0252523

2262	8089230	2.428318	-3.2982	0.456187	0.0252646
2263	7986567	2.426768	-3.3004	0.517216	0.0253465
2264	7986633	2.426768	-3.3004	0.517216	0.0253465
2265	7894815	-2.4250923	-3.3029	-0.71559	0.0254354
2266	7892605	-2.4242199	-3.3042	-0.54861	0.0254817
2267	8061112	2.4233645	-3.3054	0.553504	0.0255273
2268	7896653	-2.4219292	-3.3076	-0.51394	0.0256038
2269	7895843	-2.4215118	-3.3082	-1.19169	0.0256261
2270	7990652	2.4213075	-3.3085	0.724354	0.0256371
2271	8132413	-2.4204001	-3.3098	-2.96228	0.0256856
2272	8085529	-2.4195187	-3.3111	-0.97792	0.0257329
2273	7932304	2.4192341	-3.3115	5.579525	0.0257482
2274	7894157	-2.4190085	-3.3119	-4.39002	0.0257603
2275	7895242	-2.4172014	-3.3145	-3.33167	0.0258576
2276	7894345	-2.4171654	-3.3146	-3.13683	0.0258595
2277	7893216	-2.4119149	-3.3223	-0.65622	0.0261441
2278	8045347	-2.4116535	-3.3227	-3.27002	0.0261583
2279	8109526	2.4115612	-3.3228	0.588772	0.0261633
2280	8151250	-2.4097055	-3.3255	-0.7914	0.0262647
2281	7894492	2.4096667	-3.3256	0.470388	0.0262668
2282	7919390	-2.4087328	-3.327	-1.08585	0.0263179
2283	8083848	-2.4084437	-3.3274	-1.15683	0.0263338
2284	7893563	-2.4079422	-3.3281	-1.27284	0.0263613
2285	8072411	2.4075229	-3.3288	0.520005	0.0263843
2286	7893806	2.4064536	-3.3303	0.598339	0.0264431
2287	7893772	-2.4039024	-3.3341	-0.95352	0.0265839
2288	8019958	-2.4035089	-3.3346	-2.92834	0.0266057
2289	8009299	-2.4021594	-3.3366	-0.83574	0.0266805
2290	7952675	2.3995584	-3.3404	0.563223	0.0268253
2291	7895896	-2.3986575	-3.3418	-3.15121	0.0268756
2292	7894351	2.3958676	-3.3459	0.581768	0.027032
2293	7895272	-2.3958042	-3.3459	-0.39837	0.0270355
2294	7895808	2.3919083	-3.3517	0.456641	0.0272553
2295	7956987	-2.3919062	-3.3517	-0.46855	0.0272554
2296	7896221	-2.391836	-3.3518	-0.59706	0.0272594
2297	8005759	-2.3876508	-3.3579	-0.75606	0.0274974
2298	8103873	2.3852764	-3.3614	1.17771	0.0276333
2299	8099756	-2.3849126	-3.3619	-0.41671	0.0276542
2300	8146305	-2.3839805	-3.3633	-0.47397	0.0277077
2301	8047765	-2.3815201	-3.3669	-0.58656	0.0278495
2302	8102726	-2.3810946	-3.3675	-4.89175	0.0278741
2303	8121416	-2.3808037	-3.3679	-1.88864	0.0278909
2304	7942989	-2.3805482	-3.3683	-0.67689	0.0279057
2305	7895331	2.3803841	-3.3685	1.709153	0.0279152
2306	7893963	-2.3802517	-3.3687	-3.65767	0.0279229
2307	7896715	2.3796619	-3.3696	1.740888	0.0279571
2308	7892770	2.3779036	-3.3721	0.608488	0.0280592
2309	8107090	2.3750374	-3.3763	0.318382	0.0282264
2310	7895442	-2.3740205	-3.3778	-2.02224	0.028286
2311	7895259	2.3735115	-3.3786	0.762917	0.0283158
2312	8064926	-2.3714613	-3.3816	-2.3446	0.0284364
2313	7931926	2.3687007	-3.3856	0.636081	0.0285994
2314	8174251	-2.366929	-3.3882	-0.55297	0.0287045
2315	7892846	-2.3652309	-3.3906	-0.3656	0.0288056
2316	8130209	-2.3648789	-3.3912	-4.10872	0.0288266
2317	8106528	2.3603503	-3.3978	0.454514	0.029098
2318	7896563	2.3601462	-3.398	0.619242	0.0291102
2319	8166872	2.3594926	-3.399	0.923984	0.0291496
2320	7895945	-2.3590038	-3.3997	-0.65515	0.0291791
2321	8043470	2.3588992	-3.3999	2.1296	0.0291854
2322	7907968	-2.3583131	-3.4007	-4.41668	0.0292208
2323	8117032	-2.3570005	-3.4026	-1.40709	0.0293002
2324	8100576	2.3537769	-3.4073	5.295926	0.0294961
2325	8092220	-2.3536676	-3.4075	-0.584	0.0295027
2326	7893236	2.3534674	-3.4078	0.38289	0.0295149
2327	8036025	-2.349487	-3.4136	-1.33237	0.0297586
2328	7894942	-2.3489603	-3.4143	-4.73626	0.029791
2329	7894727	-2.348942	-3.4144	-0.56416	0.0297921
2330	7948042	2.3462346	-3.4183	0.686109	0.0299591
2331	7895551	-2.3458342	-3.4189	-0.40404	0.0299838
2332	7892740	2.3431072	-3.4228	1.94812	0.030153
2333	7894212	2.3426053	-3.4236	0.588931	0.0301843
2334	7896181	2.3421667	-3.4242	0.888727	0.0302116

2335	8123763	2.341685	-3.4249	0.530692	0.0302416
2336	7895983	-2.3408339	-3.4261	-3.08068	0.0302948
2337	7892959	2.3406802	-3.4263	0.450697	0.0303044
2338	7895914	2.3377053	-3.4307	1.268048	0.0304908
2339	7894096	-2.3375363	-3.4309	-0.65025	0.0305014
2340	8105645	-2.3350922	-3.4345	-4.97901	0.0306554
2341	8044743	2.330395	-3.4413	1.361758	0.0309535
2342	8081818	2.329442	-3.4426	0.483353	0.0310143
2343	8035859	2.3289061	-3.4434	0.549144	0.0310485
2344	7917180	-2.3274202	-3.4456	-1.39739	0.0311436
2345	7918755	2.324981	-3.4491	0.726633	0.0313003
2346	8111627	-2.3237361	-3.4509	-0.67521	0.0313806
2347	8127656	-2.3227452	-3.4523	-3.72561	0.0314446
2348	7892983	-2.3226828	-3.4524	-0.37202	0.0314486
2349	7894113	-2.3219906	-3.4534	-4.08207	0.0314934
2350	7895810	2.3216807	-3.4539	0.627437	0.0315135
2351	8097305	2.3208591	-3.4551	1.01164	0.0315668
2352	8165860	-2.3189344	-3.4578	-0.60931	0.0316919
2353	7895308	2.3155402	-3.4627	0.498694	0.0319138
2354	7893313	-2.3147071	-3.4639	-0.49628	0.0319684
2355	7895199	2.3132733	-3.466	4.152251	0.0320627
2356	7895691	2.3128664	-3.4666	0.708266	0.0320895
2357	8021824	2.3115259	-3.4685	0.910749	0.032178
2358	7895085	2.3106829	-3.4698	0.786913	0.0322337
2359	7895122	-2.3081069	-3.4735	-0.37813	0.0324046
2360	8135271	-2.3077933	-3.4739	-0.67303	0.0324254
2361	7914498	-2.3062922	-3.4761	-0.53687	0.0325254
2362	8049197	-2.3058052	-3.4768	-1.51789	0.032558
2363	7966425	-2.3025065	-3.4815	-0.45463	0.0327789
2364	8020971	-2.3019303	-3.4824	-0.47785	0.0328177
2365	7986627	-2.3016798	-3.4827	-4.84962	0.0328346
2366	8053718	2.301123	-3.4835	5.789918	0.0328721
2367	7892772	-2.2995518	-3.4858	-0.91433	0.0329781
2368	7895093	2.2960591	-3.4908	0.977975	0.0332149
2369	8169156	-2.2958174	-3.4912	-0.54711	0.0332314
2370	7895008	-2.2950872	-3.4922	-1.25342	0.0332811
2371	7917050	-2.294397	-3.4932	-0.78443	0.0333282
2372	8021368	2.2930641	-3.4951	0.694815	0.0334192
2373	7930629	-2.2914017	-3.4975	-3.1357	0.0335332
2374	7893984	-2.2905185	-3.4988	-3.24955	0.0335938
2375	7981317	2.2899405	-3.4996	0.399395	0.0336336
2376	8043022	2.2892006	-3.5007	0.956442	0.0336845
2377	7984255	-2.2887387	-3.5013	-5.08298	0.0337164
2378	8045275	-2.2879924	-3.5024	-1.4744	0.0337679
2379	7895574	-2.2874808	-3.5032	-0.46347	0.0338033
2380	7895823	-2.2852193	-3.5064	-3.91493	0.03396
2381	7895532	-2.2843275	-3.5077	-0.74771	0.0340219
2382	8156261	2.2834032	-3.509	4.960782	0.0340863
2383	8128258	2.2833469	-3.5091	0.426908	0.0340902
2384	7983938	-2.2820908	-3.5109	-0.66678	0.0341778
2385	7947421	-2.2800036	-3.5139	-0.72577	0.0343239
2386	8145622	2.2798622	-3.5141	0.571398	0.0343338
2387	8101061	-2.2791115	-3.5152	-0.45727	0.0343865
2388	8079149	2.2789086	-3.5155	0.514483	0.0344008
2389	7894013	-2.276149	-3.5194	-0.85532	0.0345952
2390	7928514	-2.2759936	-3.5196	-0.50108	0.0346062
2391	7894167	-2.2758517	-3.5198	-0.85416	0.0346162
2392	8151445	-2.2756643	-3.5201	-3.63932	0.0346294
2393	8117565	2.2753659	-3.5205	0.535062	0.0346506
2394	7894010	-2.2746989	-3.5215	-3.16918	0.0346978
2395	8043441	2.27326	-3.5235	1.82997	0.0347998
2396	8055305	2.2708242	-3.527	0.65083	0.0349732
2397	7896066	-2.2705814	-3.5274	-3.45928	0.0349905
2398	7896505	-2.2694789	-3.529	-3.62133	0.0350693
2399	8091444	2.2683438	-3.5306	0.505951	0.0351506
2400	8034905	-2.2679079	-3.5312	-0.56762	0.0351819
2401	8050906	-2.2663497	-3.5334	-0.58566	0.0352938
2402	8116992	2.2638009	-3.5371	1.159428	0.0354776
2403	7906993	2.2622793	-3.5392	0.376981	0.0355878
2404	8043572	-2.2621379	-3.5395	-0.39345	0.035598
2405	8096457	2.2605899	-3.5417	0.492975	0.0357105
2406	7894471	-2.2601844	-3.5422	-0.44885	0.03574
2407	8102592	-2.2560911	-3.5481	-0.7675	0.036039

2408	7895534	-2.2548394	-3.5499	-0.84135	0.036131
2409	7910674	2.2522375	-3.5536	5.19529	0.0363227
2410	7934840	2.2475891	-3.5602	0.663485	0.0366676
2411	7953979	2.2474624	-3.5604	1.554469	0.0366771
2412	7893723	-2.2466686	-3.5615	-0.96157	0.0367363
2413	8036989	2.2461833	-3.5622	0.515365	0.0367725
2414	8168884	-2.2450548	-3.5638	-2.79517	0.0368569
2415	7985200	-2.2448571	-3.5641	-0.49633	0.0368718
2416	8145134	2.2433769	-3.5662	0.91917	0.0369828
2417	8090637	2.2418356	-3.5684	0.344663	0.0370987
2418	7896442	-2.2418346	-3.5684	-0.65531	0.0370988
2419	7892675	2.241825	-3.5684	0.903076	0.0370995
2420	8120269	-2.241027	-3.5695	-0.68269	0.0371597
2421	7896696	2.2406784	-3.57	0.418022	0.037186
2422	8113614	-2.2395692	-3.5716	-0.59253	0.0372698
2423	8136291	2.2388126	-3.5727	5.493066	0.0373271
2424	8055592	2.2374737	-3.5746	0.540014	0.0374286
2425	7893779	-2.2372007	-3.575	-0.34077	0.0374494
2426	8000882	2.2368144	-3.5755	0.491455	0.0374787
2427	7945950	-2.2367975	-3.5756	-1.28436	0.03748
2428	7899375	-2.2365805	-3.5759	-2.68711	0.0374965
2429	7893724	-2.2360003	-3.5767	-0.78201	0.0375407
2430	7893487	-2.2356533	-3.5772	-0.79837	0.0375671
2431	8083939	2.2344476	-3.5789	4.269085	0.0376591
2432	8134403	-2.2335076	-3.5802	-3.15343	0.037731
2433	8122240	-2.2328705	-3.5811	-4.73517	0.0377797
2434	7961887	2.2323254	-3.5819	0.355948	0.0378215
2435	8020025	-2.2271826	-3.5892	-0.6166	0.0382177
2436	8124194	2.2266848	-3.5899	0.665653	0.0382563
2437	7930915	2.2248508	-3.5925	0.665037	0.0383986
2438	8180220	-2.2238545	-3.5939	-0.95362	0.0384762
2439	8084122	2.2212269	-3.5976	0.586277	0.0386813
2440	7955074	-2.2211234	-3.5978	-4.71944	0.0386894
2441	8092199	-2.2198782	-3.5995	-1.51494	0.038787
2442	7925246	-2.2196244	-3.5999	-3.24867	0.038807
2443	8152211	-2.2186075	-3.6013	-0.71982	0.0388869
2444	8083592	-2.2172147	-3.6033	-2.81591	0.0389966
2445	7896694	2.2151043	-3.6063	1.050642	0.0391633
2446	8052056	-2.214365	-3.6073	-2.94923	0.0392219
2447	8176415	-2.2141966	-3.6076	-0.60775	0.0392352
2448	8145529	2.2139468	-3.6079	0.580784	0.039255
2449	7894945	2.2127638	-3.6096	0.510405	0.039349
2450	8105189	-2.2114868	-3.6114	-0.60182	0.0394507
2451	7896701	2.2109243	-3.6122	1.225044	0.0394955
2452	8095037	-2.2079096	-3.6165	-3.70838	0.0397367
2453	8113443	2.2077076	-3.6168	0.470324	0.0397529
2454	7968732	-2.2076649	-3.6168	-0.48248	0.0397563
2455	7895589	2.2076086	-3.6169	0.438721	0.0397609
2456	7988685	2.2072295	-3.6174	0.448312	0.0397913
2457	7893647	-2.2064146	-3.6186	-0.348	0.0398568
2458	8085052	-2.203575	-3.6226	-0.80637	0.0400859
2459	7895159	-2.2015788	-3.6254	-2.91225	0.0402476
2460	8112174	-2.2012898	-3.6258	-0.40488	0.0402711
2461	8161568	2.200515	-3.6269	1.692231	0.040334
2462	8148329	2.1993798	-3.6285	0.425721	0.0404265
2463	7896490	-2.1979863	-3.6305	-0.36563	0.0405402
2464	7895322	-2.1975695	-3.6311	-0.4725	0.0405743
2465	7893608	-2.1970344	-3.6318	-0.5064	0.040618
2466	7910672	2.1947844	-3.635	4.805167	0.0408026
2467	7894304	2.1945463	-3.6353	0.474214	0.0408222
2468	7892733	-2.1941216	-3.6359	-0.99922	0.0408571
2469	8009513	-2.1932225	-3.6372	-0.46302	0.0409312
2470	8170418	-2.1932067	-3.6372	-0.43731	0.0409325
2471	7962242	-2.1923311	-3.6384	-0.66421	0.0410047
2472	7896106	-2.191359	-3.6398	-1.74134	0.0410851
2473	8111212	2.1900591	-3.6416	0.669256	0.0411927
2474	8161580	2.1898031	-3.642	1.684257	0.0412139
2475	7896109	-2.1893841	-3.6426	-4.75762	0.0412487
2476	7971913	-2.1877048	-3.6449	-4.35931	0.0413883
2477	7894436	-2.1872183	-3.6456	-0.68121	0.0414289
2478	8003857	-2.1866436	-3.6464	-0.74511	0.0414768
2479	7894762	-2.1862101	-3.647	-0.71974	0.041513
2480	7893173	-2.1854202	-3.6481	-0.34643	0.041579

2481	8106767	-2.1828632	-3.6517	-0.95381	0.0417933
2482	7895636	2.1819128	-3.6531	5.513379	0.0418733
2483	7918757	-2.181028	-3.6543	-0.6958	0.0419478
2484	7938299	-2.1797754	-3.6561	-3.07176	0.0420535
2485	7895949	2.178012	-3.6585	0.68198	0.0422028
2486	7895491	-2.177264	-3.6596	-0.89576	0.0422662
2487	7895538	2.1770967	-3.6598	0.691606	0.0422804
2488	7894402	-2.1755718	-3.662	-0.3367	0.0424101
2489	7895861	-2.1750956	-3.6626	-4.2987	0.0424507
2490	7911349	-2.1746469	-3.6633	-0.78974	0.0424889
2491	8168998	-2.1742613	-3.6638	-0.51794	0.0425218
2492	7934095	-2.1722097	-3.6667	-1.58546	0.0426973
2493	7893468	-2.1713765	-3.6678	-1.23782	0.0427687
2494	7892679	-2.1703919	-3.6692	-1.09736	0.0428533
2495	7895280	2.1692334	-3.6708	0.376846	0.042953
2496	7895381	-2.1671418	-3.6738	-1.01681	0.0431335
2497	8142017	2.1670679	-3.6739	0.414667	0.0431399
2498	7893716	-2.1652998	-3.6763	-1.71338	0.0432931
2499	7896101	-2.1629137	-3.6797	-0.4267	0.0435005
2500	7893293	2.1614207	-3.6818	4.399878	0.0436308
2501	7895048	2.1603914	-3.6832	0.780093	0.0437209
2502	8167784	2.1592564	-3.6848	0.733056	0.0438203
2503	8129039	-2.1564685	-3.6887	-1.44993	0.0440656
2504	8019937	2.1539794	-3.6921	0.362394	0.0442855
2505	8009524	2.1529135	-3.6936	0.670128	0.0443801
2506	7893125	2.1525382	-3.6942	0.571335	0.0444134
2507	7893754	-2.1518117	-3.6952	-0.86063	0.0444779
2508	7927031	-2.1517948	-3.6952	-2.34442	0.0444794
2509	7906465	2.1512392	-3.696	0.624527	0.0445289
2510	8058195	-2.1506236	-3.6968	-5.21381	0.0445837
2511	8070900	2.1505719	-3.6969	0.452995	0.0445883
2512	8067981	-2.149835	-3.6979	-3.01433	0.0446541
2513	7892704	-2.1489985	-3.6991	-0.35882	0.0447288
2514	8168676	-2.148522	-3.6997	-0.47601	0.0447714
2515	8147371	-2.14835	-3.7	-2.52334	0.0447868
2516	7895129	-2.1470082	-3.7019	-2.99415	0.044907
2517	7896528	-2.1459455	-3.7033	-0.38404	0.0450024
2518	7960514	-2.1457867	-3.7036	-3.77485	0.0450167
2519	7894907	-2.1440799	-3.7059	-0.58337	0.0451704
2520	7893481	-2.1439532	-3.7061	-2.14565	0.0451818
2521	7894617	-2.1425541	-3.708	-0.57007	0.0453082
2522	7950893	-2.1424799	-3.7082	-2.2641	0.0453149
2523	8003846	2.1419235	-3.7089	0.388715	0.0453653
2524	8052932	2.1414415	-3.7096	0.803746	0.0454089
2525	8130183	2.139566	-3.7122	0.747932	0.0455792
2526	7970804	-2.1390509	-3.7129	-0.48719	0.0456261
2527	8048761	-2.1376705	-3.7148	-0.77797	0.0457519
2528	8047559	2.1376386	-3.7149	4.656047	0.0457548
2529	8107671	2.1373384	-3.7153	0.620523	0.0457822
2530	7895508	2.135246	-3.7182	0.457029	0.0459736
2531	8066027	2.1335171	-3.7206	4.774872	0.0461323
2532	7901374	-2.1334894	-3.7206	-2.93291	0.0461348
2533	8101990	-2.1327374	-3.7217	-0.70031	0.046204
2534	7975309	2.1300885	-3.7254	0.98262	0.0464485
2535	8130403	2.1289962	-3.7269	0.293649	0.0465496
2536	8150149	-2.1282179	-3.7279	-0.47998	0.0466218
2537	8085912	-2.1274583	-3.729	-4.12686	0.0466924
2538	7903113	-2.1268058	-3.7299	-2.75322	0.0467531
2539	7952223	-2.1268025	-3.7299	-0.5976	0.0467534
2540	8093974	-2.1243928	-3.7332	-0.61616	0.0469781
2541	7896094	2.1228971	-3.7353	0.487903	0.0471181
2542	8124924	-2.1228093	-3.7354	-0.37905	0.0471263
2543	7893067	-2.1215718	-3.7372	-0.89762	0.0472424
2544	7896664	-2.1189394	-3.7408	-0.38068	0.0474903
2545	8055909	-2.1186456	-3.7412	-0.99124	0.0475181
2546	7893513	-2.1183446	-3.7416	-0.31547	0.0475465
2547	7896714	2.1158988	-3.745	1.503515	0.0477781
2548	8134550	-2.1149868	-3.7463	-0.53064	0.0478648
2549	8131512	2.1143112	-3.7472	0.975397	0.047929
2550	7898373	-2.1141375	-3.7474	-1.00922	0.0479456
2551	8055641	2.1133026	-3.7486	0.665714	0.0480251
2552	7900633	-2.1107795	-3.7521	-4.43797	0.0482663
2553	7894591	-2.1079934	-3.7559	-2.43576	0.0485338

2554	7910096	2.1078508	-3.7561	0.580823	0.0485475
2555	8171489	-2.1075671	-3.7565	-3.79915	0.0485749
2556	7950532	-2.106334	-3.7582	-3.89534	0.0486938
2557	7895962	-2.1052674	-3.7597	-1.0455	0.0487969
2558	7896187	-2.1044285	-3.7608	-4.70373	0.0488781
2559	8067197	2.1041601	-3.7612	0.533039	0.0489041
2560	7981773	2.1030251	-3.7627	0.597636	0.0490143
2561	7939998	2.102735	-3.7631	0.382322	0.0490424
2562	7899071	2.1027252	-3.7632	0.796351	0.0490434
2563	7893030	2.1024467	-3.7635	0.796873	0.0490705
2564	7894742	2.1018573	-3.7644	0.701968	0.0491278
2565	7895976	-2.1003483	-3.7664	-0.59611	0.0492749
2566	7895341	-2.1000538	-3.7668	-3.76815	0.0493036
2567	7949954	-2.1000249	-3.7669	-2.03301	0.0493065
2568	7895464	-2.0998666	-3.7671	-0.56906	0.0493219
2569	8154719	-2.09876	-3.7686	-1.98998	0.0494301
2570	7896468	-2.0984803	-3.769	-0.40313	0.0494575
2571	8111214	2.0981067	-3.7695	0.417777	0.0494941
2572	7895424	-2.0971538	-3.7708	-0.92762	0.0495876
2573	8133854	2.0971222	-3.7709	5.204177	0.0495907
2574	8022745	2.0960733	-3.7723	0.398289	0.0496938
2575	7973869	2.0958311	-3.7726	1.701581	0.0497176

Supplementary Table 2. Differentially expressed gene list of TNBC cell lines (BT549, HS578T, and MDA-MB-231) versus non-TNBC cell lines (MCF7 and T47D).

#	ID	Gene.symbol	TNBC group (tested)	non-TNBC group (base line)	logFC	P.Value	Gene.title
1	209459_s_at	ABAT	26.491167	36.54245	-5.884911	5.55E-16	4-aminobutyrate aminotransferase
2	209460_at	ABAT	21.396832	24.901831	-4.7562425	1.53E-13	4-aminobutyrate aminotransferase
3	206527_at	ABAT	12.272142	13.086629	-1.2167816	1.45E-09	4-aminobutyrate aminotransferase
4	215465_at	ABCA12	33.241545	68.979819	-5.4222065	4.60E-20	ATP binding cassette subfamily A member 12
5	230913_at	ABCG1	10.512109	11.54248	-2.3117885	8.01E-09	ATP binding cassette subfamily G member 1
6	208636_at	ACTN1	11.197986	-12.124168	1.9801712	4.12E-09	actinin alpha 1
7	213102_at	ACTR3	13.795153	-14.570094	1.5931459	3.27E-10	ARP3 actin related protein 3 homolog
8	209765_at	ADAM19	10.706551	-11.704903	1.8636232	6.63E-09	ADAM metallopeptidase domain 19
9	237411_at	ADAMTS6	15.344434	-16.241309	4.4511655	7.10E-11	ADAM metallopeptidase with thrombospondin type 1 motif 6
10	209195_s_at	ADCY6	14.359695	15.1589	-1.5336519	1.88E-10	adenylate cyclase 6
11	223582_at	ADGRV1	14.164987	14.953307	-1.8078524	2.27E-10	adhesion G protein-coupled receptor V1
12	205891_at	ADORA2B	15.25965	-16.145219	5.0939892	7.72E-11	adenosine A2b receptor
13	225114_at	AGPS	11.110436	-12.04854	4.7619854	4.48E-09	alkylglycerone phosphate synthase
14	225108_at	AGPS	11.065719	-12.010071	3.0934464	4.68E-09	alkylglycerone phosphate synthase
15	202820_at	AHR	11.654044	-12.524866	2.9984632	2.64E-09	aryl hydrocarbon receptor
16	1553734_at	AK7	12.595471	13.389421	-3.954627	1.06E-09	adenylate kinase 7
17	201272_at	AKR1B1	18.830324	-20.739883	5.2674114	2.16E-12	aldo-keto reductase family 1 member B
18	212607_at	AKT3	15.720464	-16.674346	4.1472436	4.89E-11	AKT serine/threonine kinase 3
19	222880_at	AKT3	15.627991	-16.566802	3.6666826	5.36E-11	AKT serine/threonine kinase 3
20	212609_s_at	AKT3	15.499833	-16.418897	3.7377981	6.09E-11	AKT serine/threonine kinase 3
21	209645_s_at	ALDH1B1	12.350287	-13.159236	1.820933	1.34E-09	aldehyde dehydrogenase 1 family member B1
22	206385_s_at	ANK3	19.162678	21.232536	-4.8054182	1.54E-12	ankyrin 3, node of Ranvier (ankyrin G)
23	228573_at	ANTXR2	13.562188	-14.333493	3.4331134	4.10E-10	anthrax toxin receptor 2
24	225524_at	ANTXR2	13.083643	-13.85872	4.5503851	6.56E-10	anthrax toxin receptor 2
25	201012_at	ANXA1	16.515151	-17.627897	7.062545	2.22E-11	annexin A1
26	200782_at	ANXA5	17.687904	-19.137927	1.2185257	6.86E-12	annexin A5
27	211712_s_at	ANXA9	12.397109	13.202914	-4.5001464	1.28E-09	annexin A9
28	210085_s_at	ANXA9	11.413583	12.312172	-3.7942773	3.34E-09	annexin A9
29	211975_at	ARFGAP2	11.485046	12.375049	-1.0737957	3.11E-09	ADP ribosylation factor GTPase activating protein 2
30	224764_at	ARHGAP21	13.530426	-14.301517	1.3353342	4.23E-10	Rho GTPase activating protein 21
31	201230_s_at	ARIH2	10.754214	-11.745013	1.1312537	6.33E-09	ariadne RBR E3 ubiquitin protein ligase 2
32	202206_at	ARL4C	20.424018	-23.22285	4.4963926	4.21E-13	ADP ribosylation factor like GTPase 4C
33	202207_at	ARL4C	18.865281	-20.791112	5.8262263	2.09E-12	ADP ribosylation factor like GTPase 4C
34	202208_s_at	ARL4C	12.521636	-13.319721	2.4879536	1.13E-09	ADP ribosylation factor like GTPase 4C
35	223696_at	ARSD	10.30455	11.371213	-2.325981	9.79E-09	arylsulfatase D
36	224791_at	ASAP1	18.384832	-20.098802	2.0686903	3.40E-12	ArfGAP with SH3 domain, ankyrin repeat and PH domain 1
37	224790_at	ASAP1	16.9528	-18.176482	1.8610886	1.43E-11	ArfGAP with SH3 domain, ankyrin repeat and PH domain 1
38	221039_s_at	ASAP1	16.817511	-18.005056	2.0375673	1.64E-11	ArfGAP with SH3 domain, ankyrin repeat and PH domain 1
39	224796_at	ASAP1	16.606185	-17.740594	1.7908866	2.03E-11	ArfGAP with SH3 domain, ankyrin repeat and PH domain 1
40	236533_at	ASAP1	12.774759	-13.560056	1.1918423	8.86E-10	ArfGAP with SH3 domain, ankyrin repeat and PH domain 1
41	206043_s_at	ATP2C2	16.636482	17.778265	-2.6814482	1.97E-11	ATPase secretory pathway Ca2+ transporting 2
42	208764_s_at	ATP5G2	12.809907	13.59374	-1.4835335	8.57E-10	ATP synthase, H+ transporting, mitochondrial Fo complex subunit C2
43	213106_at	ATP8A1	22.628834	27.235009	-3.1253898	4.14E-14	ATPase phospholipid transporting 8A1
44	212114_at	ATXN7L3B	25.508767	33.825817	-2.0874528	1.73E-15	ataxin 7 like 3B
45	202686_s_at	AXL	18.420764	-20.149714	7.2430362	3.28E-12	AXL receptor tyrosine kinase
46	225612_s_at	B3GNT5	15.421208	-16.328807	5.1154156	6.58E-11	UDP-GlcNAc:betaGal beta-1,3-N-acetylglucosaminyltransferase 5
47	209406_at	BAG2	25.738842	-34.437008	3.8336023	1.33E-15	BCL2 associated athanogene 2
48	230750_at	BAG2	14.107968	-14.893606	1.450601	2.40E-10	BCL2 associated athanogene 2
49	225285_at	BCAT1	31.078706	-54.613934	5.7658465	1.46E-18	branched chain amino acid transaminase 1
50	226517_at	BCAT1	26.816285	-37.506988	5.5100258	3.78E-16	branched chain amino acid transaminase 1
51	214452_at	BCAT1	24.474937	-31.248616	4.1102373	5.53E-15	branched chain amino acid transaminase 1
52	214390_s_at	BCAT1	18.449979	-20.191208	2.2245001	3.18E-12	branched chain amino acid transaminase 1
53	203795_s_at	BCL7A	11.93382	12.776419	-1.2628278	2.01E-09	BCL tumor suppressor 7A
54	239367_at	BDNF	24.876821	-32.219079	2.8308213	3.53E-15	brain derived neurotrophic factor
55	206382_s_at	BDNF	21.584369	-25.241352	4.7480061	1.25E-13	brain derived neurotrophic factor
56	213429_at	BICC1	13.497093	-14.26803	5.2698264	4.37E-10	BicC family RNA binding protein 1
57	231964_at	BICD1	10.690896	-11.691753	1.5243192	6.73E-09	BICD cargo adaptor 1
58	213154_s_at	BICD2	11.632967	-12.506095	1.6644641	2.70E-09	BICD cargo adaptor 2
59	228320_x_at	BICDL1	17.732785	19.198319	-1.7355332	6.56E-12	BICD family like cargo adaptor 1
60	235095_at	BICDL2	11.749736	12.610406	-1.6292178	2.41E-09	BICD family like cargo adaptor 2
61	202931_x_at	BIN1	17.982543	-19.538083	3.2218109	5.10E-12	bridging integrator 1
62	210201_x_at	BIN1	15.666646	-16.611673	3.1704115	5.16E-11	bridging integrator 1
63	214439_x_at	BIN1	15.236272	-16.118822	3.328001	7.90E-11	bridging integrator 1
64	210202_s_at	BIN1	11.641355	-12.513562	2.0569617	2.67E-09	bridging integrator 1
65	207655_s_at	BLNK	10.363977	11.420028	-4.8057899	9.24E-09	B-cell linker
66	202701_at	BMP1	10.39053	-11.441897	0.9654802	9.01E-09	bone morphogenetic protein 1
67	218792_s_at	BSPRY	26.064655	35.328077	-3.0439382	9.12E-16	B-box and SPRY domain containing
68	222746_s_at	BSPRY	19.796933	22.208613	-3.6349383	8.05E-13	B-box and SPRY domain containing
69	205548_s_at	BTG3	11.296738	-12.209968	4.5683173	3.74E-09	BTG anti-proliferation factor 3
70	225863_s_at	C19orf12	10.736503	-11.730095	1.2151227	6.44E-09	chromosome 19 open reading frame 12
71	226107_at	C1GALT1	13.048511	-13.824447	3.0500306	6.78E-10	core 1 synthase, glycoprotein-N-acetylgalactosamine 3-beta-galactos
72	226105_at	C1GALT1	13.041183	-13.817308	3.0486652	6.83E-10	core 1 synthase, glycoprotein-N-acetylgalactosamine 3-beta-galactos
73	1554246_at	C1orf210	12.738661	13.525541	-2.2201435	9.18E-10	chromosome 1 open reading frame 210
74	223459_s_at	C1orf56	12.377961	13.185036	-1.5608063	1.31E-09	chromosome 1 open reading frame 56
75	1552575_a_at	C6orf141	16.078374	17.097202	-4.2776317	3.43E-11	chromosome 6 open reading frame 141
76	229964_at	C9orf152	15.794368	16.760795	-4.6862191	4.55E-11	chromosome 9 open reading frame 152
77	1552755_at	C9orf66	12.507609	13.306517	-1.0594082	1.15E-09	chromosome 9 open reading frame 66
78	210108_at	CACNA1D	19.136739	21.193631	-3.0364097	1.58E-12	calcium voltage-gated channel subunit alpha1 D
79	1555993_at	CACNA1D	14.169259	14.957788	-1.6909512	2.26E-10	calcium voltage-gated channel subunit alpha1 D
80	228555_at	CAMK2D	12.708931	-13.497176	3.3844655	9.45E-10	calcium/calmodulin dependent protein kinase II delta
81	224994_at	CAMK2D	10.741922	-11.734657	4.1223684	6.41E-09	calcium/calmodulin dependent protein kinase II delta
82	1568617_a_at	CAMSAP3	17.046598	18.29632	-2.4319489	1.31E-11	calmodulin regulated spectrin associated protein family member 3
83	213798_s_at	CAP1	12.14873	-12.972697	1.8574658	1.63E-09	adenylate cyclase associated protein 1
84	200625_s_at	CAP1	10.788489	-11.773929	1.8371226	6.12E-09	adenylate cyclase associated protein 1
85	210026_s_at	CARD10	18.808502	-20.707974	3.0963261	2.21E-12	caspase recruitment domain family member 10

86	203065_s_at	CAV1	21.286671	-24.704867	6.5879461	1.72E-13	caveolin 1
87	212097_at	CAV1	17.83691	-19.339204	8.0735134	5.91E-12	caveolin 1
88	203323_at	CAV2	22.961448	-27.909038	6.3897616	2.90E-14	caveolin 2
89	203324_s_at	CAV2	18.68998	-20.535581	6.9012164	2.49E-12	caveolin 2
90	208056_s_at	CBFA2T3	19.82568	22.254012	-3.2068468	7.81E-13	CBFA2/RUNX1 translocation partner 3
91	220581_at	CCDC170	20.444468	23.256791	-3.8632652	4.13E-13	coiled-coil domain containing 170
92	226713_at	CCDC50	10.581732	-11.600416	1.9073104	7.48E-09	coiled-coil domain containing 50
93	223300_s_at	CCDC82	10.585087	-11.603214	2.4767784	7.46E-09	coiled-coil domain containing 82
94	225045_at	CCDC88A	27.581466	-39.922706	4.1844644	1.51E-16	coiled-coil domain containing 88A
95	221078_s_at	CCDC88A	15.97534	-16.974382	2.1997987	3.80E-11	coiled-coil domain containing 88A
96	231991_at	CCM2L	11.045079	11.99235	-0.8612029	4.77E-09	CCM2 like scaffolding protein
97	208796_s_at	CCNG1	11.130852	12.06614	-1.0375211	4.39E-09	cyclin G1
98	212014_x_at	CD44	17.040224	-18.28815	3.2100945	1.31E-11	CD44 molecule (Indian blood group)
99	209835_x_at	CD44	16.795687	-17.977559	3.2816216	1.68E-11	CD44 molecule (Indian blood group)
100	204490_s_at	CD44	16.462761	-17.563371	3.2409946	2.34E-11	CD44 molecule (Indian blood group)
101	210916_s_at	CD44	13.590928	-14.362485	3.6411554	3.99E-10	CD44 molecule (Indian blood group)
102	1557905_s_at	CD44	10.886762	-11.857175	3.6846567	5.57E-09	CD44 molecule (Indian blood group)
103	204489_s_at	CD44	10.678198	-11.681098	2.7948278	6.81E-09	CD44 molecule (Indian blood group)
104	225685_at	CDC42EP3	11.642534	-12.514612	5.7799705	2.67E-09	CDC42 effector protein 3
105	201130_s_at	CDH1	29.41319	46.718388	-5.7686483	1.48E-17	cadherin 1
106	201131_s_at	CDH1	24.629795	31.618053	-7.6506876	4.66E-15	cadherin 1
107	207173_x_at	CDH11	10.725542	-11.72087	6.1121225	6.51E-09	cadherin 11
108	224848_at	CDK6	21.931781	-25.884708	4.1141521	8.70E-14	cyclin dependent kinase 6
109	224847_at	CDK6	19.762378	-22.154179	4.6370049	8.34E-13	cyclin dependent kinase 6
110	224851_at	CDK6	17.421054	-18.782936	5.0851133	8.98E-12	cyclin dependent kinase 6
111	235287_at	CDK6	12.796223	-13.580617	2.4799923	8.68E-10	cyclin dependent kinase 6
112	226185_at	CDS1	24.652137	31.671814	-4.7111715	4.54E-15	CDP-diacylglycerol synthase 1
113	205709_s_at	CDS1	24.162985	30.520973	-4.5897217	7.82E-15	CDP-diacylglycerol synthase 1
114	226187_at	CDS1	18.611695	20.422563	-2.3705753	2.70E-12	CDP-diacylglycerol synthase 1
115	41660_at	CELSR1	14.202872	14.993099	-2.8017601	2.19E-10	cadherin EGF LAG seven-pass G-type receptor 1
116	228007_at	CEP85L	13.569197	-14.340558	1.4836103	4.08E-10	centrosomal protein 85 like
117	223232_s_at	CGN	12.253313	13.069187	-2.6178626	1.47E-09	cingulin
118	228560_at	CHDH	15.564884	16.493808	-2.5924177	5.71E-11	choline dehydrogenase
119	209834_at	CHST3	12.869341	-13.650873	2.2519573	8.08E-10	carbohydrate sulfotransferase 3
120	225623_at	CIPC	13.145805	13.919554	-1.8139651	6.17E-10	CLOCK interacting pacemaker
121	200811_at	CIRBP	11.906806	12.751936	-1.7679389	2.07E-09	cold inducible RNA binding protein
122	200810_s_at	CIRBP	10.727624	11.722622	-1.786412	6.50E-09	cold inducible RNA binding protein
123	202712_s_at	CKMT1A///CKMT1E	18.654178	20.483811	-5.0127582	2.59E-12	creatine kinase, mitochondrial 1A///creatine kinase, mitochondrial 1B
124	203954_x_at	CLDN3	20.857277	23.954085	-3.5988961	2.69E-13	claudin 3
125	203953_s_at	CLDN3	18.12669	19.737063	-5.8177835	4.41E-12	claudin 3
126	224733_at	CMTM3	20.339355	-23.082931	2.9834898	4.60E-13	CKLF like MARVEL transmembrane domain containing 3
127	1552344_s_at	CNOT7	11.269488	-12.186239	1.5251406	3.84E-09	CCR4-NOT transcription complex subunit 7
128	1553510_s_at	CNOT9	11.278038	-12.19368	0.907366	3.81E-09	CCR4-NOT transcription complex subunit 9
129	211964_at	COL4A2	16.591989	-17.722972	6.9736434	2.06E-11	collagen type IV alpha 2 chain
130	211966_at	COL4A2	13.891913	-14.669444	5.3048985	2.97E-10	collagen type IV alpha 2 chain
131	204724_s_at	COL9A3	18.138173	19.753006	-2.2265027	4.36E-12	collagen type IX alpha 3 chain
132	222644_s_at	COLGALT1	11.891138	-12.737755	2.525478	2.10E-09	collagen beta(1-O)galactosyltransferase 1
133	218473_s_at	COLGALT1	10.911328	-11.878064	2.0786923	5.44E-09	collagen beta(1-O)galactosyltransferase 1
134	223114_at	COQ5	11.894566	12.740857	-1.8708364	2.09E-09	coenzyme Q5, methyltransferase
135	222409_at	CORO1C	11.265377	-12.182663	2.0532098	3.85E-09	coronin 1C
136	221059_s_at	COTL1	10.435584	-11.479084	3.0824534	8.62E-09	coactosin like F-actin binding protein 1
137	225129_at	CPNE2	10.567161	-11.588271	2.0183392	7.59E-09	copine 2
138	203633_at	CPT1A	11.460981	12.353844	-1.7678662	3.19E-09	carnitine palmitoyltransferase 1A
139	202575_at	CRABP2	11.194085	12.12079	-4.0281913	4.13E-09	cellular retinoic acid binding protein 2
140	227429_at	CRACR2B	16.067612	17.084332	-1.8411259	3.47E-11	calcium release activated channel regulator 2B
141	226455_at	CREB3L4	28.074537	41.599231	-4.6781436	8.20E-17	cAMP responsive element binding protein 3 like 4
142	208774_at	CSNK1D	13.495827	14.26676	-0.9673608	4.38E-10	casein kinase 1 delta
143	214297_at	CSPG4	12.592175	-13.386303	1.191357	1.06E-09	chondroitin sulfate proteoglycan 4
144	209101_at	CTGF	18.14063	-19.756419	7.4492369	4.35E-12	connective tissue growth factor
145	225647_s_at	CTSC	14.357297	-15.156352	3.9769727	1.88E-10	cathepsin C
146	225646_at	CTSC	10.624576	-11.636191	3.9392488	7.18E-09	cathepsin C
147	203666_at	CXCL12	14.229573	15.021205	-4.0601656	2.13E-10	C-X-C motif chemokine ligand 12
148	209164_s_at	CYB561	14.074115	14.858268	-2.0829135	2.48E-10	cytochrome b561
149	209163_at	CYB561	11.465731	12.358026	-2.4051558	3.17E-09	cytochrome b561
150	210764_s_at	CYR61	21.625751	-25.316994	4.4969411	1.20E-13	cysteine rich angiogenic inducer 61
151	201289_at	CYR61	20.017372	-22.559391	3.8490511	6.41E-13	cysteine rich angiogenic inducer 61
152	216389_s_at	DCAF11	12.208525	13.027786	-0.8593834	1.54E-09	DDB1 and CUL4 associated factor 11
153	201886_at	DCAF11	11.015532	11.967022	-0.8217861	4.91E-09	DDB1 and CUL4 associated factor 11
154	222925_at	DCDC2	16.632851	17.773745	-2.424343	1.98E-11	doublecortin domain containing 2
155	212919_at	DCP2	12.3205	13.131516	-1.7353649	1.38E-09	decapping mRNA 2
156	217973_at	DCXR	22.274471	26.538338	-2.3628888	6.04E-14	dicarbonyl and L-xylulose reductase
157	243711_at	DDAH1	11.83478	-12.686862	1.886993	2.22E-09	dimethylarginine dimethylaminohydrolase 1
158	236496_at	DEGS2	22.992366	27.972713	-3.4480173	2.80E-14	delta 4-desaturase, sphingolipid 2
159	53991_at	DENND2A	11.448406	-12.342776	2.6504667	3.23E-09	DENN domain containing 2A
160	221081_s_at	DENND2D	10.926555	11.891027	-3.1137107	5.36E-09	DENN domain containing 2D
161	203695_s_at	DFNA5	22.395597	-26.774044	4.6556258	5.31E-14	DFNA5, deafness associated tumor suppressor
162	205603_s_at	DIAPH2	16.290914	-17.353392	3.5231427	2.78E-11	diaphanous related formin 2
163	205726_at	DIAPH2	10.384153	-11.436642	3.3412617	9.06E-09	diaphanous related formin 2
164	225061_at	DNAJA4	11.265646	12.182897	-3.8336837	3.85E-09	DnaJ heat shock protein family (Hsp40) member A4
165	1554334_a_at	DNAJA4	11.105751	12.044505	-3.1516083	4.50E-09	DnaJ heat shock protein family (Hsp40) member A4
166	240633_at	DOK7	15.317074	16.210239	-2.6342488	7.30E-11	docking protein 7
167	204646_at	DPYD	15.562632	-16.491208	6.1932929	5.72E-11	dihydropyrimidine dehydrogenase
168	200762_at	DPYSL2	14.650104	-15.470604	4.6827553	1.41E-10	dihydropyrimidinase like 2
169	204751_x_at	DSC2	13.326654	14.097953	-2.8239099	5.17E-10	desmocollin 2
170	1562821_a_at	DSCAM-AS1	36.083034	105.178467	-9.4892957	8.74E-23	DSCAM antisense RNA 1
171	218854_at	DSE	13.897359	-14.675054	4.6453686	2.95E-10	dermatan sulfate epimerase
172	238467_at	DYNLL2	14.806393	15.640908	-2.7670549	1.21E-10	dynein light chain LC8-type 2

173	238116_at	DYNLRB2	17.347748	18.686626	-3.0100684	9.66E-12	dynein light chain roadblock-type 2
174	202623_at	EAPP	19.514346	21.76776	-1.3935298	1.08E-12	E2F associated phosphoprotein
175	225993_at	EARS2	11.666097	12.535612	-0.9538488	2.61E-09	glutamyl-tRNA synthetase 2, mitochondrial
176	225275_at	EDIL3	24.352066	-30.959407	8.4201672	6.34E-15	EGF like repeats and discoidin domains 3
177	207379_at	EDIL3	11.691295	-12.558104	3.7819109	2.55E-09	EGF like repeats and discoidin domains 3
178	204540_at	EEF1A2	12.163213	12.986021	-3.6996443	1.61E-09	eukaryotic translation elongation factor 1 alpha 2
179	224999_at	EGFR	11.769322	-12.627978	4.033678	2.36E-09	epidermal growth factor receptor
180	211937_at	EIF4B	11.775693	12.633698	-2.0744612	2.35E-09	eukaryotic translation initiation factor 4B
181	221773_at	ELK3	10.704964	-11.703569	3.3540828	6.64E-09	ELK3, ETS transcription factor
182	226099_at	ELL2	12.769406	-13.554932	3.379616	8.91E-10	elongation factor for RNA polymerase II 2
183	219411_at	ELMO3	18.803183	20.700202	-2.8557733	2.22E-12	engulfment and cell motility 3
184	201324_at	EMP1	18.956051	-20.924775	6.1540808	1.90E-12	epithelial membrane protein 1
185	201325_s_at	EMP1	13.370004	-14.14103	3.9879357	4.95E-10	epithelial membrane protein 1
186	225078_at	EMP2	17.112782	18.381367	-3.819828	1.22E-11	epithelial membrane protein 2
187	225079_at	EMP2	12.157275	12.980557	-3.3388533	1.62E-09	epithelial membrane protein 2
188	203729_at	EMP3	19.302064	-21.442931	5.5101551	1.34E-12	epithelial membrane protein 3
189	201231_s_at	ENO1	10.999782	-11.953539	1.8887219	4.99E-09	enolase 1
190	201719_s_at	EPB41L2	10.300766	-11.36811	3.0099591	9.82E-09	erythrocyte membrane protein band 4.1 like 2
191	229292_at	EPB41L5	13.22561	13.998017	-4.090548	5.71E-10	erythrocyte membrane protein band 4.1 like 5
192	209368_at	EPHX2	12.017573	12.852592	-2.367665	1.85E-09	epoxide hydrolase 2
193	220318_at	EPN3	15.588461	16.521042	-1.8926381	5.58E-11	epsin 3
194	223895_s_at	EPN3	12.695299	13.484188	-3.0764159	9.58E-10	epsin 3
195	232164_s_at	EPPK1	22.936505	27.857797	-5.0239339	2.98E-14	epiplakin 1
196	232165_at	EPPK1	22.627904	27.233154	-4.4261927	4.15E-14	epiplakin 1
197	216836_s_at	ERBB2	15.681487	16.628933	-1.6559781	5.09E-11	erb-b2 receptor tyrosine kinase 2
198	202454_s_at	ERBB3	23.415445	28.862144	-5.9579922	1.77E-14	erb-b2 receptor tyrosine kinase 3
199	226213_at	ERBB3	18.45256	20.194879	-6.0122124	3.17E-12	erb-b2 receptor tyrosine kinase 3
200	1563253_s_at	ERBB3	11.73548	12.59763	-1.6857199	2.44E-09	erb-b2 receptor tyrosine kinase 3
201	214053_at	ERBB4	28.90698	44.675428	-4.9634768	2.86E-17	erb-b2 receptor tyrosine kinase 4
202	206794_at	ERBB4	12.752264	13.538538	-1.4695374	9.06E-10	erb-b2 receptor tyrosine kinase 4
203	205225_at	ESR1	30.988244	54.129848	-7.4798547	1.67E-18	estrogen receptor 1
204	225846_at	ESRP1	35.091865	88.61056	-7.0315518	1.12E-21	epithelial splicing regulatory protein 1
205	219121_s_at	ESRP1	24.009498	30.170832	-5.4778632	9.26E-15	epithelial splicing regulatory protein 1
206	201931_at	ETFA	11.127752	12.063465	-1.5892204	4.41E-09	electron transfer flavoprotein alpha subunit
207	224833_at	ETS1	31.643838	-57.814483	6.7613471	6.30E-19	ETS proto-oncogene 1, transcription factor
208	1555355_a_at	ETS1	17.183779	-18.473055	3.9507986	1.14E-11	ETS proto-oncogene 1, transcription factor
209	221911_at	ETV1	11.343483	-12.250766	4.080814	3.57E-09	ETS variant 1
210	203349_s_at	ETV5	17.855128	-19.363966	4.2494585	5.80E-12	ETS variant 5
211	230102_at	ETV5	16.257611	-17.312995	4.1692305	2.87E-11	ETS variant 5
212	203348_s_at	ETV5	15.283791	-16.172522	3.1537212	7.54E-11	ETS variant 5
213	225764_at	ETV6	21.426035	-24.95435	1.8043359	1.48E-13	ETS variant 6
214	235056_at	ETV6	17.383664	-18.733748	2.5669673	9.32E-12	ETS variant 6
215	239364_at	ETV6	14.283226	-15.077835	1.1006902	2.02E-10	ETS variant 6
216	218363_at	EXD2	17.361647	18.704847	-1.6034719	9.53E-12	exonuclease 3'-5' domain containing 2
217	230183_at	EXT1	24.046115	-30.253902	3.6072121	8.89E-15	exostosin glycosyltransferase 1
218	214985_at	EXT1	15.880202	-16.861761	2.6929795	4.18E-11	exostosin glycosyltransferase 1
219	230792_at	FAAH2	11.467182	12.359304	-1.0202304	3.17E-09	fatty acid amide hydrolase 2
220	226863_at	FAM110C	12.523909	13.321862	-3.0689197	1.13E-09	family with sequence similarity 110 member C
221	225395_s_at	FAM120AOS	13.79687	14.571851	-1.7130811	3.26E-10	family with sequence similarity 120A opposite strand
222	212771_at	FAM171A1	15.04064	-15.899584	4.1183219	9.59E-11	family with sequence similarity 171 member A1
223	51158_at	FAM174B	15.425045	16.333192	-2.5188928	6.56E-11	family with sequence similarity 174 member B
224	221880_s_at	FAM174B	12.816696	13.600255	-2.1241596	8.51E-10	family with sequence similarity 174 member B
225	1557014_a_at	FAM201A	13.756924	14.531018	-2.1779456	3.39E-10	family with sequence similarity 201 member A
226	225327_at	FAM214A	19.66798	22.006227	-3.9248275	9.19E-13	family with sequence similarity 214 member A
227	227811_at	FGD3	16.273169	17.331855	-1.7174962	2.83E-11	FYVE, RhoGEF and PH domain containing 3
228	227948_at	FGD4	11.22264	12.145539	-2.4337312	4.02E-09	FYVE, RhoGEF and PH domain containing 4
229	226705_at	FGFR1	13.202903	-13.975651	2.1082059	5.83E-10	fibroblast growth factor receptor 1
230	200895_s_at	FKBP4	12.028304	12.862382	-1.7779711	1.84E-09	FK506 binding protein 4
231	200859_x_at	FLNA	10.909702	-11.87668	2.7311361	5.44E-09	filamin A
232	213746_s_at	FLNA	10.583808	-11.602148	2.9987041	7.47E-09	filamin A
233	214752_x_at	FLNA	10.495559	-11.528745	2.810784	8.13E-09	filamin A
234	226184_at	FMNL2	11.332299	-12.240994	4.3994401	3.61E-09	formin like 2
235	204420_at	FOSL1	16.326732	-17.396947	4.238034	2.68E-11	FOS like 1, AP-1 transcription factor subunit
236	225262_at	FOSL2	14.699519	-15.524255	2.274496	1.34E-10	FOS like 2, AP-1 transcription factor subunit
237	218881_s_at	FOSL2	13.849216	-14.625525	1.5974342	3.10E-10	FOS like 2, AP-1 transcription factor subunit
238	218880_at	FOSL2	11.324708	-12.234366	2.7043648	3.64E-09	FOS like 2, AP-1 transcription factor subunit
239	204667_at	FOXA1	23.79936	29.699606	-5.9499635	1.17E-14	forkhead box A1
240	237086_at	FOXA1	21.619697	25.305911	-6.0268709	1.21E-13	forkhead box A1
241	219889_at	FRAT1	21.228685	24.601916	-2.8254625	1.82E-13	frequently rearranged in advanced T-cell lymphomas 1
242	209864_at	FRAT2	14.864842	15.705066	-2.3463377	1.14E-10	frequently rearranged in advanced T-cell lymphomas 2
243	226045_at	FRS2	11.535033	12.419197	-1.1050327	2.97E-09	fibroblast growth factor receptor substrate 2
244	226847_at	FST	26.333856	-36.08801	5.1104072	6.67E-16	follistatin
245	204948_s_at	FST	21.030073	-24.253017	2.5572539	2.24E-13	follistatin
246	212788_x_at	FTL	12.21208	-13.031069	1.3860756	1.53E-09	ferritin light chain
247	202489_s_at	FXYD3	24.238874	30.695989	-3.5990263	7.19E-15	FXYD domain containing ion transport regulator 3
248	205219_s_at	GALK2	12.631652	13.423697	-1.3836109	1.02E-09	galactokinase 2
249	204921_at	GAS8	10.592537	11.609429	-0.8452562	7.40E-09	growth arrest specific 8
250	209603_at	GATA3	22.025469	26.061493	-5.9391549	7.87E-14	GATA binding protein 3
251	210002_at	GATA6	13.595019	-14.366616	3.9900622	3.97E-10	GATA binding protein 6
252	223434_at	GBP3	15.679544	-16.626672	5.9024071	5.10E-11	guanylate binding protein 3
253	234986_at	GCLM	19.062105	-21.082127	2.6855658	1.71E-12	glutamate-cysteine ligase modifier subunit
254	203925_at	GCLM	17.153721	-18.434179	2.1796376	1.17E-11	glutamate-cysteine ligase modifier subunit
255	215380_s_at	GGCT	13.737181	14.510875	-1.2575306	3.46E-10	gamma-glutamylcyclotransferase
256	225604_s_at	GLIPR2	21.804314	-25.646449	1.96229	9.95E-14	GLI pathogenesis related 2
257	225602_at	GLIPR2	13.239898	-14.012109	1.3144352	5.63E-10	GLI pathogenesis related 2
258	218241_at	GOLGA5	14.707135	15.532539	-1.2010507	1.33E-10	golgin A5
259	229372_at	GOLT1A	13.172922	13.946169	-2.149709	6.01E-10	golgi transport 1A

260	212510_at	GPD1L	11.807268	12.662083	-2.6204133	2.28E-09	glycerol-3-phosphate dehydrogenase 1-like
261	223423_at	GPR160	15.436826	16.346663	-4.9301348	6.48E-11	G protein-coupled receptor 160
262	214104_at	GPR161	10.609588	-11.623666	1.899168	7.28E-09	G protein-coupled receptor 161
263	227769_at	GPR27	20.132244	22.744629	-4.38935	5.70E-13	G protein-coupled receptor 27
264	225463_x_at	GPR89A///GPR89B	12.19451	13.014856	-1.2241209	1.56E-09	G protein-coupled receptor 89A///G protein-coupled receptor 89B
265	222140_s_at	GPR89A///GPR89B	10.686242	11.687847	-1.2771739	6.76E-09	G protein-coupled receptor 89A///G protein-coupled receptor 89B
266	220642_x_at	GPR89A///GPR89B	10.384085	11.436586	-1.186384	9.06E-09	G protein-coupled receptor 89A///G protein-coupled receptor 89B
267	200736_s_at	GPX1	26.390008	-36.249327	7.3205306	6.24E-16	glutathione peroxidase 1
268	205862_at	GREB1	31.830641	58.94527	-7.4059686	4.73E-19	growth regulation by estrogen in breast cancer 1
269	222830_at	GRHL1	25.823444	34.665456	-4.2887244	1.21E-15	grainyhead like transcription factor 1
270	1552685_a_at	GRHL1	18.475674	20.22778	-1.9155796	3.10E-12	grainyhead like transcription factor 1
271	219388_at	GRHL2	31.147097	54.98483	-4.7360054	1.33E-18	grainyhead like transcription factor 2
272	212056_at	GSE1	18.262813	19.926942	-3.6501937	3.84E-12	Gse1 coiled-coil protein
273	212057_at	GSE1	13.67208	14.444646	-3.7611205	3.68E-10	Gse1 coiled-coil protein
274	227163_at	GSTO2	22.648173	27.273658	-3.9766775	4.06E-14	glutathione S-transferase omega 2
275	200824_at	GSTP1	23.248932	-28.508008	4.3439872	2.12E-14	glutathione S-transferase pi 1
276	213357_at	GTF2H5	10.868731	-11.841863	0.8018362	5.66E-09	general transcription factor IIH subunit 5
277	214500_at	H2AFY	15.472209	-16.387189	1.4552035	6.26E-11	H2A histone family member Y
278	218445_at	H2AFY2	24.471759	31.241093	-1.8122536	5.55E-15	H2A histone family member Y2
279	227008_at	HDDC3	14.955941	15.805575	-1.5933042	1.04E-10	HD domain containing 3
280	244593_at	HID1	13.238212	14.010445	-1.4430592	5.64E-10	HID1 domain containing
281	218507_at	HILPDA	17.702756	19.15789	-2.2074304	6.76E-12	hypoxia inducible lipid droplet associated
282	1554452_a_at	HILPDA	14.899937	15.743712	-2.262191	1.10E-10	hypoxia inducible lipid droplet associated
283	214469_at	HIST1H2AE	13.204475	13.977198	-2.9745139	5.82E-10	histone cluster 1, H2ae
284	236278_at	HIST1H3E	17.182591	18.471517	-3.0756637	1.14E-11	histone cluster 1, H3e
285	214616_at	HIST1H3E	11.627493	12.501223	-1.2506463	2.71E-09	histone cluster 1, H3e
286	206110_at	HIST1H3F///HIST1H	11.259297	12.177376	-2.8773403	3.88E-09	histone cluster 1, H3f///histone cluster 1, H3b///histone cluster 1, H3
287	208180_s_at	HIST1H4H	16.067972	17.084762	-1.9373812	3.47E-11	histone cluster 1, H4h
288	202708_s_at	HIST2H2BE	11.684414	12.551959	-3.6105719	2.57E-09	histone cluster 2, H2be
289	225792_at	HOOK1	29.834504	48.532356	-4.7608213	8.41E-18	hook microtubule tethering protein 1
290	203285_s_at	HS2ST1	10.697654	-11.697429	0.9470543	6.69E-09	heparan sulfate 2-O-sulfotransferase 1
291	200942_s_at	HSBP1	13.212236	13.98484	-0.9861937	5.78E-10	heat shock factor binding protein 1
292	223248_at	HSDL1	15.63965	16.580324	-1.4635998	5.30E-11	hydroxysteroid dehydrogenase like 1
293	207949_s_at	ICA1	27.699408	40.314676	-3.4250049	1.30E-16	islet cell autoantigen 1
294	210547_x_at	ICA1	22.362205	26.708816	-3.9323294	5.51E-14	islet cell autoantigen 1
295	206332_s_at	IFI16	13.072613	-13.847951	6.3106596	6.63E-10	interferon gamma inducible protein 16
296	208966_x_at	IFI16	10.979163	-11.935908	5.7280749	5.09E-09	interferon gamma inducible protein 16
297	225330_at	IGF1R	13.503302	14.274263	-2.4794672	4.35E-10	insulin like growth factor 1 receptor
298	218847_at	IGF2BP2	15.213119	-16.092722	3.732705	8.09E-11	insulin like growth factor 2 mRNA binding protein 2
299	202718_at	IGFBP2	16.834737	18.026791	-3.9586144	1.61E-11	insulin like growth factor binding protein 2
300	201163_s_at	IGFBP7	21.226	-24.597161	8.6118463	1.83E-13	insulin like growth factor binding protein 7
301	201162_at	IGFBP7	11.076157	-12.019041	5.0848926	4.63E-09	insulin like growth factor binding protein 7
302	219255_x_at	IL17RB	21.371976	24.857231	-2.1706107	1.57E-13	interleukin 17 receptor B
303	224156_x_at	IL17RB	16.87818	18.081724	-2.3385482	1.55E-11	interleukin 17 receptor B
304	224361_s_at	IL17RB	15.173202	16.04782	-1.8878241	8.41E-11	interleukin 17 receptor B
305	243541_at	IL31RA	12.869918	-13.651429	2.1723202	8.08E-10	interleukin 31 receptor A
306	205207_at	IL6	18.063119	-19.649046	4.0830339	4.70E-12	interleukin 6
307	235583_at	ILDR1	16.282743	17.343472	-2.3923663	2.80E-11	immunoglobulin like domain containing receptor 1
308	204030_s_at	IQCJ-SCHIP1///SCH	19.269178	-21.393086	3.6451413	1.38E-12	IQCJ-SCHIP1 readthrough///schwannomin interacting protein 1
309	1552477_a_at	IRF6	14.4555	15.261055	-3.5563517	1.71E-10	interferon regulatory factor 6
310	228462_at	IRX2	10.881645	11.852828	-3.4917452	5.59E-09	iroquois homeobox 2
311	1553678_a_at	ITGB1	18.100906	-19.701313	2.6613083	4.53E-12	integrin subunit beta 1
312	211945_s_at	ITGB1	17.524172	-18.919291	1.9346283	8.09E-12	integrin subunit beta 1
313	1553530_a_at	ITGB1	16.751293	-17.921757	2.6859748	1.76E-11	integrin subunit beta 1
314	225582_at	ITPRIP	16.10534	-17.129493	2.4074639	3.34E-11	inositol 1,4,5-trisphosphate receptor interacting protein
315	227677_at	JAK3	12.356574	13.165093	-1.5321551	1.33E-09	Janus kinase 3
316	229139_at	JPH1	15.048139	15.907934	-3.8951316	9.52E-11	junctophilin 1
317	200048_s_at	JTB	12.692959	13.481959	-0.712543	9.60E-10	jumping translocation breakpoint
318	210434_x_at	JTB	11.900835	12.74653	-0.6709257	2.08E-09	jumping translocation breakpoint
319	210927_x_at	JTB	11.661823	12.531801	-0.7039762	2.62E-09	jumping translocation breakpoint
320	218418_s_at	KANK2	15.243328	-16.126785	1.898375	7.85E-11	KN motif and ankyrin repeat domains 2
321	227713_at	KATNAL1	10.508586	-11.539555	1.9106614	8.03E-09	katanin catalytic subunit A1 like 1
322	223658_at	KCNK6	13.486008	14.256911	-2.46269	4.42E-10	potassium two pore domain channel subfamily K member 6
323	212192_at	KCTD12	19.561307	-21.840348	5.6567226	1.02E-12	potassium channel tetramerization domain containing 12
324	212188_at	KCTD12	16.265526	-17.322587	3.5799425	2.85E-11	potassium channel tetramerization domain containing 12
325	203143_s_at	KIAA0040	18.603322	20.410515	-2.4360677	2.72E-12	KIAA0040
326	221874_at	KIAA1324	21.284855	24.701636	-4.5540311	1.72E-13	KIAA1324
327	243349_at	KIAA1324	18.834395	20.745842	-3.4172029	2.15E-12	KIAA1324
328	226248_s_at	KIAA1324	18.128971	19.740229	-5.290602	4.40E-12	KIAA1324
329	225303_at	KIRREL	11.679223	-12.547325	4.0420187	2.58E-09	kin of IRRE like (Drosophila)
330	227261_at	KLF12	17.664553	-19.106584	3.5250844	7.03E-12	Kruppel like factor 12
331	225068_at	KLHL12	12.376296	13.183483	-1.7136656	1.31E-09	kelch like family member 12
332	220239_at	KLHL7	12.658477	-13.449161	2.0310658	9.93E-10	kelch like family member 7
333	221503_s_at	KPNA3	10.785148	-11.771107	1.4043157	6.14E-09	karyopherin subunit alpha 3
334	221502_at	KPNA3	10.571233	-11.591664	1.5061742	7.56E-09	karyopherin subunit alpha 3
335	213803_at	KPNB1	11.182289	-12.110578	1.0623066	4.18E-09	karyopherin subunit beta 1
336	203042_at	LAMP2	11.380492	-12.283151	1.4209086	3.45E-09	lysosomal associated membrane protein 2
337	201030_x_at	LDHB	22.11271	-26.227397	5.7117346	7.18E-14	lactate dehydrogenase B
338	218604_at	LEMD3	20.286256	22.995661	-1.5055296	4.86E-13	LEM domain containing 3
339	201105_at	LGALS1	10.454065	-11.494368	2.630749	8.47E-09	galectin 1
340	226413_at	LINC00938	11.177543	12.106472	-2.8558588	4.20E-09	long intergenic non-protein coding RNA 938
341	1559827_at	LINC00960	11.233582	-12.155035	3.1713725	3.98E-09	long intergenic non-protein coding RNA 960
342	239319_at	LINC00992	18.614408	20.426468	-6.7802993	2.69E-12	long intergenic non-protein coding RNA 992
343	228348_at	LINS1	11.536856	12.42081	-1.077825	2.96E-09	lines homolog 1
344	235036_at	LIX1L	17.025493	-18.269285	2.6249268	1.33E-11	limb and CNS expressed 1 like
345	225793_at	LIX1L	11.589227	-12.467218	3.0214811	2.81E-09	limb and CNS expressed 1 like
346	203713_s_at	LLGL2	11.753017	12.613348	-2.3633542	2.40E-09	LLGL2, scribble cell polarity complex component

347	230641_at	LOC100505938	16.775714	17.952431	-2.3194943	1.71E-11	uncharacterized LOC100505938
348	202237_at	LOC101928916///N	25.086258	-32.740289	7.8991912	2.79E-15	uncharacterized LOC101928916///nicotinamide N-methyltransferase
349	202238_s_at	LOC101928916///N	20.18267	-22.826481	6.1475169	5.41E-13	uncharacterized LOC101928916///nicotinamide N-methyltransferase
350	1568763_s_at	LOC102724884///L(	15.728663	16.683915	-2.1290957	4.85E-11	uncharacterized LOC102724884///programmed cell death 6 pseudog
351	1569110_x_at	LOC728613	10.717322	11.713956	-2.7892421	6.56E-09	programmed cell death 6 pseudogene
352	1568764_x_at	LOC728613///PDCD	18.98631	20.96954	-2.0326654	1.84E-12	programmed cell death 6 pseudogene///programmed cell death 6
353	225996_at	LONRF2	18.302626	19.982845	-3.9855802	3.69E-12	LON peptidase N-terminal domain and ring finger 2
354	202998_s_at	LOXL2	17.49569	-18.881526	7.6256585	8.33E-12	lysyl oxidase like 2
355	220253_s_at	LRP12	14.063796	-14.847513	4.3193623	2.51E-10	LDL receptor related protein 12
356	219631_at	LRP12	13.740961	-14.51473	4.0549297	3.44E-10	LDL receptor related protein 12
357	207358_x_at	MACF1	16.395408	-17.480767	2.5573746	2.50E-11	microtubule-actin crosslinking factor 1
358	208634_s_at	MACF1	15.075989	-15.938981	2.3602511	9.26E-11	microtubule-actin crosslinking factor 1
359	214894_x_at	MACF1	14.985608	-15.83844	2.676754	1.01E-10	microtubule-actin crosslinking factor 1
360	208633_s_at	MACF1	14.011546	-14.793166	2.4030432	2.64E-10	microtubule-actin crosslinking factor 1
361	215222_x_at	MACF1	13.641641	-14.413777	2.3335942	3.80E-10	microtubule-actin crosslinking factor 1
362	36711_at	MAFF	15.400853	-16.305563	5.3893989	6.72E-11	MAF bZIP transcription factor F
363	205193_at	MAFF	10.932177	-11.895817	2.827436	5.33E-09	MAF bZIP transcription factor F
364	224650_at	MAL2	28.739667	44.030259	-7.3922415	3.55E-17	mal, T-cell differentiation protein 2 (gene/pseudogene)
365	235457_at	MAML2	10.906601	-11.874042	3.0804246	5.46E-09	mastermind like transcriptional coactivator 2
366	242794_at	MAML3	15.442964	16.353686	-2.7318214	6.44E-11	mastermind like transcriptional coactivator 3
367	226084_at	MAP1B	16.361171	-17.438929	5.4944646	2.59E-11	microtubule associated protein 1B
368	212233_at	MAP1B	15.177304	-16.052429	5.0882384	8.38E-11	microtubule associated protein 1B
369	214577_at	MAP1B	14.313983	-15.110391	3.5640083	1.96E-10	microtubule associated protein 1B
370	212566_at	MAP4	14.583883	-15.398987	1.8082305	1.50E-10	microtubule associated protein 4
371	212567_s_at	MAP4	11.577312	-12.456646	1.3445384	2.85E-09	microtubule associated protein 4
372	243_g_at	MAP4	11.547605	-12.430322	1.5896072	2.93E-09	microtubule associated protein 4
373	202890_at	MAP7	19.659984	21.993744	-4.4854909	9.26E-13	microtubule associated protein 7
374	202889_x_at	MAP7	17.036245	18.283052	-2.3225517	1.32E-11	microtubule associated protein 7
375	215471_s_at	MAP7	15.494585	16.412869	-1.9999055	6.12E-11	microtubule associated protein 7
376	217943_s_at	MAP7D1	11.08385	-12.025655	2.0036641	4.60E-09	MAP7 domain containing 1
377	206401_s_at	MAPT	13.269263	14.04111	-1.3640819	5.47E-10	microtubule associated protein tau
378	203928_x_at	MAPT	12.698761	13.487485	-1.2410475	9.55E-10	microtubule associated protein tau
379	201669_s_at	MARCKS	33.636481	-72.392293	8.1335987	2.24E-20	myristoylated alanine rich protein kinase C substrate
380	201670_s_at	MARCKS	30.612932	-52.196677	7.0687728	2.86E-18	myristoylated alanine rich protein kinase C substrate
381	225897_at	MARCKS	27.977851	-41.262493	6.9147492	9.25E-17	myristoylated alanine rich protein kinase C substrate
382	213002_at	MARCKS	21.720863	-25.491858	4.0198479	1.09E-13	myristoylated alanine rich protein kinase C substrate
383	201668_x_at	MARCKS	21.585423	-25.243275	4.2117993	1.25E-13	myristoylated alanine rich protein kinase C substrate
384	235955_at	MARVELD2	14.049762	14.832897	-2.6558424	2.54E-10	MARVEL domain containing 2
385	233634_at	MARVELD3	10.314772	11.379597	-1.5433593	9.69E-09	MARVEL domain containing 3
386	204179_at	MB	12.370142	13.177742	-3.8089292	1.32E-09	myoglobin
387	226797_at	MBTD1	15.713022	16.665665	-1.7140712	4.93E-11	mbt domain containing 1
388	225160_x_at	MDM2	12.939471	13.718572	-1.8069746	7.55E-10	MDM2 proto-oncogene
389	212209_at	MED13L	10.472091	11.509291	-2.0634174	8.32E-09	mediator complex subunit 13 like
390	225222_at	MFSD14A	13.09675	-13.871526	1.1760893	6.47E-10	major facilitator superfamily domain containing 14A
391	212473_s_at	MICAL2	10.635519	-11.645344	5.6395775	7.10E-09	microtubule associated monooxygenase, calponin and LIM domain c
392	225381_at	MIR100HG	11.610191	-12.485838	5.3775906	2.76E-09	mir-100-let-7a-2 cluster host gene
393	214696_at	MIR22///MIR22HG	12.428708	-13.232466	2.6172074	1.24E-09	microRNA 22///MIR22 host gene
394	212594_at	MIR4680///PDCD4	11.597613	12.474663	-2.9137552	2.79E-09	microRNA 4680///programmed cell death 4 (neoplastic transformatic
395	225374_at	MIR4723///TMEM1!	11.794981	12.651031	-0.9042546	2.30E-09	microRNA 4723///transmembrane protein 199
396	219395_at	MIR6773///ESRP2	16.990135	18.224084	-3.6103205	1.38E-11	microRNA 6773///epithelial splicing regulatory protein 2
397	226066_at	MITF	14.82089	-15.656798	3.034376	1.19E-10	melanogenesis associated transcription factor
398	207233_s_at	MITF	11.744799	-12.605981	2.2590472	2.42E-09	melanogenesis associated transcription factor
399	1555820_a_at	MKS1	11.016917	11.968208	-1.2390602	4.91E-09	Meckel syndrome, type 1
400	238451_at	MPP7	14.831644	15.668595	-5.0425673	1.18E-10	membrane palmitoylated protein 7
401	238778_at	MPP7	12.803095	13.587206	-5.3712205	8.62E-10	membrane palmitoylated protein 7
402	227747_at	MPZL3	18.466885	20.215262	-2.9416083	3.13E-12	myelin protein zero like 3
403	212199_at	MRFAP1L1	12.136148	12.961131	-1.3786623	1.65E-09	Morf4 family associated protein 1 like 1
404	200600_at	MSN	20.733516	-23.742571	6.9919994	3.06E-13	moesin
405	210319_x_at	MSX2	13.384515	14.155477	-3.8498471	4.88E-10	msh homeobox 2
406	205555_s_at	MSX2	11.695396	12.561768	-3.3165071	2.54E-09	msh homeobox 2
407	213358_at	MTCL1	26.730432	-37.248886	4.243866	4.18E-16	microtubule crosslinking factor 1
408	202180_s_at	MVP	20.467831	-23.295634	2.527471	4.03E-13	major vault protein
409	204798_at	MYB	18.446317	20.186002	-5.7119466	3.19E-12	MYB proto-oncogene, transcription factor
410	215152_at	MYB	10.805755	11.788518	-1.1295243	6.02E-09	MYB proto-oncogene, transcription factor
411	218966_at	MYO5C	10.811526	11.793398	-5.6067893	5.99E-09	myosin VC
412	214156_at	MYRIP	27.765177	40.535675	-2.9825724	1.20E-16	myosin VIIA and Rab interacting protein
413	204823_at	NAV3	11.189886	-12.117154	2.8591621	4.15E-09	neuron navigator 3
414	213325_at	NECTIN3	29.538484	-47.246505	4.650027	1.25E-17	nectin cell adhesion molecule 3
415	227503_at	NECTIN3	20.396365	-23.177044	3.5960006	4.34E-13	nectin cell adhesion molecule 3
416	241310_at	NEK5	10.862867	11.836887	-2.047373	5.70E-09	NIMA related kinase 5
417	217722_s_at	NGRN	10.344587	11.404082	-1.0404578	9.42E-09	neugrin, neurite outgrowth associated
418	225911_at	NPNT	28.475612	43.040357	-5.2494323	4.96E-17	nephronectin
419	244747_at	NPNT	10.747907	11.739698	-1.0440685	6.37E-09	nephronectin
420	205440_s_at	NPY1R	12.047058	12.879506	-5.9371482	1.80E-09	neuropeptide Y receptor Y1
421	226499_at	NRARP	10.675609	11.678926	-1.9574159	6.83E-09	NOTCH-regulated ankyrin repeat protein
422	202599_s_at	NRIP1	11.888985	12.735807	-2.531276	2.10E-09	nuclear receptor interacting protein 1
423	202600_s_at	NRIP1	10.299813	11.367329	-2.7621125	9.83E-09	nuclear receptor interacting protein 1
424	212298_at	NRP1	10.722997	-11.718729	5.2206159	6.53E-09	neuropilin 1
425	203939_at	NT5E	15.202583	-16.080858	6.9884239	8.17E-11	5'-nucleotidase ecto
426	1553995_a_at	NT5E	10.330728	-11.392695	5.0239851	9.54E-09	5'-nucleotidase ecto
427	204589_at	NUAK1	13.082834	-13.85793	3.4494194	6.56E-10	NUAK family kinase 1
428	214136_at	NUDT13	10.995046	11.949487	-1.9010647	5.01E-09	nudix hydrolase 13
429	224477_s_at	NUDT16L1	13.068439	13.843878	-1.0409256	6.65E-10	nudix hydrolase 16 like 1
430	205729_at	OSMR	16.308472	-17.374729	3.7321606	2.73E-11	oncostatin M receptor
431	226621_at	OSMR	15.264995	-16.15126	3.4857854	7.68E-11	oncostatin M receptor
432	218717_s_at	P3H2	13.075348	-13.850621	4.5857893	6.61E-10	prolyl 3-hydroxylase 2
433	225622_at	PAG1	13.02339	-13.799988	2.0204844	6.95E-10	phosphoprotein membrane anchor with glycosphingolipid microdom

434	225626_at	PAG1	12.759739	-13.545685	2.7577412	9.00E-10	phosphoprotein membrane anchor with glycosphingolipid microdom
435	227354_at	PAG1	12.115391	-12.942073	1.9692558	1.69E-09	phosphoprotein membrane anchor with glycosphingolipid microdom
436	231878_at	PAGR1	15.044949	15.904382	-1.4481528	9.55E-11	PAXIP1 associated glutamate rich protein 1
437	221868_at	PAIP2B	13.613824	14.38562	-1.6132538	3.90E-10	poly(A) binding protein interacting protein 2B
438	226694_at	PALM2-AKAP2///AI	17.360411	-18.703226	6.2212897	9.54E-12	PALM2-AKAP2 readthrough///A-kinase anchoring protein 2
439	202760_s_at	PALM2-AKAP2///AI	16.370197	-17.449949	5.4447089	2.57E-11	PALM2-AKAP2 readthrough///A-kinase anchoring protein 2
440	202759_s_at	PALM2-AKAP2///AI	11.903308	-12.748769	5.069356	2.07E-09	PALM2-AKAP2 readthrough///A-kinase anchoring protein 2
441	214620_x_at	PAM	14.051336	-14.834536	1.2346141	2.54E-10	peptidylglycine alpha-amidating monooxygenase
442	202336_s_at	PAM	12.111362	-12.938376	1.494799	1.69E-09	peptidylglycine alpha-amidating monooxygenase
443	212958_x_at	PAM	10.665837	-11.670733	1.308302	6.90E-09	peptidylglycine alpha-amidating monooxygenase
444	227626_at	PAQR8	11.509133	-12.396305	2.1621505	3.04E-09	progestin and adipoQ receptor family member 8
445	223435_s_at	PCDHA1///PCDHA2	31.06765	54.554376	-5.7973278	1.49E-18	protocadherin alpha 1///protocadherin alpha 2///protocadherin alpha
446	224212_s_at	PCDHA1///PCDHA2	25.072386	32.705429	-3.4381988	2.84E-15	protocadherin alpha 1///protocadherin alpha 2///protocadherin alpha
447	210674_s_at	PCDHA1///PCDHA2	21.776629	25.595042	-4.8004132	1.02E-13	protocadherin alpha 1///protocadherin alpha 2///protocadherin alpha
448	210907_s_at	PDCD10	12.071394	12.901757	-0.8835612	1.76E-09	programmed cell death 10
449	204491_at	PDE4D	11.122656	-12.059071	2.3380143	4.43E-09	phosphodiesterase 4D
450	218273_s_at	PDP1	14.875501	-15.716794	4.1064473	1.13E-10	pyruvate dehyrogenase phosphatase catalytic subunit 1
451	222572_at	PDP1	11.583894	-12.462485	4.17752	2.83E-09	pyruvate dehyrogenase phosphatase catalytic subunit 1
452	202658_at	PEX11B	17.295533	18.618339	-1.84208	1.02E-11	peroxisomal biogenesis factor 11 beta
453	207132_x_at	PFDN5	18.248718	19.907191	-1.3907832	3.90E-12	prefoldin subunit 5
454	210908_s_at	PFDN5	17.26567	18.5794	-1.336261	1.05E-11	prefoldin subunit 5
455	228554_at	PGR	15.951202	16.945737	-7.2788333	3.89E-11	progesterone receptor
456	223470_at	PIGM	12.990229	13.767763	-2.154159	7.18E-10	phosphatidylinositol glycan anchor biosynthesis class M
457	204484_at	PIK3C2B	10.450986	11.49182	-1.992379	8.49E-09	phosphatidylinositol-4-phosphate 3-kinase catalytic subunit type 2 be
458	203879_at	PIK3CD	12.689863	-13.479011	1.9947499	9.63E-10	phosphatidylinositol-4,5-bisphosphate 3-kinase catalytic subunit delta
459	213111_at	PIKFYVE	10.527264	-11.555071	1.0287194	7.89E-09	phosphoinositide kinase, FYVE-type zinc finger containing
460	218667_at	PJA1	11.933681	-12.776293	1.4056967	2.01E-09	praja ring finger ubiquitin ligase 1
461	209873_s_at	PKP3	11.197166	12.123458	-1.3666247	4.12E-09	plakophilin 3
462	210845_s_at	PLAUR	15.952948	-16.947807	3.3841642	3.89E-11	plasminogen activator, urokinase receptor
463	211924_s_at	PLAUR	11.316659	-12.22734	2.592455	3.67E-09	plasminogen activator, urokinase receptor
464	205093_at	PLEKHA6	10.979218	11.935956	-1.7253838	5.09E-09	pleckstrin homology domain containing A6
465	222699_s_at	PLEKHF2	17.220996	18.521308	-2.5896233	1.10E-11	pleckstrin homology and FYVE domain containing 2
466	218640_s_at	PLEKHF2	16.863917	18.06367	-2.4826408	1.57E-11	pleckstrin homology and FYVE domain containing 2
467	225727_at	PLEKHH1	16.907828	18.119312	-3.1261321	1.50E-11	pleckstrin homology, MyTH4 and FERM domain containing H1
468	225726_s_at	PLEKHH1	11.884314	12.731582	-3.9797054	2.11E-09	pleckstrin homology, MyTH4 and FERM domain containing H1
469	201215_at	PLS3	16.361595	-17.439447	1.8592975	2.59E-11	plastin 3
470	242201_at	PMS2P5	10.734026	11.728009	-2.0948776	6.46E-09	PMS1 homolog 2, mismatch repair system component pseudogene 5
471	239699_s_at	PMS2P5///PMS2P1	14.337791	15.135638	-2.0039674	1.92E-10	PMS1 homolog 2, mismatch repair system component pseudogene 5
472	209598_at	PNMA2	26.057485	-35.308136	5.3491979	9.20E-16	paraneoplastic Ma antigen 2
473	218824_at	PNMAL1	19.09443	-21.130342	4.461807	1.65E-12	paraneoplastic Ma antigen family like 1
474	233772_at	POU6F2-AS2	20.958758	24.129128	-3.6620458	2.42E-13	POU6F2 antisense RNA 2
475	216347_s_at	PPP1R13B	15.539903	16.465	-1.8676687	5.85E-11	protein phosphatase 1 regulatory subunit 13B
476	224927_at	PPP1R18	13.89908	-14.676828	3.1248569	2.95E-10	protein phosphatase 1 regulatory subunit 18
477	228010_at	PPP2R2C	14.24636	15.038902	-3.5484748	2.10E-10	protein phosphatase 2 regulatory subunit Bgamma
478	224909_s_at	PREX1	25.112365	32.806027	-5.3330808	2.71E-15	phosphatidylinositol-3,4,5-trisphosphate dependent Rac exchange fac
479	224925_at	PREX1	17.111636	18.379891	-5.0154208	1.22E-11	phosphatidylinositol-3,4,5-trisphosphate dependent Rac exchange fac
480	226065_at	PRICKLE1	13.13726	-13.911177	2.8732128	6.22E-10	prickle planar cell polarity protein 1
481	226069_at	PRICKLE1	12.891577	-13.672305	3.0509377	7.91E-10	prickle planar cell polarity protein 1
482	213093_at	PRKCA	27.48818	-39.616549	4.971155	1.69E-16	protein kinase C alpha
483	213010_at	PRKCDBP	12.082941	-12.912327	4.0565445	1.74E-09	protein kinase C delta binding protein
484	206099_at	PRKCH	16.472594	17.575463	-1.7368617	2.32E-11	protein kinase C eta
485	218764_at	PRKCH	13.240009	14.012218	-3.423901	5.63E-10	protein kinase C eta
486	213847_at	PRPH	13.608716	14.380456	-1.5601655	3.92E-10	peripherin
487	226961_at	PRR15	22.441278	26.863587	-3.1282186	5.06E-14	proline rich 15
488	219127_at	PRR15L	13.525758	14.296822	-3.1223303	4.25E-10	proline rich 15 like
489	37117_at	PRR5-ARHGAP8///A	15.227883	16.10936	-3.7763521	7.97E-11	PRR5-ARHGAP8 readthrough///Rho GTPase activating protein 8
490	205980_s_at	PRR5-ARHGAP8///A	12.490063	13.290017	-3.3135102	1.17E-09	PRR5-ARHGAP8 readthrough///Rho GTPase activating protein 8
491	202525_at	PRSS8	21.770358	25.583414	-3.8444192	1.03E-13	protease, serine 8
492	216088_s_at	PSMA7	11.002427	-11.955803	1.0428174	4.98E-09	proteasome subunit alpha 7
493	204279_at	PSMB9	15.754171	-16.713719	3.4404506	4.73E-11	proteasome subunit beta 9
494	231828_at	PSMD5-AS1	13.293416	14.065006	-2.6331516	5.34E-10	PSMD5 antisense RNA 1 (head to head)
495	208790_s_at	PTRF	23.708546	-29.498809	3.8294206	1.29E-14	polymerase I and transcript release factor
496	1557938_s_at	PTRF	19.093466	-21.128903	3.7172233	1.65E-12	polymerase I and transcript release factor
497	208789_at	PTRF	18.322017	-20.010132	3.7744405	3.62E-12	polymerase I and transcript release factor
498	214443_at	PVR	19.531466	-21.794192	1.8710799	1.06E-12	poliovirus receptor
499	32699_s_at	PVR	14.421183	-15.224388	1.292476	1.77E-10	poliovirus receptor
500	216283_s_at	PVR	14.301238	-15.096892	1.9063692	1.99E-10	poliovirus receptor
501	214444_s_at	PVR	13.811536	-14.58687	1.2529348	3.21E-10	poliovirus receptor
502	212662_at	PVR	11.066915	-12.011098	1.955722	4.67E-09	poliovirus receptor
503	227332_at	PXN-AS1	11.171832	12.101532	-1.8401931	4.22E-09	PXN antisense RNA 1
504	234998_at	RAB11A	12.544447	13.341219	-1.3663991	1.11E-09	RAB11A, member RAS oncogene family
505	221810_at	RAB15	14.96783	15.818737	-1.6307431	1.03E-10	RAB15, member RAS oncogene family
506	59697_at	RAB15	12.212357	13.031324	-1.4936123	1.53E-09	RAB15, member RAS oncogene family
507	218931_at	RAB17	16.961673	18.187783	-2.7203092	1.42E-11	RAB17, member RAS oncogene family
508	218186_at	RAB25	33.999954	75.842349	-6.5666748	1.12E-20	RAB25, member RAS oncogene family
509	219562_at	RAB26	20.37768	23.146152	-2.694553	4.42E-13	RAB26, member RAS oncogene family
510	50965_at	RAB26	16.751647	17.922201	-2.0028532	1.75E-11	RAB26, member RAS oncogene family
511	204028_s_at	RABGAP1	10.647041	11.654987	-1.1191388	7.02E-09	RAB GTPase activating protein 1
512	202052_s_at	RAI14	12.39869	-13.204391	3.0853588	1.28E-09	retinoic acid induced 14
513	213049_at	RALGAPA1	16.000529	17.004326	-1.7879455	3.71E-11	Ral GTPase activating protein catalytic alpha subunit 1
514	214855_s_at	RALGAPA1///RALGA	16.142759	17.174404	-1.8821451	3.22E-11	Ral GTPase activating protein catalytic alpha subunit 1///Ral GTPase a
515	1553986_at	RASEF	12.133856	12.959026	-3.6197615	1.66E-09	RAS and EF-hand domain containing
516	230563_at	RASGEF1A	16.465084	17.566226	-4.670904	2.33E-11	RasGEF domain family member 1A
517	200997_at	RBM14-RBM4///RB	13.407725	14.178615	-1.059422	4.77E-10	RBM14-RBM4 readthrough///RNA binding motif protein 4
518	238447_at	RBMS3	12.056555	-12.888185	5.1211504	1.79E-09	RNA binding motif single stranded interacting protein 3
519	205407_at	RECK	21.137675	-24.441327	3.1344944	2.00E-13	reversion inducing cysteine rich protein with kazal motifs
520	205205_at	RELB	13.524143	-14.295199	1.9854199	4.26E-10	RELB proto-oncogene, NF-kB subunit

521	227758_at	RERG	13.245161	14.017302	-5.0595571	5.60E-10	RAS like estrogen regulated growth inhibitor
522	244745_at	RERG	10.569351	11.590096	-4.5648915	7.57E-09	RAS like estrogen regulated growth inhibitor
523	227339_at	RGMB	14.486639	-15.2944	1.3602611	1.66E-10	repulsive guidance molecule family member b
524	210138_at	RGS20	11.887574	-12.734531	3.6503875	2.11E-09	regulator of G-protein signaling 20
525	212099_at	RHOB	16.180339	17.219628	-3.8854469	3.10E-11	ras homolog family member B
526	1553962_s_at	RHOB	11.109732	12.047934	-2.2279887	4.48E-09	ras homolog family member B
527	60471_at	RIN3	13.155857	-13.929414	1.6438162	6.11E-10	Ras and Rab interactor 3
528	219457_s_at	RIN3	11.97617	-12.814886	1.0245206	1.93E-09	Ras and Rab interactor 3
529	214519_s_at	RLN2	17.08518	18.345848	-3.5560006	1.26E-11	relaxin 2
530	229285_at	RNASEL	14.447345	15.252333	-2.0248412	1.72E-10	ribonuclease L
531	214857_at	RPARP-AS1	10.771619	11.759689	-1.6754966	6.22E-09	RPARP antisense RNA 1
532	238026_at	RPL35A	11.922691	12.766328	-1.3122051	2.03E-09	ribosomal protein L35a
533	208456_s_at	RRAS2	11.074135	-12.017302	1.9113189	4.64E-09	related RAS viral (r-ras) oncogene homolog 2
534	203594_at	RTCA	13.96252	-14.742345	2.114359	2.77E-10	RNA 3'-terminal phosphate cyclase
535	205464_at	SCNN1B	10.367579	11.422993	-1.0531509	9.21E-09	sodium channel epithelial 1 beta subunit
536	218981_at	SDHAF3	25.925732	-34.944385	3.4899883	1.07E-15	succinate dehydrogenase complex assembly factor 3
537	223299_at	SEC11C	13.330345	14.101616	-1.142259	5.15E-10	SEC11 homolog C, signal peptidase complex subunit
538	203789_s_at	SEMA3C	12.948432	13.727245	-1.8751772	7.48E-10	semaphorin 3C
539	219259_at	SEMA4A	16.569061	17.694547	-1.3592171	2.11E-11	semaphorin 4A
540	201427_s_at	SEPP1	11.91298	12.757528	-6.5732804	2.05E-09	selenoprotein P, plasma, 1
541	200969_at	SERP1	10.930851	11.894687	-1.5086351	5.33E-09	stress-associated endoplasmic reticulum protein 1
542	202627_s_at	SERPINE1	20.697645	-23.681664	6.2096836	3.17E-13	serpin family E member 1
543	202628_s_at	SERPINE1	20.454558	-23.273556	5.0139585	4.08E-13	serpin family E member 1
544	205120_s_at	SGCB	14.902389	-15.746416	2.7400616	1.10E-10	sarcoglycan beta
545	228584_at	SGCB	12.201213	-13.021039	2.4099825	1.55E-09	sarcoglycan beta
546	226112_at	SGCB	10.883816	-11.854672	2.1494305	5.58E-09	sarcoglycan beta
547	201739_at	SGK1	10.631299	-11.641814	3.8333867	7.13E-09	serum/glucocorticoid regulated kinase 1
548	209090_s_at	SH3GLB1	15.130204	-15.99959	1.5723252	8.78E-11	SH3 domain containing GRB2 like endophilin B1
549	224817_at	SH3PXD2A	18.206784	-19.848553	2.9373625	4.07E-12	SH3 and PX domains 2A
550	243681_at	SHANK2	14.551934	15.364549	-3.2316524	1.55E-10	SH3 and multiple ankyrin repeat domains 2
551	213307_at	SHANK2	12.036097	12.869494	-3.1244243	1.82E-09	SH3 and multiple ankyrin repeat domains 2
552	242335_at	SLC25A37	20.228409	-22.901011	2.4630715	5.16E-13	solute carrier family 25 member 37
553	222528_s_at	SLC25A37	16.931977	-18.149988	2.7861069	1.47E-11	solute carrier family 25 member 37
554	218136_s_at	SLC25A37	13.991619	-14.77249	1.9673433	2.69E-10	solute carrier family 25 member 37
555	226179_at	SLC25A37	13.902541	-14.680395	2.3232386	2.94E-10	solute carrier family 25 member 37
556	221920_s_at	SLC25A37	12.996503	-13.773855	2.5463957	7.14E-10	solute carrier family 25 member 37
557	222529_at	SLC25A37	12.935687	-13.714911	2.522345	7.58E-10	solute carrier family 25 member 37
558	232092_at	SLC25A51	13.416148	-14.187019	1.3017131	4.73E-10	solute carrier family 25 member 51
559	219125_s_at	SLC50A1	13.601254	14.372915	-1.355083	3.95E-10	solute carrier family 50 member 1
560	201349_at	SLC9A3R1	16.713746	17.8747	-3.677347	1.82E-11	SLC9A3 regulator 1
561	209607_x_at	SLX1B-SULT1A4///S	10.959258	11.91891	-1.878766	5.19E-09	SLX1B-SULT1A4 readthrough (NMD candidate)///SLX1A-SULT1A3 rea
562	213139_at	SNAI2	15.258983	-16.144465	6.2458933	7.73E-11	snail family transcriptional repressor 2
563	229050_s_at	SNORA17B///SNOF	10.786276	11.772059	-3.1597541	6.14E-09	small nucleolar RNA, H/ACA box 17B///small nucleolar RNA, H/ACA l
564	209181_s_at	SNORD45C///SNOF	10.450053	-11.491048	1.1511676	8.50E-09	small nucleolar RNA, C/D box 45C///small nucleolar RNA, C/D box 4!
565	205573_s_at	SNX7	13.398255	-14.16917	5.4995341	4.82E-10	sorting nexin 7
566	1552269_at	SPATA17	12.415576	13.220178	-2.6934506	1.26E-09	spermatogenesis associated 17
567	214404_x_at	SPDEF	16.324949	17.394776	-3.2765973	2.68E-11	SAM pointed domain containing ETS transcription factor
568	220192_x_at	SPDEF	15.496578	16.415159	-3.8824207	6.11E-11	SAM pointed domain containing ETS transcription factor
569	243594_x_at	SPIRE2	11.629762	12.503242	-1.2572176	2.71E-09	spire type actin nucleation factor 2
570	203433_at	ST20-MTHFS///MTH	13.04682	13.8228	-1.1305997	6.80E-10	ST20-MTHFS readthrough///5,10-methenyltetrahydrofolate synthetas
571	223103_at	STARD10	11.297081	12.210267	-3.5480853	3.74E-09	StAR related lipid transfer domain containing 10
572	226525_at	STK17B	14.463338	-15.269442	2.5199852	1.69E-10	serine/threonine kinase 17b
573	213434_at	STX2	15.285077	-16.173978	1.3381317	7.53E-11	syntaxin 2
574	233841_s_at	SUDS3	12.741247	13.528011	-1.6963682	9.16E-10	SDS3 homolog, SIN3A corepressor complex component
575	203615_x_at	SULT1A1	17.338027	18.673893	-2.1524756	9.76E-12	sulfotransferase family 1A member 1
576	215299_x_at	SULT1A1	13.679418	14.452097	-2.3486092	3.66E-10	sulfotransferase family 1A member 1
577	211385_x_at	SULT1A2	11.294048	12.207623	-1.3539819	3.75E-09	sulfotransferase family 1A member 2
578	207122_x_at	SULT1A2	11.089497	12.030513	-1.172024	4.57E-09	sulfotransferase family 1A member 2
579	227352_at	SWSAP1	11.695546	12.561902	-1.4332428	2.54E-09	SWIM-type zinc finger 7 associated protein 1
580	44702_at	SYDE1	12.071294	-12.901665	1.7269294	1.76E-09	synapse defective Rho GTPase homolog 1
581	226068_at	SYK	18.717149	20.574961	-1.7815894	2.43E-12	spleen associated tyrosine kinase
582	235515_at	SYNE4	21.02516	24.244457	-3.1078907	2.26E-13	spectrin repeat containing nuclear envelope family member 4
583	212730_at	SYNM	20.743878	-23.760197	3.0701798	3.02E-13	synemin
584	229053_at	SYT17	13.622537	14.394434	-3.2214545	3.87E-10	synaptotagmin 17
585	205613_at	SYT17	12.897312	13.677837	-2.4387023	7.86E-10	synaptotagmin 17
586	227134_at	SYTL1	10.712629	11.710011	-3.3251762	6.59E-09	synaptotagmin like 1
587	213912_at	TBC1D30	28.164551	41.916378	-3.5771733	7.33E-17	TBC1 domain family member 30
588	213913_s_at	TBC1D30	23.381413	28.789327	-3.7460158	1.84E-14	TBC1 domain family member 30
589	221194_s_at	TBC1D3P1-DHX40P	15.465865	16.381062	-1.8735845	6.29E-11	TBC1D3P1-DHX40P1 readthrough, transcribed pseudogene///ring fin
590	234970_at	TC2N	26.506398	36.586864	-3.2495714	5.45E-16	tandem C2 domains, nuclear
591	1553132_a_at	TC2N	19.933817	22.425711	-4.3393997	6.99E-13	tandem C2 domains, nuclear
592	231104_at	TDRD5	17.14624	18.424517	-2.0115843	1.18E-11	tudor domain containing 5
593	205287_s_at	TFAP2C	11.211645	12.136004	-3.1051516	4.06E-09	transcription factor AP-2 gamma
594	204623_at	TFF3	14.948021	15.796811	-5.7037963	1.05E-10	trefoil factor 3
595	209651_at	TGFB1I1	16.025646	-17.034237	5.3717033	3.62E-11	transforming growth factor beta 1 induced transcript 1
596	35148_at	TJP3	15.961127	16.957509	-1.7631573	3.85E-11	tight junction protein 3
597	213412_at	TJP3	14.030658	14.813023	-1.7327656	2.59E-10	tight junction protein 3
598	223892_s_at	TMBIM4	20.028697	22.577578	-1.6803623	6.34E-13	transmembrane BAX inhibitor motif containing 4
599	222845_x_at	TMBIM4	19.978806	22.49758	-1.6480663	6.67E-13	transmembrane BAX inhibitor motif containing 4
600	226403_at	TMC4	16.186162	17.226646	-3.3884065	3.08E-11	transmembrane channel like 4
601	225822_at	TMEM125	15.07823	15.941482	-3.2821257	9.24E-11	transmembrane protein 125
602	213338_at	TMEM158	21.20505	-24.560094	5.9663916	1.87E-13	transmembrane protein 158 (gene/pseudogene)
603	227544_at	TMEM229B	16.325524	17.395476	-1.6972558	2.68E-11	transmembrane protein 229B
604	213285_at	TMEM30B	29.75992	48.203186	-5.7095771	9.30E-18	transmembrane protein 30B
605	228834_at	TOB1	19.416244	21.616979	-3.5936519	1.19E-12	transducer of ERBB2, 1
606	202704_at	TOB1	18.762183	20.640415	-3.1457045	2.32E-12	transducer of ERBB2, 1
607	221897_at	TRIM52	12.192444	13.01295	-1.6024003	1.56E-09	tripartite motif containing 52

608	217979_at	TSPAN13	16.433666	17.527638	-5.8931914	2.41E-11	tetraspanin 13
609	203226_s_at	TSPAN31	10.535354	11.561796	-1.5150637	7.83E-09	tetraspanin 31
610	225388_at	TSPAN5	15.473501	-16.388671	2.6367413	6.25E-11	tetraspanin 5
611	225387_at	TSPAN5	12.628699	-13.420897	2.6703998	1.02E-09	tetraspanin 5
612	1554588_a_at	TTC30B	12.657468	13.448203	-1.7696486	9.94E-10	tetratricopeptide repeat domain 30B
613	243413_at	TTC30B	11.335253	12.243574	-1.7908183	3.60E-09	tetratricopeptide repeat domain 30B
614	210652_s_at	TTC39A	23.084172	28.162834	-5.2083505	2.54E-14	tetratricopeptide repeat domain 39A
615	219415_at	TTYH1	13.659799	14.432184	-1.5418903	3.73E-10	tweety family member 1
616	204141_at	TUBB2A	12.910279	-13.690354	4.096885	7.77E-10	tubulin beta 2A class IIa
617	209372_x_at	TUBB2B///TUBB2A	15.820161	-16.791071	1.9204475	4.43E-11	tubulin beta 2B class IIb///tubulin beta 2A class IIa
618	209191_at	TUBB6	16.291611	-17.354238	2.7597603	2.78E-11	tubulin beta 6 class V
619	205694_at	TYRP1	24.257558	30.739273	-5.0840875	7.04E-15	tyrosinase related protein 1
620	209115_at	UBA3	11.188044	-12.115559	0.961292	4.16E-09	ubiquitin like modifier activating enzyme 3
621	238462_at	UBASH3B	15.25894	-16.144417	3.9638467	7.73E-11	ubiquitin associated and SH3 domain containing B
622	225982_at	UBTF	10.902256	11.870346	-1.113833	5.48E-09	upstream binding transcription factor, RNA polymerase I
623	214843_s_at	USP33	19.43834	-21.650841	1.2343894	1.16E-12	ubiquitin specific peptidase 33
624	212513_s_at	USP33	12.593836	-13.387874	0.9856912	1.06E-09	ubiquitin specific peptidase 33
625	209946_at	VEGFC	16.61844	-17.755822	3.7571197	2.00E-11	vascular endothelial growth factor C
626	201426_s_at	VIM	31.374398	-56.249142	7.7055969	9.46E-19	vimentin
627	200611_s_at	WDR1	12.389825	-13.196111	1.8512587	1.29E-09	WD repeat domain 1
628	210935_s_at	WDR1	11.31274	-12.22392	2.1971063	3.68E-09	WD repeat domain 1
629	203855_at	WDR47	12.016351	-12.851478	1.2461254	1.86E-09	WD repeat domain 47
630	213031_s_at	WDR73	13.900778	14.678577	-1.6823343	2.94E-10	WD repeat domain 73
631	221029_s_at	WNT5B	10.550815	-11.574659	4.2621781	7.71E-09	Wnt family member 5B
632	225665_at	ZAK	15.16391	-16.037384	2.924636	8.49E-11	sterile alpha motif and leucine zipper containing kinase AZK
633	225662_at	ZAK	13.996597	-14.777652	3.2226188	2.68E-10	sterile alpha motif and leucine zipper containing kinase AZK
634	223519_at	ZAK	12.014268	-12.849579	3.058014	1.86E-09	sterile alpha motif and leucine zipper containing kinase AZK
635	212774_at	ZBTB18	23.42833	-28.889773	5.5415922	1.75E-14	zinc finger and BTB domain containing 18
636	227162_at	ZBTB26	14.147843	14.935332	-1.4742206	2.31E-10	zinc finger and BTB domain containing 26
637	228216_at	ZBTB37	14.032519	14.814958	-1.4220669	2.59E-10	zinc finger and BTB domain containing 37
638	226962_at	ZBTB41	11.92737	12.77057	-1.4184288	2.03E-09	zinc finger and BTB domain containing 41
639	229691_at	ZBTB42	13.8397	14.615754	-3.0928603	3.13E-10	zinc finger and BTB domain containing 42
640	222731_at	ZDHHC2	25.839152	-34.708095	4.9558584	1.18E-15	zinc finger DHHC-type containing 2
641	222730_s_at	ZDHHC2	10.38364	-11.436219	3.2227036	9.07E-09	zinc finger DHHC-type containing 2
642	212764_at	ZEB1	25.01655	-32.565601	6.4264317	3.02E-15	zinc finger E-box binding homeobox 1
643	210875_s_at	ZEB1	17.356636	-18.698276	4.7004931	9.57E-12	zinc finger E-box binding homeobox 1
644	210275_s_at	ZFAND5	17.76306	-19.239171	1.2399169	6.36E-12	zinc finger AN1-type containing 5
645	217741_s_at	ZFAND5	13.964627	-14.744526	1.2949786	2.77E-10	zinc finger AN1-type containing 5
646	1555982_at	ZFYVE16	11.264595	12.181983	-2.1653668	3.86E-09	zinc finger FYVE-type containing 16
647	228058_at	ZG16B	28.28397	42.342668	-3.2163368	6.32E-17	zymogen granule protein 16B
648	206314_at	ZKSCAN7	12.457012	-13.258987	1.1421062	1.21E-09	zinc finger with KRAB and SCAN domains 7
649	202136_at	ZMYND11	11.382397	12.284821	-1.373784	3.44E-09	zinc finger MYND-type containing 11
650	244743_x_at	ZNF138	10.683127	11.685234	-1.9887498	6.78E-09	zinc finger protein 138
651	215022_x_at	ZNF33B	11.597386	12.474461	-2.5430855	2.79E-09	zinc finger protein 33B
652	227952_at	ZNF595	12.816457	13.600026	-5.1886339	8.51E-10	zinc finger protein 595
653	224593_at	ZNF664	11.96133	12.801395	-1.5811079	1.96E-09	zinc finger protein 664
654	243729_at		21.082873	24.34521	-2.7074102	2.12E-13	
655	231042_s_at		19.607906	-21.912639	3.4687254	9.77E-13	
656	232113_at		19.317958	-21.467066	4.9508479	1.31E-12	
657	238646_at		16.440434	17.535944	-2.1786312	2.39E-11	
658	229223_at		16.072601	17.090296	-3.1584458	3.45E-11	
659	233364_s_at		14.286613	-15.081416	3.7924479	2.02E-10	
660	238946_at		14.069095	14.853036	-1.3587647	2.50E-10	
661	242354_at		14.038138	14.820802	-2.6862163	2.57E-10	
662	227943_at		12.616979	13.409787	-3.1443151	1.03E-09	
663	239536_at		12.432501	13.236018	-1.6090358	1.24E-09	
664	242909_at		12.26152	13.076787	-2.5776077	1.46E-09	
665	236113_at		11.893441	12.739839	-1.6868543	2.09E-09	
666	242874_at		11.446432	12.341039	-1.9648303	3.23E-09	
667	236445_at		11.255899	12.174422	-1.8635995	3.89E-09	
668	235247_at		11.114719	12.05223	-3.5009944	4.46E-09	
669	1556938_a_at		10.865967	11.839518	-1.2398735	5.68E-09	
670	244475_at		10.70015	11.699524	-1.565064	6.67E-09	
671	230795_at		10.405261	11.454045	-3.697192	8.88E-09	
672	241429_at		10.376615	11.430432	-1.0174526	9.13E-09	
